# A thiochromenone antibiotic derived from the *Pseudomonas* quinolone signal selectively targets the Gram-negative pathogen *Moraxella catarrhalis*†
†Electronic supplementary information (ESI) available. See DOI: 10.1039/c9sc01090d


**DOI:** 10.1039/c9sc01090d

**Published:** 2019-05-23

**Authors:** Dávid Szamosvári, Tamara Schuhmacher, Christof R. Hauck, Thomas Böttcher

**Affiliations:** a Department of Chemistry , Konstanz Research School Chemical Biology , Zukunftskolleg , University of Konstanz , 78457 Konstanz , Germany . Email: Thomas.Boettcher@uni-konstanz.de; b Department of Biology , University of Konstanz , 78457 Konstanz , Germany . Email: Christof.Hauck@uni-konstanz.de

## Abstract

The *Pseudomonas* quinolone signal (PQS) is an important quorum sensing signal of the pathogen *Pseudomonas aeruginosa*. We discovered an additional activity of PQS as a narrow spectrum antibiotic. Exploiting the privileged structure of PQS by the synthesis of heteroatom-substituted analogues led to a class of 2-alkyl-3-hydroxythiochromen-4-ones with highly potent antibiotic activity against the nasopharyngeal pathogen *Moraxella catarrhalis*. Synthetic optimization resulted in minimum inhibitory concentrations in the nanomolar range even for clinical isolates of *M. catarrhalis*. Surprisingly, the growth of other human pathogens and commensals, including closely related *Moraxella* species, was not inhibited, indicating exceptional species selectivity. Mechanistic studies revealed that the antibiotic was bactericidal and likely inhibits a target in the primary energy metabolism causing rapid depletion of the cellular ATP pool.

## Abstract 2

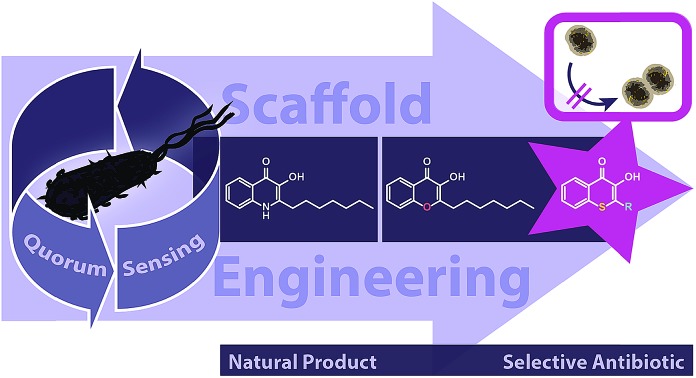
Scaffold engineering of the *Pseudomonas* quinolone signal results in a highly potent antibiotic with unprecedented species selectivity.

## 

Antibiotic-resistant pathogens limit the treatment of infectious diseases and are a major threat for human health. Thus, the development of novel antibiotics and expansion of the target spectrum are of utmost urgency.^1^ However, the discovery of new classes of potent antibiotics is rare, especially against Gram-negative bacteria, which are intrinsically harder to kill and often use multiple redundant resistance mechanisms.^2^ We have recently investigated the class of 2-alkyl-4-quinolone N-oxides of *Pseudomonas aeruginosa* and found an unsaturated compound as a major antibiotic weapon against *Staphylococcus aureus*.^3^,^4^ Other quinolones like the *Pseudomonas* quinolone signal (PQS) and its biosynthetic precursor 2-heptyl-4(1*H*)-quinolone (HHQ) are quorum sensing signals that coordinate the expression of virulence factors in *P. aeruginosa*.^5^ However, additional roles of these compounds have also been reported including growth inhibitory effects of HHQ against *S. aureus* and *Vibrio* species,^6^,^7^ as well as trapping of ferric iron^8^,^9^ and the formation of outer membrane vesicles by PQS.^10^,^11^ Activity-based protein profiling with photoreactive versions of HHQ and PQS revealed numerous putative targets and protein interactions in *P. aeruginosa* which indicate additional roles beyond classical signaling.^12^ In addition, PQS has been implicated in impairing the human immune response.^13^ We reasoned that PQS might have further unexplored effects in interspecies interactions and thus investigated potential antibiotic effects of PQS on various Gram-negative pathogens, which may compete with *P. aeruginosa* in the human body.

Here we report a highly selective antibacterial effect of the quorum sensing signal PQS of *Pseudomonas aeruginosa* against the human pathogen *Moraxella catarrhalis*, which we further exploited by organic synthesis. We hereby developed a new class of antibiotics based on a thiochromenone scaffold that exhibited submicromolar activity and unprecedented species selectivity by targeting the primary metabolism of *M. catarrhalis*.

We synthesized PQS (**1**) by the cyclization of 2-oxononyl 2′-aminobenzoate^14^ and tested it on the growth of 10 different species (Fig. S1†). *Enterobacteria* such as *Klebsiella pneumoniae*, *Enterococcus faecalis*, and *Escherichia coli* appeared to be unaffected by PQS concentrations of up to 50 μM. In contrast, the growth of *Moraxella catarrhalis* and *Neisseria meningitidis* was significantly impaired at concentrations as low as 5 μM (Fig. 1).

**Fig. 1:**
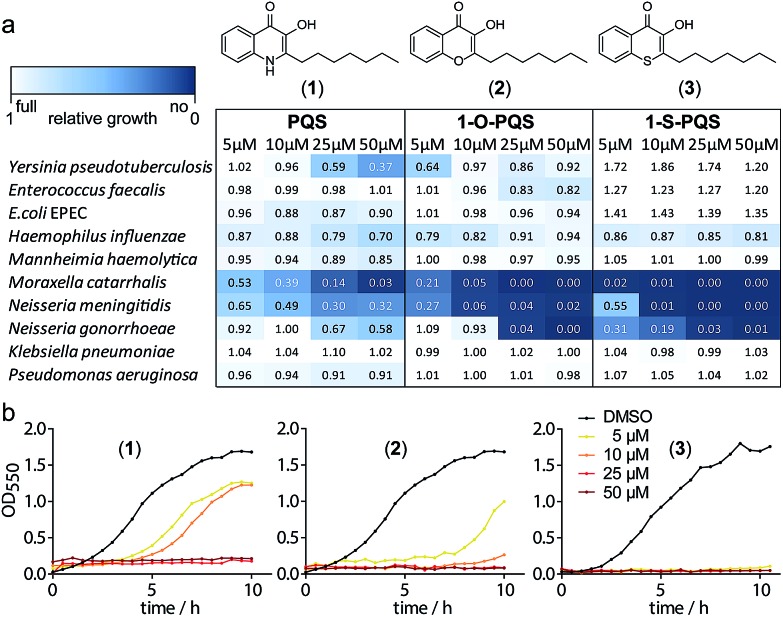
Antibacterial activity of the natural product PQS (**1**) and the synthetic derivatives (**2**) and (**3**) against Gram-negative bacteria. (a) Integrals of growth curves color-coded from dark blue (no growth) to white (full growth). (b) Example growth curves for the three compounds with *M. catarrhalis* ATCC 25238.

In cultures of *P. aeruginosa*, PQS is produced in multi-micromolar quantities,^15^ suggesting that inhibiting the growth of certain competing species may be one additional, signaling-unrelated role of PQS. Next, we aimed to exploit this surprisingly selective activity of native PQS against certain Gram-negative bacteria by evolving a scaffold of this privileged structure using synthetic chemistry. In the first step, we generated heteroatom-substituted PQS derivatives with a 3-hydroxychromen-4-one and a 3-hydroxythiochromen-4-one scaffold. Chromen-4-one (**2a**) was synthesized by esterification of 2-hydroxyacetophenone with octanoyl chloride followed by Baker–Venkataraman rearrangement.^16^ 1-O-PQS (**2**) was obtained after epoxidation of chromen-4-one (**2a**) with *tert*-butyl hydroperoxide and subsequent arene oxide rearrangement.^17^ The related 1-S-PQS (**3**) was synthesized from thiophenol and *trans*-2-decenoic acid in a superacid-catalyzed alkylation followed by a cyclic acylation reaction to give thiochroman-4-one (**3b**),^18^ which was subsequently oxidized by nitrosation with isoamyl nitrite and ensuing oximation and oxime hydrolysis (Scheme 1).^19^

**Scheme 1:**
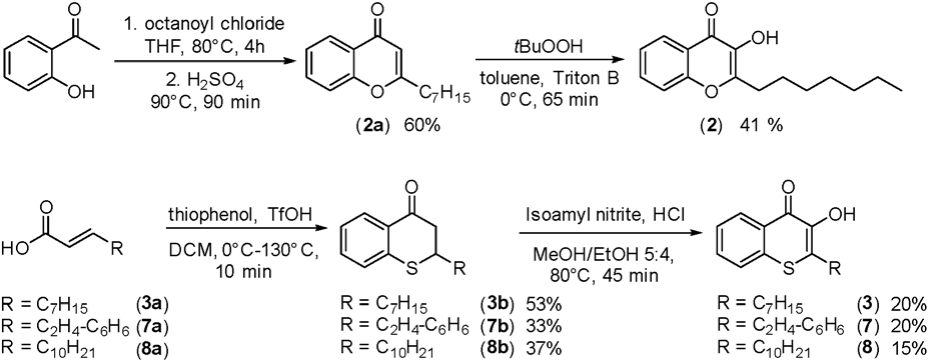
Synthesis of chromenone (**2**) and thiochromenones (**3**, **7**, and **8**).

Alkylated 3-hydroxychromen-4-one (**2**) and 3-hydroxythiochromen-4-one (**3**) exhibited both significantly stronger growth inhibition compared to PQS and an identical species spectrum (Fig. S1†). 3-Hydroxythiochromen-4-one (**3**) was hereby the most active compound and completely inhibited the growth of *M. catarrhalis* at concentrations down to 5 μM (Fig. 1). We therefore synthesized a small focused library of substituted thiochromen-4-one derivatives. To test whether 3-OH was essential for the activity, we generated the HHQ derived thiochromen-4-one compound (**4**).^20^ Interestingly, the lack of the 3-OH group resulted in a severe drop in activity against *M. catarrhalis* so further refinement was restricted to the 3-hydroxythiochromen-4-one scaffold (Fig. 2a and b). Since *Pseudomonas* quinolone N-oxides are known respiratory chain inhibitors,^3^,^21^,^22^ we investigated the possibility that (**3**) is activated *in situ* by oxidation to a sulfoxide, which is isosteric with N-oxide. We thus synthesized thiochromen-4-one 1-oxide (**5**) and 1,1-dioxide (**6**) by treating (**3**) with mCPBA (Scheme 2). Both compounds with an oxidized sulfur exhibited strongly decreased activity against *M. catarrhalis* suggesting that (**3**) is not activated by 1-S-oxidation (Fig. 2b). We next explored the effects of variations on substitution and electronic changes to the thiochromen-4-one ring system. 2-Phenethyl (**7**) and 2-decyl (**8**) derivatives were synthesized using the same strategy as for (**3**). To obtain the thiopyranopyridin-4-ones (**9** and **10**) we used the described oxidation method on 2-heptyl-2,3-dihydro-4*H*-thiopyrano[2,3-*b*]pyridin-4-one (**9c**) and 2-heptyl-2,3-dihydro-4*H*-thiopyrano[3,2-*c*]pyridin-4-one (**10c**), which were synthesized according to Kobayashi *et al.* (Scheme 2).^23^ Derivatives **11–14** with substitutions at the aromatic region were not accessible by this synthetic approach. We therefore used copper catalyzed conjugate addition of heptylmagnesium bromide on the corresponding thiochromenones (**11c–14c**) as described by Luo *et al.*^24^ to obtain 2-heptylthiochroman-4-ones (**11d–14d**) in four steps. Riley oxidation with SeO_2_ gave 2-heptyl-3-hydroxythiochromen-4-ones (**11–14**) with less byproducts compared to oxidation with isoamyl nitrite (Scheme 2).

**Fig. 2:**
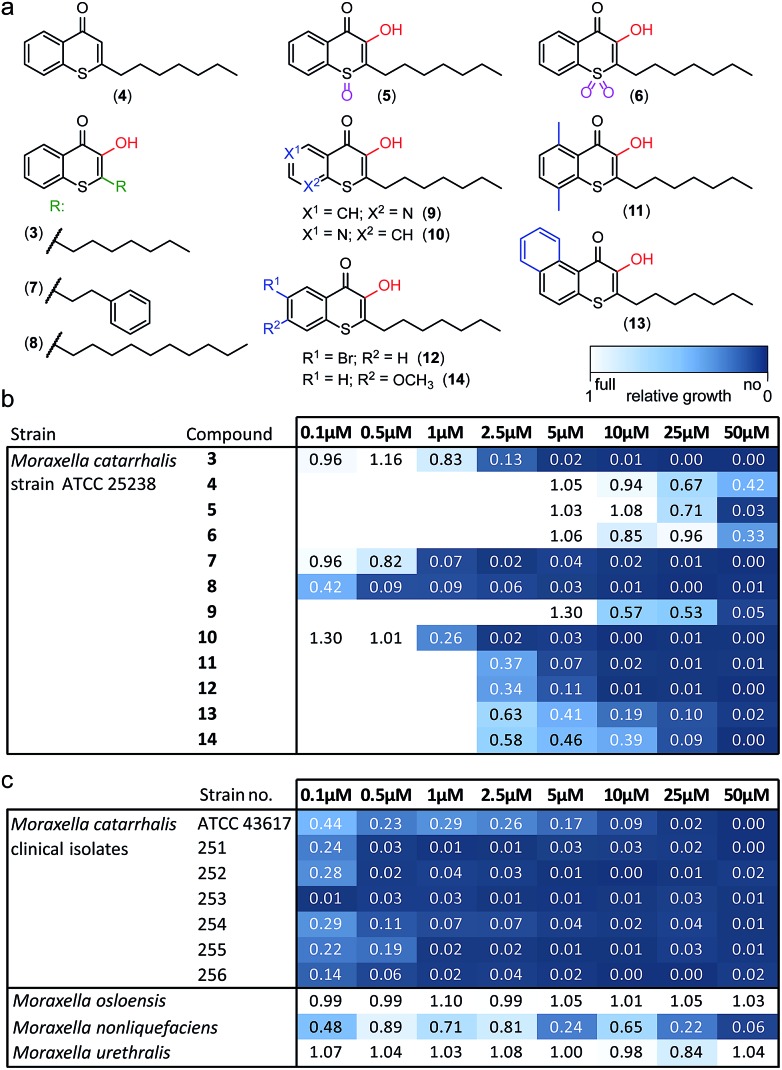
Derivatives of 1-S-PQS and their activity against *Moraxella*. (a) Focused synthetic library. (b) Antibacterial activity against *M. catarrhalis* ATCC 25238. (c) Activity of compound (**8**) against clinical strains and commensal *Moraxella* species. Integrals of growth curves color-coded from dark blue (no growth) to white (full growth).

**Scheme 2:**
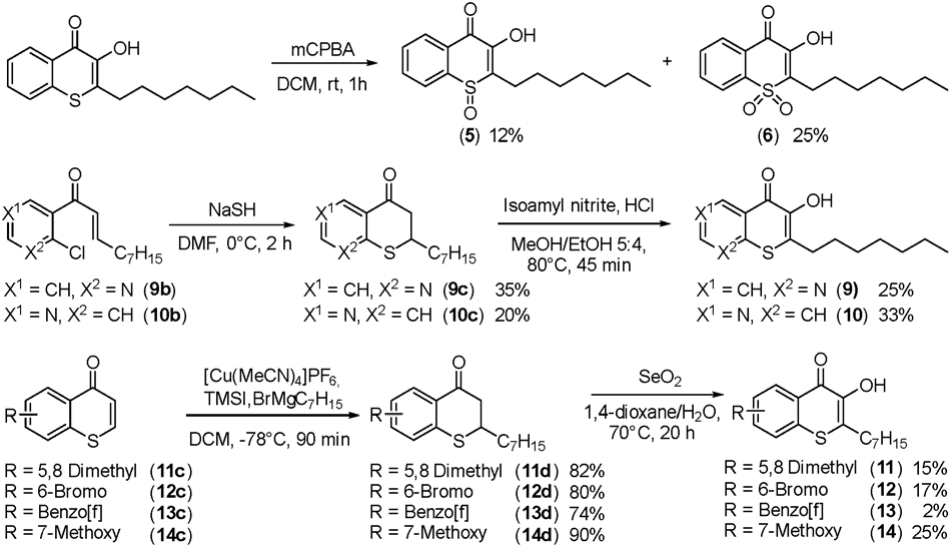
Synthesis of thiochromenones (**5**, **6** and **9–14**).

The phenethyl group of (**7**) increased the activity by more than two-fold compared to the parent compound (**3**). Strikingly, the decyl chain (**8**) further boosted the activity against *M. catarrhalis* by a factor of ten, while the thiopyranopyridin-4-one (**10**) slightly increased and (**9**) nearly completely abrogated the activity as compared to (**3**). Aromatic ring substitutions (**11** and **12**) were tolerated with a minor loss of activity, while (**13**) and (**14**) led to a four- to ten-fold drop in activity (Fig. 2b and S2†). None of the compounds displayed major activities against other nasopharyngeal pathogens such as *Haemophilus influenzae* or *Streptococcus pyogenes* (Fig. S3†). Since the most potent compound (**8**) in this study displayed unprecedented activity against the human pathogen *M. catarrhalis* in the nanomolar range with a MIC of only 0.5 μM (∼0.16 μg mL^–1^), we investigated if this compound also inhibited the growth of more problematic clinical isolates ranging from blood cultures to wound swabs and tracheal exudates. Indeed all clinical strains were inhibited with MIC values ranging from <0.1 μM to 1 μM (Fig. 2c and S4†). The antibiotic activity of (**8**) was hereby highly selective for *M. catarrhalis* and even the closely related commensal species *Moraxella osloensis* and *Moraxella urethralis* did not respond to the antibiotic at concentrations of up to 50 μM (Fig. 2c and S5†).

This unexpected selectivity indicates an extremely narrow-spectrum antibiotic, which is rare especially for Gram-negative pathogens. In order to test the cytotoxicity of our lead compound (**8**) against eukaryotic cells, we measured the metabolic activity and membrane integrity of A549 human lung carcinoma cells.

Adverse effects on metabolism were only observed at around 50 μM and loss of membrane integrity was found only after a prolonged incubation of 2–3 days at 50 μM, indicating that the sensitivity of eukaryotic cells is two orders of magnitude lower than the MIC against *M. catarrhalis* (Fig. S6a and b†). A similarly low sensitivity was observed for human kidney cells (Fig. S6c†). These properties make our lead compound an interesting and highly potent drug candidate for treating infections caused by *M. catarrhalis* including middle-ear infections in children as well as pneumonia, endocarditis, septicemia, and meningitis in adults.^25^*Moraxella* is also one of the few pathogens associated with exacerbations of chronic obstructive pulmonary disease, a pathologic lung condition of growing global significance.^26^ We thus aimed to further investigate the molecular mechanism by which the compound inhibits the growth of *M. catarrhalis*.

Since the 3-OH group was essential for the activity and the natural product PQS has a role in ferric iron trapping, we investigated the possible interference of (**8**) with *Moraxella*'s iron uptake. While pre-incubation of (**8**) in an equimolar ratio with ferric chloride abrogated antibiotic activity, supplementing bacterial cultures separately with (**8**) and ferric iron restored growth inhibition (Fig. 3c). These results suggest that the catechol moiety of 3-hydroxythiochromene-4-one may be important for interactions with the cellular target and consequently a pre-formed complex with ferric iron would prevent target binding. The structurally related iron chelator deferiprone had no effect on the growth of *M. catarrhalis* even at 100 μM (Fig. 3c), confirming that iron chelation was not responsible for the antibiotic activity. Measuring membrane polarization using a fluorescent indicator dye showed no significant influence of compound (**8**) within two hours, while the proton ionophore CCCP or the cytochrome c reductase inhibitor antimycin A abrogated the membrane potential within 15 min (Fig. S7†). Also, no increase in reactive oxygen species (ROS) was detected (Fig. S8†). Scanning electron micrographs of *M. catarrhalis* cells treated with and without our antibiotic (**8**) did not show visible morphological differences compared to a known inhibitor of cell wall biosynthesis, suggesting that the membrane and cell wall of the bacteria were not targets of the compound (Fig. 3a). To investigate if DNA, RNA, or protein biosynthesis were targeted by the compound, we analyzed the incorporation of ^3^H radiolabeled precursors of the corresponding biopolymers. Control antibiotics known to interfere with DNA replication, transcription, or protein biosynthesis selectively prevented the incorporation of the radiolabeled precursors. In the samples treated for 40 min with (**8**), incorporation of the corresponding ^3^H labelled precursors was inhibited for each of the three biopolymers (Fig. 3b), indicating an effect on the primary metabolism of the cell, which ultimately affects biosynthesis of all macromolecules.

**Fig. 3:**
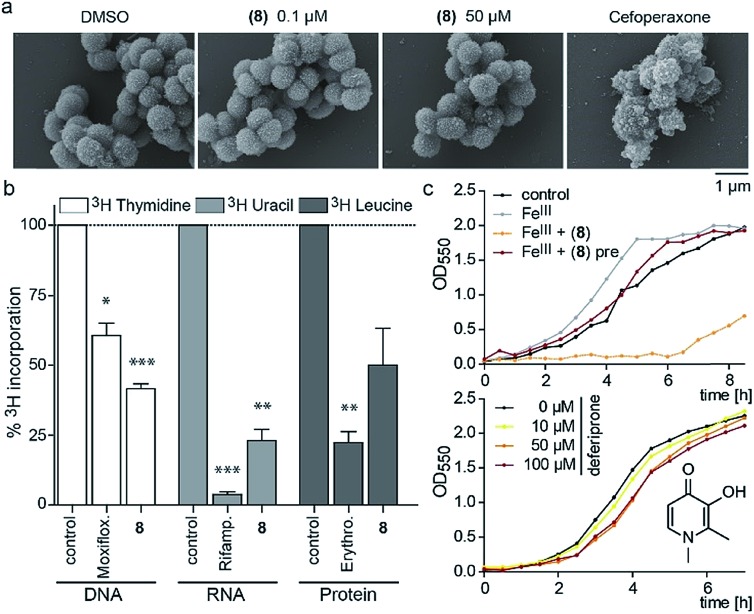
Morphological and physiological responses of *M. catarrhalis* ATCC 25238 to compound (**8**). (a) Scanning electron micrographs of cells after treatment with (**8**) in comparison to a DMSO control and the cell wall inhibitor cefoperaxone. (b) Radiolabelling experiments with ^3^H-thymidine (DNA), ^3^H-uridine (RNA), ^3^H-leucine (protein) and antibiotics moxifloxacin, rifampicin, and erythromycin as positive controls. (c) Upper panel: growth of *M. catarrhalis* with ferric iron (Fe^III^, 5 μM) and 5 μM compound (**8**); pre: pre-incubation. Lower panel: effect of the iron chelator deferiprone on the growth of *M. catarrhalis*.

To investigate the survival rate of cells, we challenged the bacteria with (**8**) for 40 min and 3 h and then plated dilutions on antibiotic-free agar for counting of colony forming units (CFUs). 40 min of exposure already severely reduced the viability of the cells (Fig. 4a and S9†), which led us to conclude that (1) our compound is bactericidal and not bacteriostatic and (2) the antibiotic inhibits a pathway, which causes rapid cell death. Hereby the average colony size on the plates considerably decreased when pre-incubated with higher concentration of (**8**) (Fig. 4b). Small-colony morphology is typically associated with impaired electron transport and depletion of ATP.^27^ Indeed, the overall metabolic activity of *M. catarrhalis* as measured by the reduction of a tetrazolium salt was inhibited (Fig. 4c). We thus investigated if cellular ATP concentrations were affected by our antibiotic using a luciferase assay. After 20 min of exposure, ATP levels in *M. catarrhalis* had already dropped significantly depending on the concentration of (**8**). In contrast, ATP levels in *M. osloensis*, a closely related microbe not sensitive to (**8**), did not decrease (Fig. 4d). These results suggest that our thiochromenone antibiotic (**8**) inhibits a pathway in the energy metabolism of *M. catarrhalis* in a highly effective manner with remarkable species specificity. Continuous cultivation of *M. catarrhalis* ATCC 25238 at 0.1 μM of compound (**8**) for 10 days did not result in decreased susceptibility, suggesting that the compound was not prone to rapid resistance development (Fig. S10†).

**Fig. 4:**
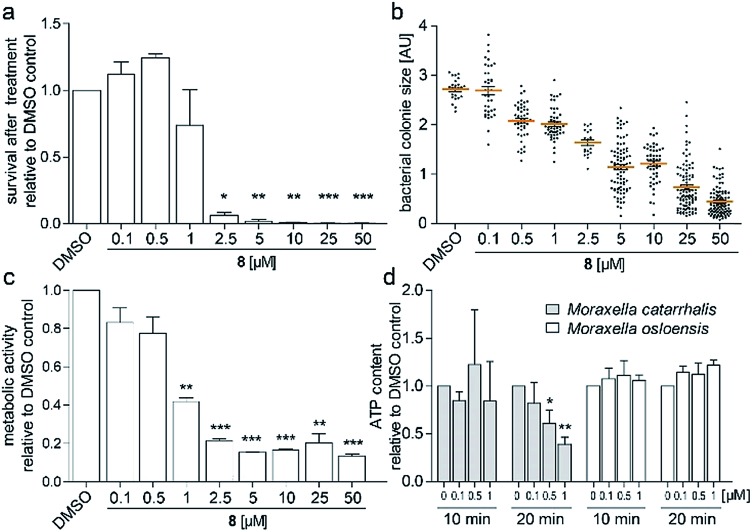
(a) Survival of *M. catarrhalis* ATCC 25238 after treatment for 40 min with (**8**) shown by the number of CFUs on agar relative to the DMSO control. (b) Relative sizes of the colonies quantified in (a). (c) Inhibition of the metabolic activity of *M. catarrhalis* after 3 h of incubation with (**8**). (d) Rapid concentration dependent depletion of ATP after 20 min of incubation with (**8**) for *M. catarrhalis* but not for *M. osloensis*.

3-Hydroxythiochromen-4-ones in this study constitute a novel class of highly potent narrow spectrum antibiotics. Related quinolones like nalidixic acid or fluoroquinolones have comparable potency but lack this species selectivity.^28^

While these quinolone antibiotics are topoisomerase inhibitors, 3-hydroxythiochromen-4-ones did not selectively inhibit DNA replication and further mechanistic investigations point to primary energy metabolism as the target. This new class of antibiotics may pave the way for customized drugs against infectious diseases or serve as chemical precision tools for microbiome research.

In conclusion, we were able to exploit the selective antibacterial activity of the quorum sensing signal PQS against the Gram-negative pathogen *Moraxella catarrhalis* by developing a new class of highly potent thiochromenone antibiotics with unprecedented species selectivity. The antibiotic targets primary energy metabolism causing rapid depletion of the cellular ATP pool. Our results demonstrate that (1) quorum sensing signals like PQS can have roles beyond classical signaling and (2) even moderate activities of these natural products can be exploited by scaffold engineering and lead to highly potent antibiotics with unprecedented customized species selectivity against medically relevant Gram-negative pathogens.

## Conflicts of interest

There are no conflicts to declare.

## Supplementary Material

Supplementary information
