# Supplementary material for: A thiochromenone antibiotic derived from the Pseudomonas quinolone signal selectively targets the Gram-negative pathogen Moraxella catarrhalis
Source: Chem Sci. 2019 May 23;10(27):6624–8. doi: 10.1039/c9sc01090d (PMC6624978; doi:10.1039/c9sc01090d)
Supplement: Supplementary file 1 [file SC-010-C9SC01090D-s001.pdf]

Electronic Supplementary Material (ESI) for Chemical Science.
This journal is © The Royal Society of Chemistry 2019

A thiochromenone antibiotic derived from Pseudomonas quinolone signal selectively targets the Gram-negative pathogen *Moraxella catarrhalis*

Dávid Szamosvári[‡a], Tamara Schuhmacher[‡b], Christof Hauck[b*], Thomas Böttcher[a*]

[a]Department of Chemistry, Konstanz Research School Chemical Biology, Zukunftskolleg, University of Konstanz, 78457 Konstanz

[b]Department of Biology, University of Konstanz, 78457 Konstanz

1. Biological Methods

Growth of bacterial strains

Yersinia pseudotuberculosis, Moraxella catarrhalis and Neisseria strains were cultured in PPM medium (15 g/L Proteon Pepton, 1 g/L soluble starch, 5 g/L NaCl, 4 g/L KH_2PO_4, 1 g/L K_2HPO_4, pH 7.5) for liquid culture and on GC plates (BD Difco™ GC Medium Base) supplemented with 1 % vitamin mix (100 g/L glucose, 10 g/L glutamine, 25.9 g/L L-cystein, 100mg/L cocarboxylase (thiamine pyrophosphate), 250 mg/L NAD, 500 µL/L $Fe(NO_3)_3$, 1.1 g/L L-cystin, 150 mg/L arginine, 3 mg/L thiamine-HCl 10 mg/L vitamine B12, 13 mg/L *p*-amino benzoic acid, 1 g/L adenine, 500 mg/L uracil, 30 mg/L guanine). Strains were either cultivated at 37°C and 220 rpm (liquid) or at 37°C and 5 %CO_2 (plates).

Enterococcus faecalis, *Haemophilus influenza*e and *Mannheimia haemolytica*, as well as *Moraxella osloenis*, *nonliquefaciens* and *urethralis* were grown in BHI medium (BD Difco Brain Heart Infusion) with 36 g/L supplemented with 20 µg/mL hemin, 20 µg/mL NAD and 15 g/L agar for plates.

Streptococcus pyogenes was grown in trypticase soy yeast extract medium (30 g/L trypticase soy broth, 3 g/L yeast extract, 15 g/L agar for plates; pH 7.0-7.2)

Escherichia coli strains, *Pseudomonas aeruginosa* and *Klebsiella pneumoniae* were cultured in LB medium (10 g/L tryptone, 5 g/L yeast extract, 5 g/L NaCl, 15 g/L agar for plates; pH 7.0).

Bacteria were streaked out on plate as described above and incubated overnight. 2-3 h prior to the experiment the bacteria were pre-cultured in liquid medium, then harvested and resuspended in PBS. Optical density was determined and a volume equal to OD_{550} = 0.2 (*Yersinia*, *Moraxella*, *Neisseria*) or OD_{600} = 0.2 (*Enterococcus*, *Haemophilus*, *Mannheimia*, *Streptococcus*), was inoculated into 5mL of liquid medium. Compounds were added as indicated. DMSO level was adjusted to 1 % of final concentration. Samples were incubated for either 10.5 hours, until they reached stationary phase (three measured values in stationary phase) or until they reached an OD of 2.5. Optical density was determined every 0.5 h.

For comparison of different growth curves, area under the curve (AUC) values were determined, blanked to their initial OD and normalized to the DMSO control. An overview of all strains used in this study is given in Table 1.

Growth monitoring with Fe^{3+}/Deferiprone

Incubation with iron was conducted under the same growth conditions described above. An aqueous solution of 5 µM $FeCl_3$ was added to the medium directly prior to the experiment. The ferric iron chelator deferiprone was added at 0, 10, 50 or 100 µM concentration directly before the cultivation.

Survival assay / colony size evaluation

Moraxella catarrhalis strain ATCC 25238 was grown on GC agar over night at 37°C and 5 % CO_2. Bacteria were harvested and resuspended in fresh 5 mL BHI medium and cultivated for 4 h at 37°C and 250 rpm. Optical density was measured at 550 nm; 10^8 bacteria were added to 5 mL BHI medium with the following concentrations of compound **8**: 0.1, 0.5, 1, 2.5, 5, 10, 25, 50 µM or DMSO (1%) or control without treatment. DMSO was added in each sample except control to equal 1 % of final volume. Bacteria were incubated for 40 min or 3 h at 37 °C and 250 rpm.
For survival assay: Bacteria were diluted from 10^{-1} to 10^{-4} in PBS. 20 µL of every dilution were plated on GC agar, incubated over night at 37 °C and 5 % CO_2, before bacterial colonies were quantified.
Colony size evaluation: Plates were scanned and area of single grown colonies was measured using the FIJI distribution of ImageJ 1.52e.

ATP assay

Moraxella spec. were incubated with compound **8** as described above for 10 or 20 minutes respectively. Bacteria were harvested through centrifugation for 10 min at 4000 rpm and 4 °C, resuspended in 1 mL PBS and frozen overnight at -80°C. Samples were sonicated for 1 min at maximum intensity on ice. Supernatant was cleared by centrifugation for 10 min at 13,500 rpm and 4°C. ATP assay was performed using 10 µL of supernatant, 100 µL ATP assay buffer (25 mM Tris pH 7.8, 4 mM EGTA, 20 mM $MgSO_4$, 1 mM DTT, pH 7.8), 20 µmol luciferin and 1 µg luciferase (recombinant, $\geq 10 \cdot 10^{10}$ units/mg protein, Sigma-Aldrich) per well. Luminescence was measured in white 96 well plates using a Thermo Fisher Varioskan Flash spectrophotometer.

MTT assay for human cells

A549 cells have been cultivated in Dulbecco's modified Eagle's medium (DMEM) supplemented with 10 % fetal calf serum and passaged every 2-3 days.
HEK293T cells were cultured on gelatine coated cell culture dishes using DMEM supplemented with 10 % calf serum, and passaged every 2-3 days. Both lines were kept at 37 °C and 5 % CO_2.
All plates were coated overnight at 4 °C with 0.1 % gelatine in PBS before usage. $2 \cdot 10^4$ A549 or HEK293T cells respectively were incubated with the mentioned concentrations of compound **8** in 100 µL of their respective medium and incubated for 1, 2 and 3 days in the case of A549 or 1 day for HEK293T at 37°C and 5% CO_2 in a 96 well plate. 10 µL of MTT solution (12 mM in PBS, sterile filtered with 0.2 µM filter) were added. The cells were subsequently incubated for 2 h at 37 °C. Afterwards growth medium was removed and 100 µL isopropanol was added to each well. The formazan was allowed to dissolve overnight in a humidified chamber at room temperature and quantified by OD_{550} measurement using a Thermo Fisher Varioskan Flash spectrophotometer.

MTT assay for bacteria

Bacteria were cultivated and treated with compound **8** as described above. After the incubation time, 100 µl of the bacteria solution were mixed with 500 µl 12mM MTT in PBS and incubated for 1 h at 37°C and 250 rpm. Bacteria were then centrifuged for 20 min at 4700 rpm at RT. The supernatant was discarded and the pellet was dissolved in 2 ml isopropanol overnight. OD_{550} was measured using a Thermo Fisher Varioskan Flash spectrophotometer.

PI assay

$5 \cdot 10^5$ A549 cells were incubated with indicated amounts of compound **8** in 1mL medium for 1, 2 or 3 days in a 24 well plate at 37 °C and 5 % CO_2. After this incubation period, cells were washed with PBS, detached using trypsin and EDTA and stained with propidium iodide at a concentration of 10 µg/mL in PBS for 10 min. For the positive control, cells were heat shock-treated for 15 min at 60 °C. Staining has been evaluated with FACS LSRII.

Radiolabeling of macromolecules

The radiolabeling was performed according to Ling et al.[1] *Moraxella catarrhalis* were grown for 15 h on solid BHI medium (Bacto™ Brain Heart Infusion media from BD with addition of 20 µg/ml hemin and 20 µg/ml NAD). The bacteria were harvested with a cotton swap, transferred into fresh liquid BHI media, where the OD_{550} was measured to determine bacteria number. Bacteria suspension was diluted in BHI medium to reach a final bacteria density of $2 \cdot 10^8$ cells/mL in 0.5 mL total volume. Antibiotics with known bactericidal effect (moxifloxacin for DNA synthesis inhibition at 4 µg/mL (4xMIC)[2], rifampicin for inhibition of RNA synthesis at 0.06 µg/mL (2xMIC)[3] or erythromycin for protein synthesis disruption at 2 µg/mL (4xMIC)[4]) as well as compound **8** at 3 µM (6xMIC) were added to the respective suspension. DMSO was used as solvent control. After incubation for 5 min (37°C, shaking at 750 rpm), 2.5 µL of a 1 mCi/mL aqueous solution of either tritium labeled ^{3}H-thymidine (DNA), ^{3}H-uridine (RNA) or ^{3}H-leucine (protein) was added in the respective sample (end concentration: 5 µCi/mL). The sample was incubated for 40 min (37°C, shaking at 750 rpm). Subsequently, 0.5 mL ice-cold 25% trichloroacetic acid in water (w/w) was added, vortexed and centrifuged for 5 min at 13,000 rpm. The supernatant was discarded and the pellet washed with 1 mL ice-cold 25% trichloroacetic acid (2 times) and 1 mL ice-cold water (3 times) with centrifugation for 3 min at 13,000 rpm after each washing step. The washed pellet was suspended in 0.5 mL water by ultra-sonication and mixed with 5 ml scintillation liquid (Ready Safe™ from Beckman) by vortexing for 30 sec. The samples were prepared in biological triplicates and ^{3}H was measured with a scintillation counter (Beckmann LS 6000IC) over 10 min. For determination of monomer incorporation, DPM values of the antibiotic samples were calculated in proportion to the respective controls without antibiotic.

Membrane depolarization assay

The membrane depolarization assay was adjusted for *Moraxella catarrhalis* and performed as described for the BacLight™ Bacterial Membrane Potential Kit (B34950). *Moraxella* spec. were grown as described above and incubated with the amounts of compound **8** as indicated for 5, 15, 30, 45, 60, 90 and 120 minutes respectively. $1 \cdot 10^6$ cells were diluted in 1 mL PBS containing 5 µM CCCP (carbonylcyanide 3-chlorophenylhydrazone; only for CCCP control) and 30 µM $DiOC_2(3)$ (3,3-diethyloxacarbocyanine iodide). Samples have been incubated for 10 min. Fluorescence was measured with BD LSRFortessa. To validate the assay, known cell membrane potential inhibitors have been added in the following concentrations: Amytal 2 mM, Antimycin A 10 µg/mL and KCN at 1 mM. As an additional positive control, bacteria have been heat shocked for 15 min at 60 °C.

Resistance studies

Moraxella catarrhalis were incubated as described above. The bacteria (OD_{550} of 0.2) were grown in 50 mL falcons containing 5 mL medium and compound **8** at 0.1 µM. After 12 hours the same amount of bacteria was transferred into fresh medium containing 0.1 µM compound **8**.

Every day OD_{550} = 0.2 of the challenged bacteria were incubated in 5mL medium containing either 1, 2.5, 10 or 50 µM compound **8** in 1% DMSO. The growth of these cultures was evaluated after 12 hours by measuring the optical density in comparison to control containing DMSO at 1 %.

Scanning electron microscopy

Bacteria were grown on BHI agar and incubated over night at 37 °C and 5 % CO_2. $4 \cdot 10^6$ Bacteria from a 4 h pre-culture (in BHI; 37 °C, 220 rpm) were centrifuged on poly-lysine coated coverslips (3300 g x 15 min.). Compound **8** was added at 0.1, 0.5, 10 and 50 µM and incubated for 3 h at 37 °C and 5 % CO_2. Bacteria were fixed with 2 x 300 µL fixation agent (3 % formaldehyde, 2 % glutardialdehyde, 0.09 M sucrose, 0.01 M $CaCl_2$, 0.01 M $MgCl_2$ in 0.1 M HEPES) for 5 and 25 min respectively. Slips were washed twice with 1 mL 0.1 M HEPES for 5 min each. Dehydration was performed with ethanol-dilutions of 1 mL (30 %, 50 %, 70 % (overnight), 80 %, 90 %, 96 %, 3 x 100 %) for 10 min each, if not indicated otherwise. Afterwards samples were dried with liquid CO_2 in critical point drying changing the CO_2 10 x with 10 min incubation in between, using a Baltec CPD 030 critical point dryer (Baltec, Liechtenstein). Finally, samples were mounted on stubs with silver coating polish (drying overnight at RT), sputtered with 6 nm platinum using Quorum Q 150R ES (Quorum Technologies Ltd, Laughton, East Sussex, UK) in low pressure argon atmosphere and imaged using a Zeiss FIB-FESEM Auriga 40 TM Crossbeam.

Microscopic imaging of A549 cells

$1 \cdot 10^5$ A549 were seeded into 24 well plate and incubated with the concentrations of compound **8** as indicated in 1 mL DMEM + 10 % FCS and incubated for 1, 2 or 3 days. DMSO was added to all samples (treated and untreated) equalling a total concentration of 1 %. Pictures of the cells were taken every day with a Nikon Eclipse TS100 microscope.

Detection of ROS

Moraxella species were incubated as described above. Bacteria were harvested through centrifugation for 10 min at 4000 rpm at RT and washed in oxidative burst buffer (8 g/L NaCl, 0.2 g/L KCl, 0.62 g/L KH_2PO_4, 1.14 g/L Na_2HPO_4, 1 g/L glucose, 50 mg BSA in ddH_2O; pH 7.2 sterile filtered with 0.2 µM filter). $1 \cdot 10^7$ bacteria/well were seeded in 96 well plates in 50 µL oxidative burst buffer. Compound **8** in DMSO (1% final DMSO concentration), KCN or DMSO were premixed in separate plate in 50 µL oxidative burst buffer containing 100 µM lucigenin (Santa Cruz Biotechnology) at their respective concentrations. KCN was used as a ROS inducer at 1 mM and DMSO as solvent control at 1 %. Shortly before the experiment, samples were transferred to a white 96 well plate. Luminescence was measured using a Thermo Fisher Varioskan Flash spectrophotometer.

References

1. Ling, L. L.; Schneider, T.; Peoples, A. J.; Spoering, A. L.; Engels, I.; Conlon, B. P.; Mueller, A.; Schaberle, T. F.; Hughes, D. E.; Epstein, S.; Jones, M.; Lazarides, L.; Steadman, V. A.; Cohen, D. R.; Felix, C. R.; Fetterman, K. A.; Millett, W. P.; Nitti, A. G.; Zullo, A. M.; Chen, C.; Lewis, K., *Nature* **2015,** *517* (7535), 455-459.
2. MacGowan, A. P.; Bowker, K. E.; Wootton, M.; Holt, H. A., *Clin Microbiol Infect* **1999,** *5* (4), 195-200.
3. Thornsberry, C.; Ogilvie, P. T.; Holley, H. P., Jr.; Sahm, D. F., *Antimicrob Agents Chemother* **1999,** *43* (11), 2612-23.
4. Credito, K. L.; Lin, G.; Pankuch, G. A.; Bajaksouzian, S.; Jacobs, M. R.; Appelbaum, P. C., *Antimicrob Agents Chemother* **2001,** *45* (1), 67-72.

2. Supporting Figures

Table S1. Bacterial strains used in this study.

Species	Strain number	Source	Other stain numbers
Moraxella catarrhalis	ATCC 25238	DSMZ[1]	DSM 9143
Moraxella catarrhalis	ATCC 43617	DSMZ[1]	DSM 11994
Moraxella catarrhalis	251	N. Töpfner[2]	16418
Moraxella catarrhalis	252	N. Töpfner[2]	16745
Moraxella catarrhalis	253	N. Töpfner[2]	17833
Moraxella catarrhalis	254	N. Töpfner[2]	20972
Moraxella catarrhalis	255	N. Töpfner[2]	14665
Moraxella catarrhalis	256	N. Töpfner[2]	14668
Moraxella nonliquefaciens	DSM 6327	DSMZ[1]	
Moraxella osloensis	DSM 6998	DSMZ[1]	
Moraxella urethralis	DSM 7531	DSMZ[1]	
Neisseria meningitidis	SerC C1938	T. F. Meyer[3]	N95
Neisseria gonorrhoeae	MS11[a]	T. F. Meyer[3]	
Escherichia coli EPEC	026	T. Oelschläger[6]	
Enterococcus faecalis	438	Eye isolate	
Haemophilus influenzae	NTHi KN2[b]	M. Huff-Nagel[4]	
Haemophilus influenzae	Rd KW20	A. Wright[5]	
Klebsiella pneumoniae	3091	T. Oelschläger[6]	
Mannheimia haemolytica	472	DSMZ[1]	DSM 10531
Pseudomonas aeruginosa	PAO1	DSMZ[1]	DSM 22644
Streptococcus pyogenes	140	S. Backert[7]	
Yersinia pseudotuberculosis	025	T. Oelschläger[6]	

[1]Leibniz-Institute DSMZ-German Collection of Microorganisms and Cell Cultures
[2]Klinik und Poliklinik für Kinder- und Jugendmedizin im Universitätsklinikum Dresden, Germany
[3]Max-Planck-Institute for Infection Biology, Berlin, Germany
[4]Praxis Dr. Huff-Nagel, practicing pediatrician, Konstanz, Germany
[5]Tufts University School of Medicine, Molecular Biology and Microbiology, Boston, USA
[6]Institute for Molecular Infection Biology, Julius-Maximilian-Universität Würzburg, Germany
[7]Friedrich-Alexander Universität Erlangen-Nürnberg, Erlangen, Germany
[a]Edwards, M., et al. (1984) Antigenic analysis of gonococcal pili using monoclonal antibodies. *J Exp Med* 160(6): 1782-91.
[b]Tchoupa, A.K., et al. (2015) Outer membrane protein P1 is the CEACAM-binding adhesin of Haemophilus influenza. *Mol Microbiol* 98(3): 440-55.

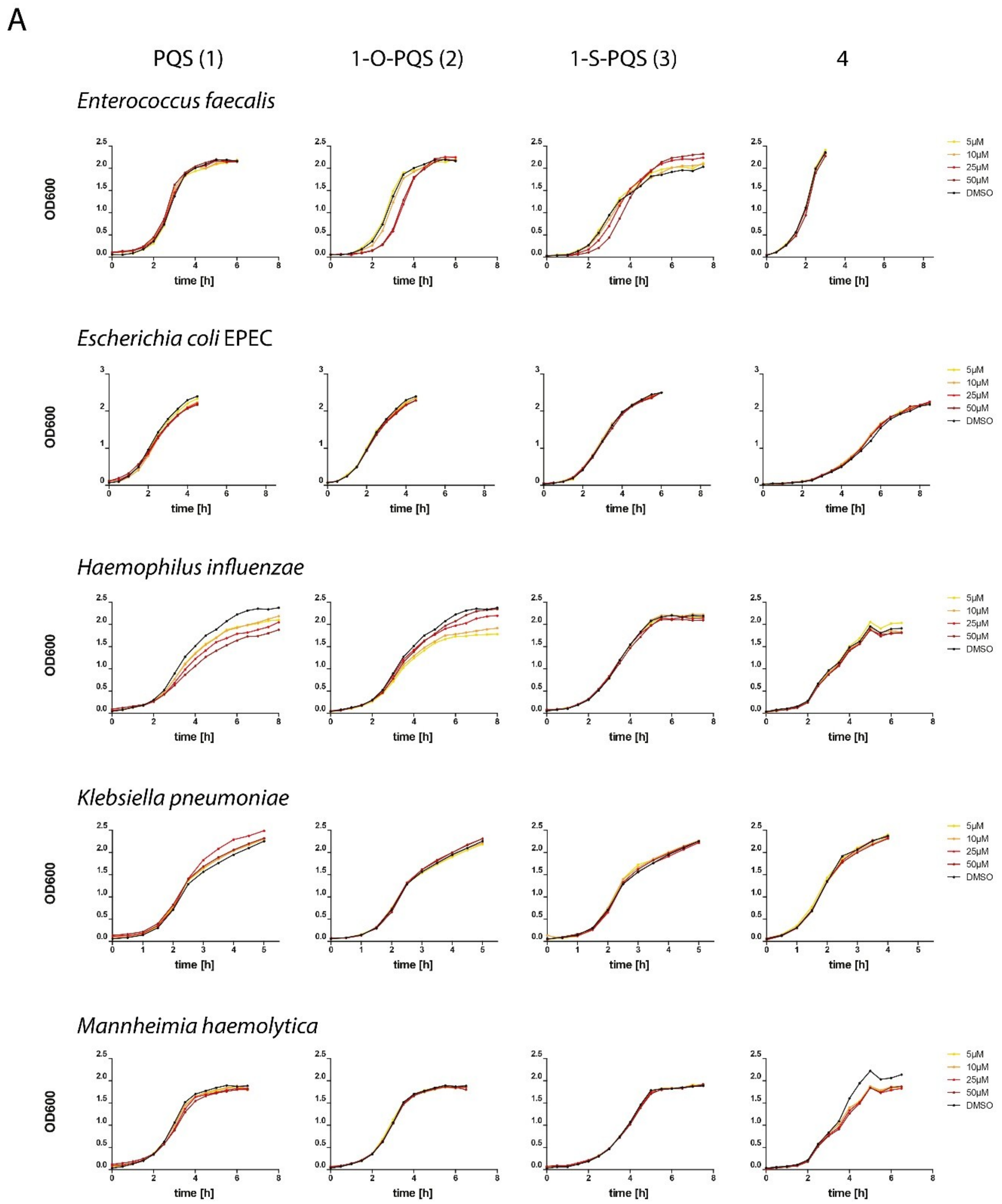
A
PQS (1)
1-O-PQS (2)
1-S-PQS (3)
4
Enterococcus faecalis
Escherichia coli EPEC
Haemophilus influenzae
Klebsiella pneumoniae
Mannheimia haemolytica
OD600
time [h]
5µM
10µM
25µM
50µM
DMSO

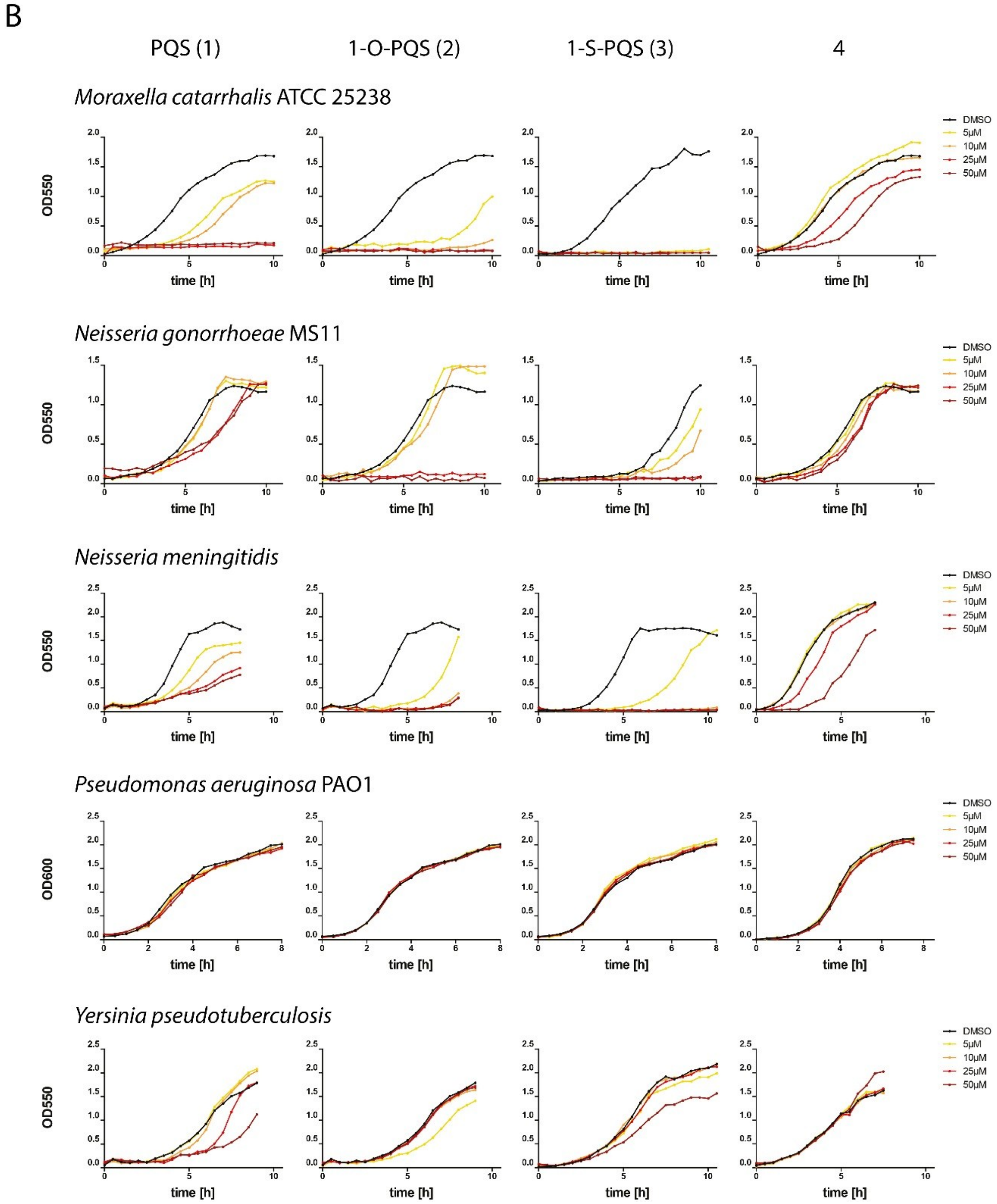

Figure S1. Growth curves of Gram-negative bacteria with the natural product PQS (**1**) and its synthetic derivatives 1-O-PQS (**2**), 1-S-PQS (**3**) and **4**.

A

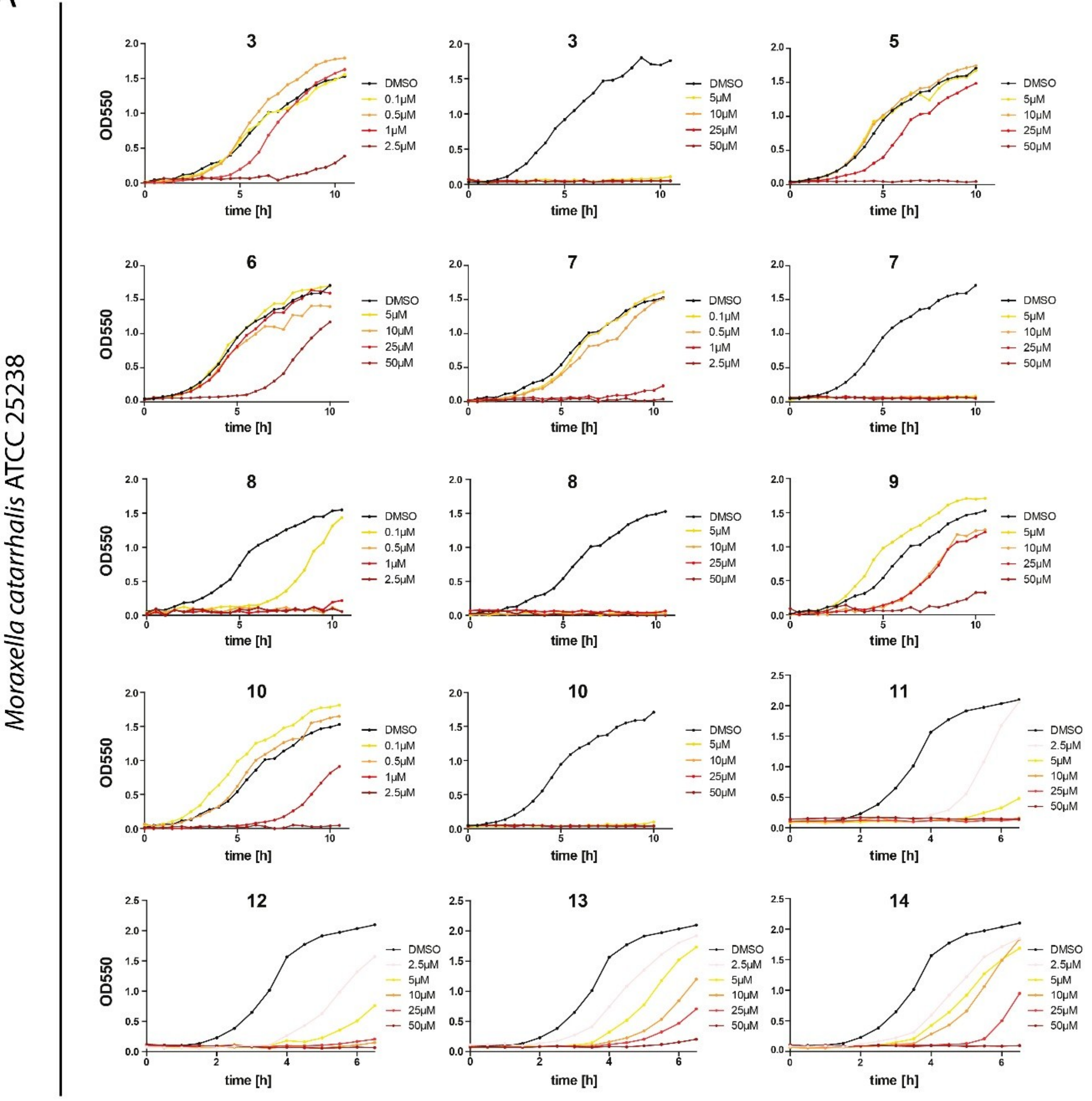

B

strain	compound	0.1µM	0.5µM	1µM	2.5µM	5µM	10µM	25µM	50µM
Moraxella catarrhalis	**3**	0.96	1.16	0.83	0.13	0.02	0.01	0.00	0.00
ATCC 25238	**4**					1.05	0.94	0.67	0.42
	**5**					1.03	1.08	0.71	0.03
	**6**					1.06	0.85	0.96	0.33
	**7**	0.96	0.82	0.07	0.02	0.04	0.02	0.01	0.00
	**8**	0.42	0.09	0.09	0.06	0.03	0.01	0.00	0.01
	**9**					1.30	0.57	0.53	0.05
	**10**	1.30	1.01	0.26	0.02	0.03	0.00	0.01	0.00
	**11**				0.37	0.07	0.02	0.01	0.01
	**12**				0.34	0.11	0.01	0.01	0.00
	**13**				0.63	0.41	0.19	0.10	0.02
	**14**				0.58	0.46	0.39	0.09	0.00

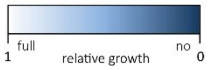

Figure S2. (A) Growth curves of *Moraxella catarrhalis* ATCC 25238 with 1-S-PQS (**3**) and its derivatives **5**-**14**. **(B)** Summarized results of growth curve from S2A. Values ranging from 0 to 1 were obtained as described for growth of bacterial strains.

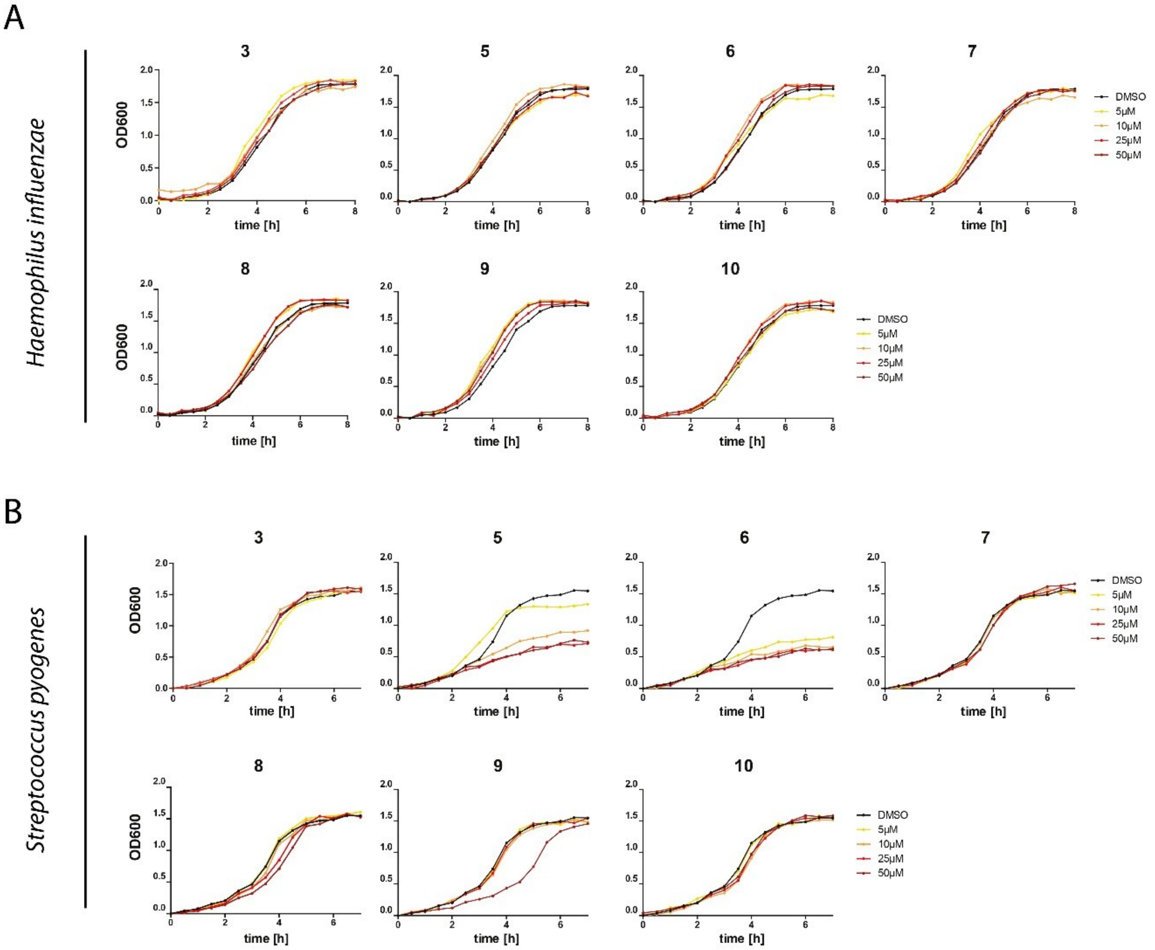

C

Strain	compound	5µM	10µM	25µM	50µM
Haemophilus influenzae non-typeable	**3**	1.14	0.91	1.05	0.99
	**4**	1.04	0.98	0.96	0.94
	**5**	0.96	1.10	0.98	1.05
	**6**	1.01	1.14	1.12	1.04
	**7**	1.08	0.96	1.03	1.00
	**8**	1.09	0.99	1.06	0.95
	**9**	1.18	1.17	1.10	1.14
	**10**	0.98	1.09	1.07	0.99
Streptococcus pyogenes	**3**	0.95	1.05	1.03	1.03
	**5**	0.98	0.63	0.49	0.49
	**6**	0.58	0.49	0.45	0.46
	**7**	0.95	0.99	0.97	1.00
	**8**	1.03	0.97	0.94	0.87
	**9**	0.99	0.96	0.98	0.67
	**10**	1.00	0.93	0.95	0.93

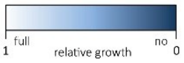

Figure S3. Growth curves of *Haemophilus influenzae* NTHi KN2 **(A)** and *Streptococcus pyogenes* **(B)** with 1-S-PQS (**3**) and its derivatives **5**-**10**. **(C)** Summarized results of growth curve from S3A and S3B. Values from 0 (no growth) to 1 (full growth) were referenced to the controls as described in the section for growth of bacterial strains.

A

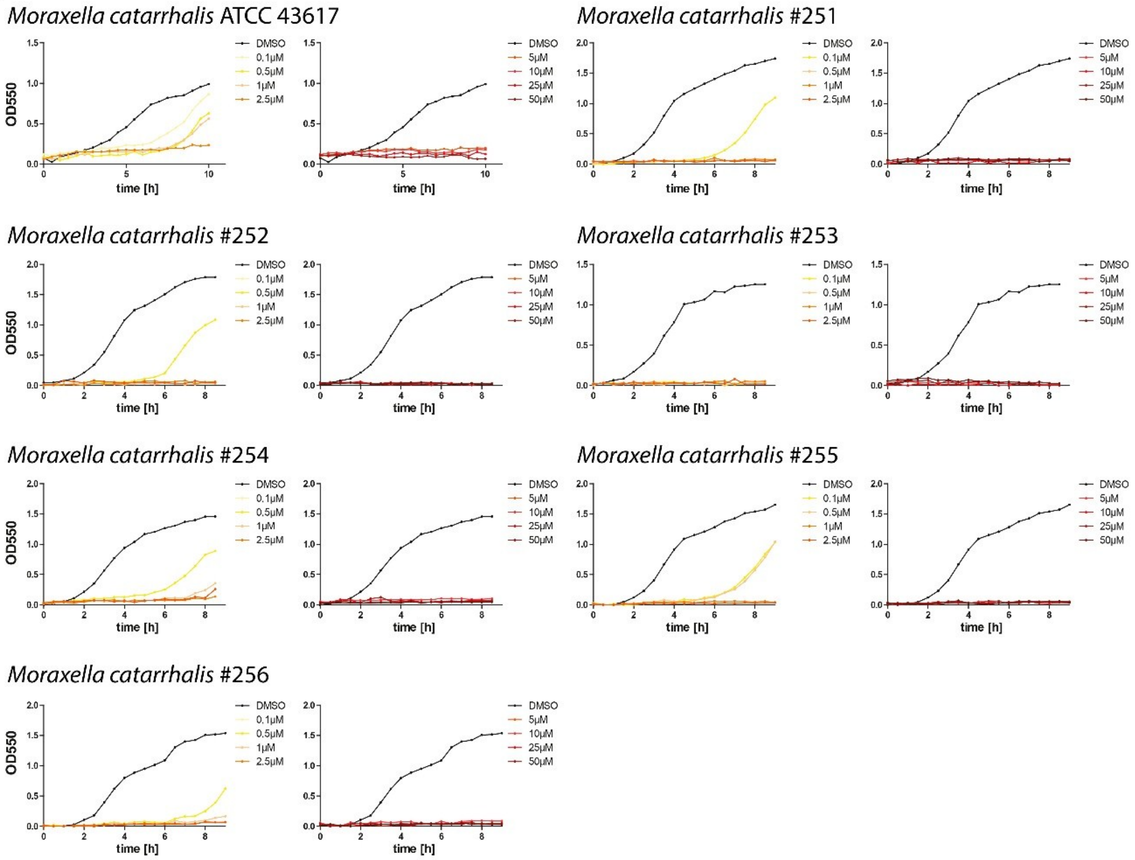

B

		8							
	strain #	0.1μM	0.5μM	1μM	2.5μM	5μM	10μM	25μM	50μM
Moraxella catarrhalis	ATCC 43617	0.44	0.23	0.29	0.26	0.17	0.09	0.02	0.00
clinical isolates	251	0.24	0.03	0.01	0.01	0.03	0.03	0.02	0.00
	252	0.28	0.02	0.04	0.03	0.01	0.00	0.01	0.02
	253	0.01	0.03	0.03	0.01	0.01	0.01	0.03	0.01
	254	0.29	0.11	0.07	0.07	0.04	0.02	0.04	0.01
	255	0.22	0.19	0.02	0.02	0.01	0.01	0.03	0.01
	256	0.14	0.06	0.02	0.04	0.02	0.00	0.00	0.02

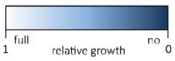

Figure S4. (A) Growth curves of *Moraxella catarrhalis* clinical isolates with compound **8**. **(B)** Summarized results of growth curve from S5A. Values from 0 (no growth) to 1 (full growth) were referenced to the controls as described in the section for growth of bacterial strains.

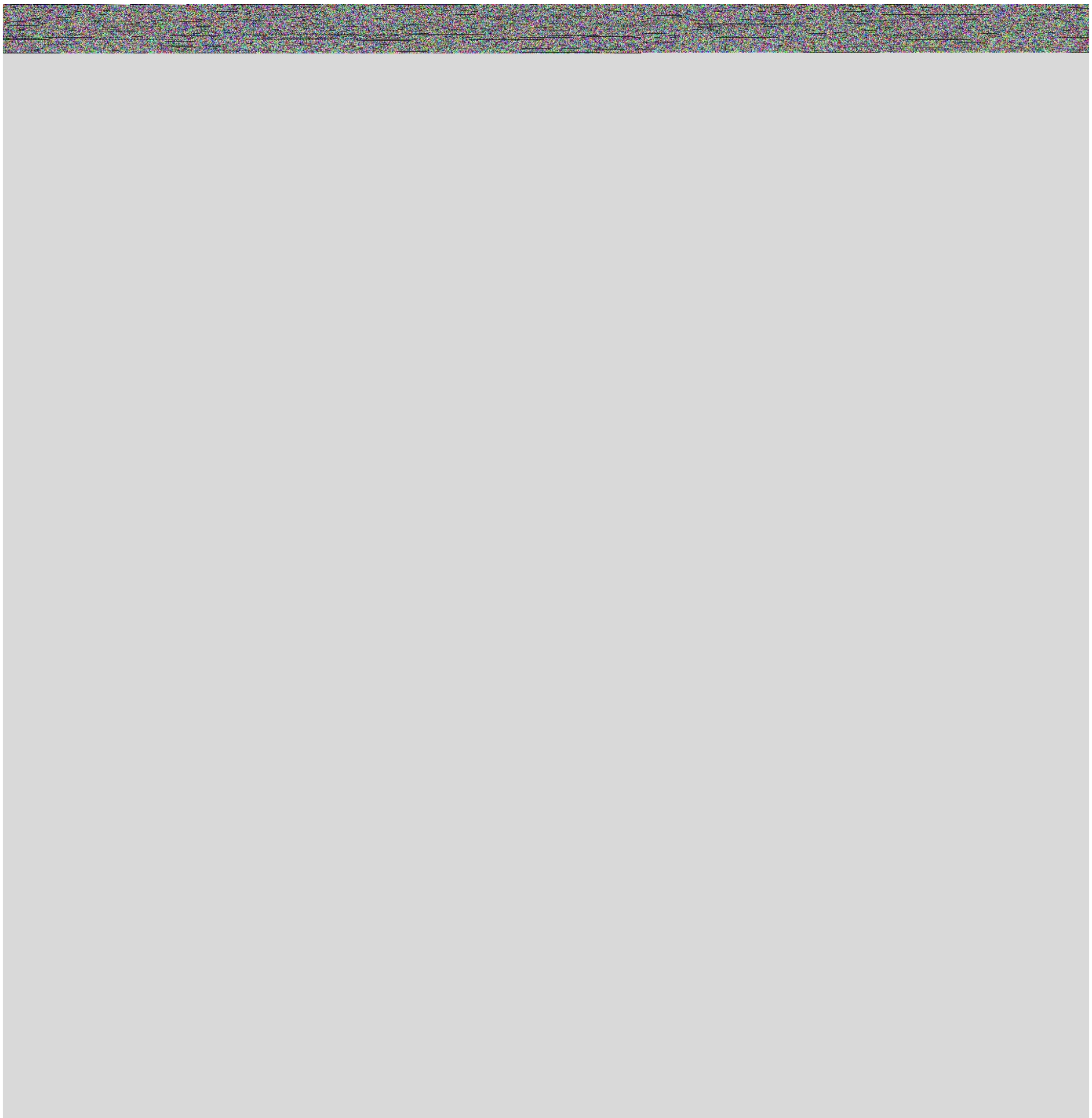

Figure S5. (A) Growth curves of commensal *Moraxella osloensis*, *Moraxella nonliquefaciens* and *Moraxella urethralis* with compound **8**. **(B)** Summarized results of growth curve from S4A. Values from 0 (no growth) to 1 (full growth) were referenced to the controls as described in the section for growth of bacterial strains.

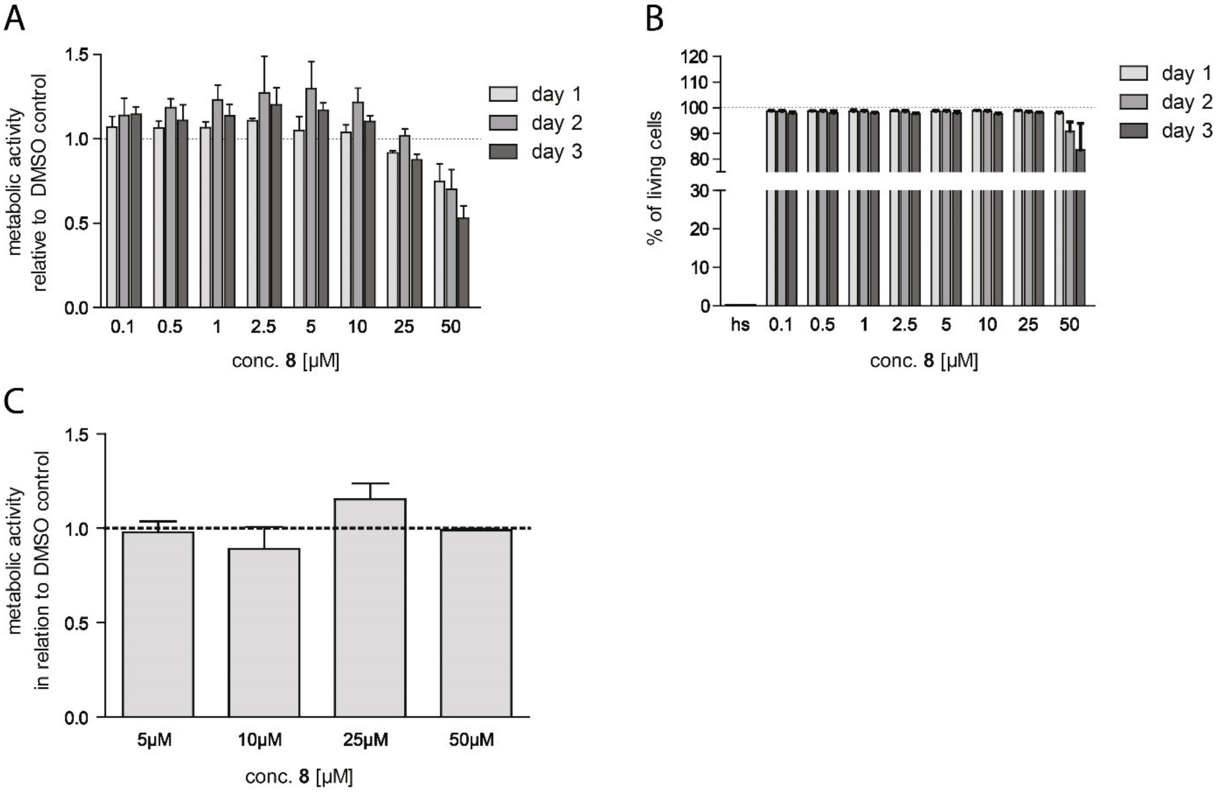

Figure S6. (A) MTT assay of A549 cells with compound **8** over 3 days. **(B)** PI assay of A549 cells with compound **8** over 3 days. **(C)** MTT assay of HEK cells with compound **8** after 1 day.

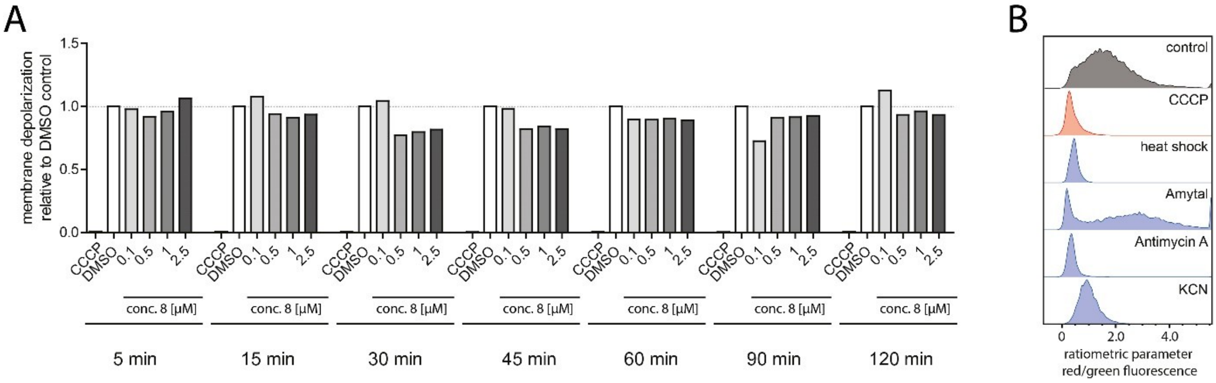

Figure S7. (A) Time resolved changes in membrane polarization of *Moraxella catarrhalis* ATCC 25238 by treatment with different concentrations of compound **8**. DMSO and CCCP were used as negative and positive controls, respectively. Membrane polarization of control is indicated with a dotted line. **(B)** Control experiments for validation of membrane polarization using Amytal (2 mM), Antimycin A (10 µg/mL) and KCN (1 mM). As an additional positive control, bacteria were heat shock-treated for 15 min at 60 °C.

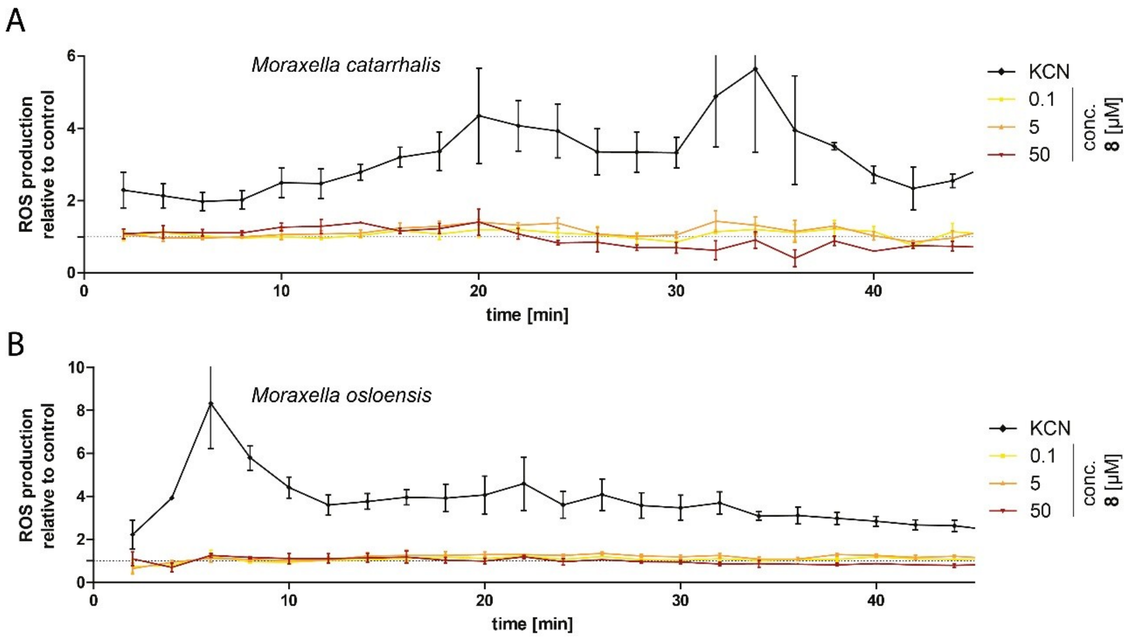

Figure S8. Production of ROS upon treatment of **(A)** *Moraxella catarrhalis* strain ATCC 25238, or **(B)** *Moraxella osloensis* with 0.1, 5 or 50 µM of compound **8**. Levels are depicted in relation to untreated bacteria, which are indicated with a dotted line. KCN served as a ROS inducing positive control. Detection of free radicals was performed using a lucigenin based luminescence reaction.

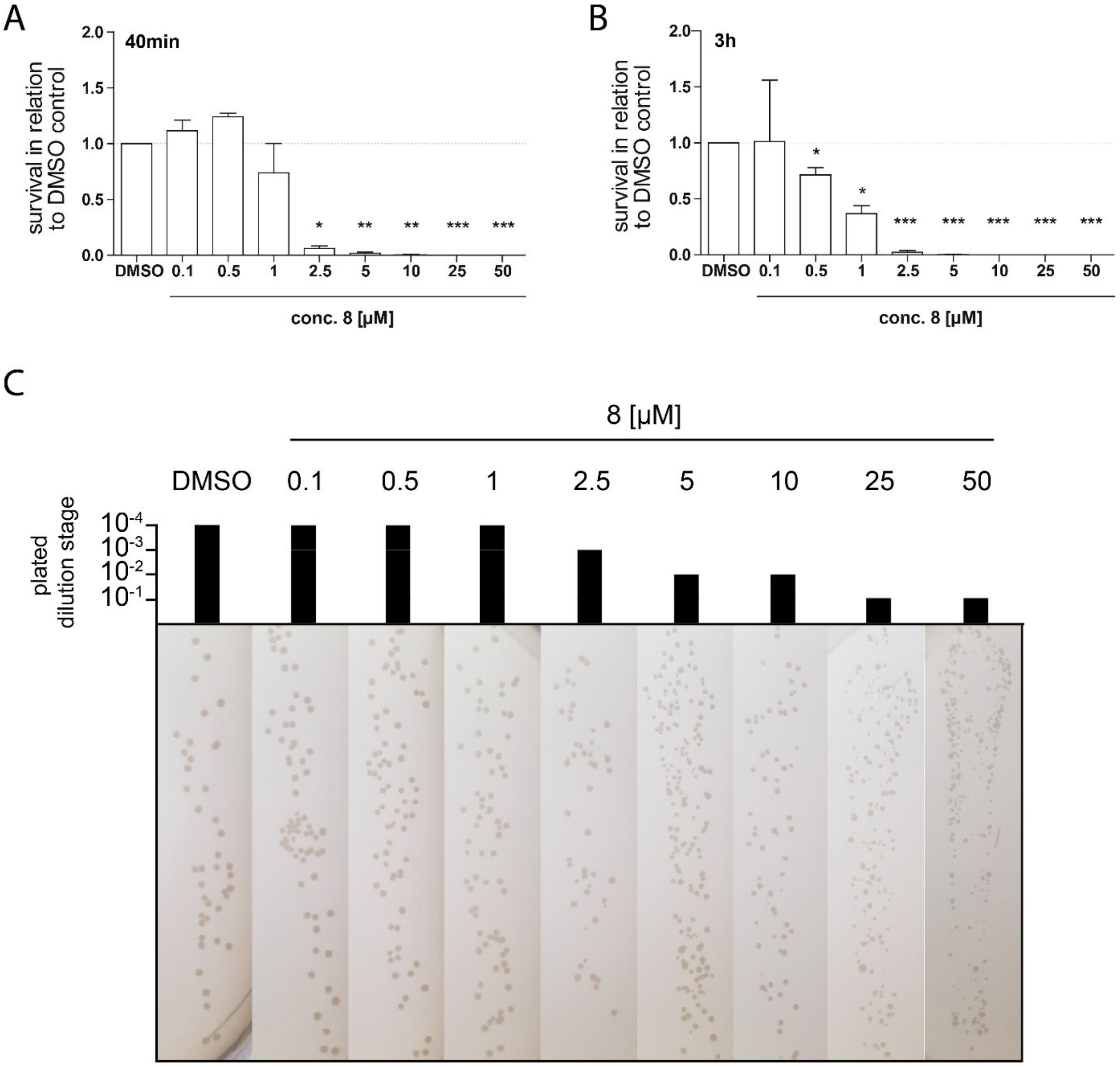

Figure S9. Survival of *Moraxella catarrhalis* ATCC 25238 when treated with different concentrations of compound **8** for A) 40 min and B) 3 hours. C) For manual colony counting, bacteria incubated with 1% DMSO or different concentrations of compound **8** were appropriately diluted as indicated and streaked out in a line on a GC plate. Incubation took place as described in the methods section.

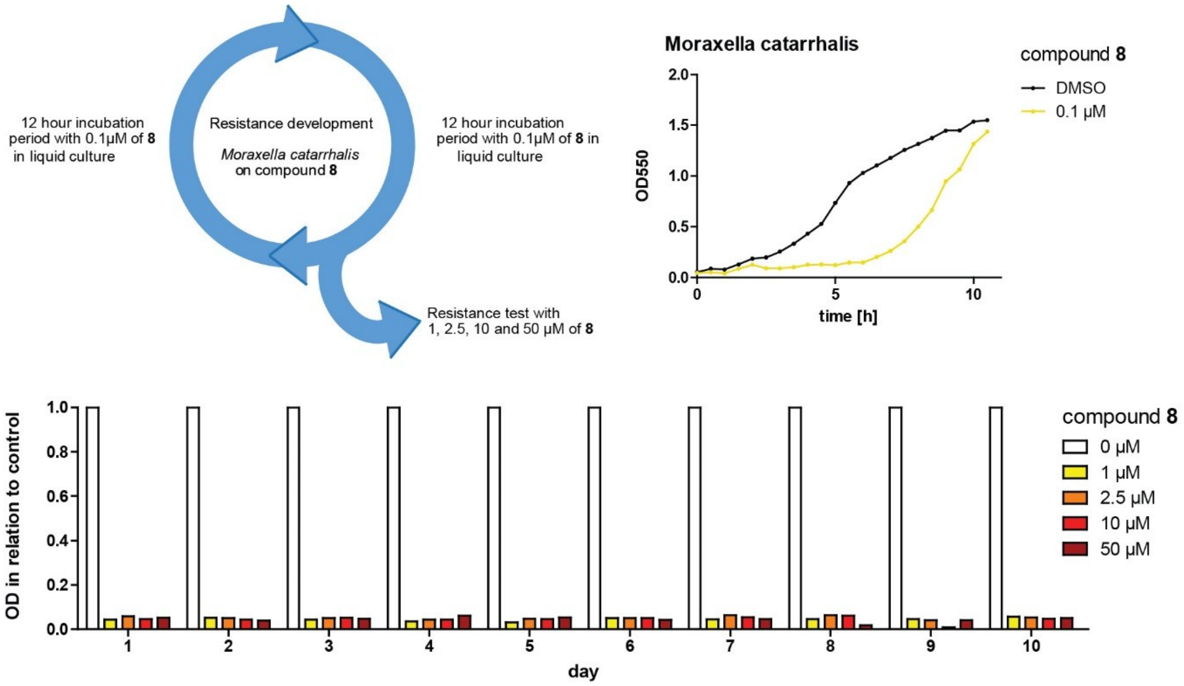

Figure S10. Resistance studies of *Moraxella catarrhalis* ATCC 25238 against different concentrations of compound **8** over 10 days.

3. Syntheses

Chemicals and solvents for the synthesis were purchased from Sigma-Aldrich, Acros Organics, Carl Roth or VWR Chemicals and were used without further purification. For silica gel chromatography, distilled technical grade solvents and silica gel 60 A (Carl Roth) was used. Thin layer chromatography (TLC) was performed using aluminum sheets "TLC silica gel 60 F254" from Merck Millipore® and analyzed with UV-light or by permanganate staining. NMR spectra were obtained with Bruker Avance-III 400, Bruker Avance-III 600 NMR and Bruker Avance Neo 800 spectrometers at ambient temperature. Multiplicities are given as follows: s -singlet, d - doublet, t - triplet, q - quartet, quint. - quintet, m - multiplet. Chemical shifts (δ) are given in parts per million (ppm) relative to the solvent residual signal with $CDCl_3$ δ_H = 7.26 ppm and δ_C = 77.16 ppm, DMSO-d_6 δ_H = 2.50 ppm and δ_C = 39.52 ppm.[1] The data obtained were processed and analyzed with Bruker Topspin 3.5 software. Mass spectrometry data were obtained on an ESI-Orbitrap (Thermo Scientific, LTQ Orbitrap Velos) by direct injection and analyzed with Xcalibur (Thermo Scientific) software. Preparative HPLC was performed on a Spot Prep II system (Armen Instruments).

1. Synthesis of 2-heptyl-3-hydroxychromen-4-one (**2**)

2-heptylchromen-4-one (**2a**)

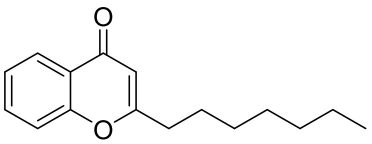

A mixture of 2 g 2´-hydroxyacetophenone (14.7 mmol) and 3.3 g t-BuOK (29.4 mmol, 2 eq.) was dissolved in 50 ml THF and 2.51 ml octanoyl chloride (14.7 mmol) was added. The mixture was stirred at 80 °C for 4h. The reaction was allowed to cool to room temperature and 10 ml conc. H_2SO_4 was added dropwise while the reaction was cooled in a water bath. The mixture was heated to 90 °C and kept at this temperature for 90 min. The reaction was allowed to cool to room temperature and was poured on ice. The water phase was extracted with ether (3x 100 ml) and the combined organic phases washed once with water and dried with $MgSO_4$. After filtration, the solvent was evaporated and the residue purified by column chromatography on silica 60 using ethyl acetate/ petrol ether 9:1. The product was obtained as yellow oil (2.164 g, 60.2 %). ^{1}H-NMR ($CDCl_3$ 400.13 MHz) δ (ppm): 0.88 (m, 3H, H-15), 1.20-1.45 (m, 8H, H-11, H-12, H-13, H-14), 1.73 (m, 2H, H-10), 2.61 (t, 2H, *J* = 7.6 Hz, H-9), 6.17 (s, 1H, H-3), 7.36 (m, 1H, H-6), 7.41 (d, 1H, *J* = 8.4 Hz, H-8), 7.63 (m, 1H, H-7), 8.17 (m, 1H, H-5). ^{13}C-NMR ($CDCl_3$ 100.26 MHz) δ (ppm): 14.2 (C-15), 22.7, 29.1, 29.1, 31.8 (C-11-14, 26.9 (C-10), 34.5 (C-9), 109.9 (C-3), 118.0 (C-8), 123.9 (C-4a), 125.0 (C-6), 133.5 (C-7), 125.8 (C-5), 156.7 (C-8a), 170.0 (C-2) 178.6 (C-4).

2-heptyl-3-hydroxychromen-4-one (**2**)

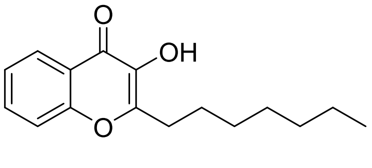

2-heptylchromen-4-one (**2a**) (860 mg, 3.52 mmol) was dissolved in 50 ml toluene and 1.2 ml t-BuOOH and 1.2 ml Triton B were added at 0°C. After stirring at 0°C for 35 min, additional 1.2 ml t-BuOOH and 1.2 ml Triton B were added and the reaction was stirred for another 30 min. The reaction was diluted with ethyl acetate and washed with water and brine, dried with $MgSO_4$, filtered and the solvent evaporated. NMR of the crude residue showed epoxide signals. The crude residue was dissolved in 30 ml DCM and 810 mg TsOH· H_2O was added. The mixture was stirred at room temperature for 2 h. TLC showed almost complete conversion of the epoxide to the blue fluorescing product. The solvent was evaporated and the residue purified by column chromatography on silica 60 using ethyl acetate/ petrol ether 5:1. The product was received as yellow solid (380 mg, 41.5 %). ^{1}H-NMR ($CDCl_3$ 399.79 MHz) δ (ppm): 0.88 (t, *J* = 7.0 Hz, 3H, H-15), 1.21 – 1.45 (m, 8H, H-11, H-12, H-13, H-14), 1.77 (p, *J* = 7.5 Hz, 2H, H-10), 2.84 (t, *J* = 7.7 Hz, 2H, H-9), 6.25 (s, 1H, -O**H**), 7.38 (t, *J* = 7.3 Hz, 1H, H-6), 7.47 (d, *J* = 8.5 Hz, 1H, H-8), 7.64 (t, *J* = 7.7 Hz, 1H, H-7), 8.22 (d, *J* = 7.8 Hz, 1H, H-5). ^{13}C-NMR ($CDCl_3$ 100.54 MHz) δ (ppm): 14.2 (C-15), 22.7 (C-14), 26.8 (C-10), 29.0 (C-9), 29.1 (C-12), 29.3 (C-11), 31.9 (C-13), 118.3 (C-8), 121.4 (C-4a), 124.4 (C-6), 125.6 (C-5), 133.1 (C-7), 138.2 (C-3), 152.5 (C-2), 155.6 (C-8a), 172.6 (C-4).

2. General synthesis of trans-unsaturated acids (**3a**, **7a** and **8a**)

Malonic acid (25 mmol) was dissolved in 10 ml pyridine and the aldehyde (25 mmol, 1 eq.) was added together with 350 µl pyrrolidine (3 mmol, 0.5 mol%). The mixture warmed up and evolution of gas was observed. The mixtures were stirred at room temperature for 20 h and were then poured together into 60 ml 20% H_3PO_4 solution. The mixtures were extracted with ethyl acetate and the combined organic phases washed with brine, dried with $MgSO_4$, filtered and the solvent evaporated. The products were purified by column chromatography using petrol ether/ethyl acetate mixtures.

(*E*)-dec-2-enoic acid (**3a**)

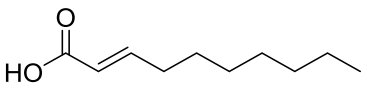

The product was obtained as colorless oil after purification by column chromatography using petrol ether/ethyl acetate 5:1 (73% yield). R_f = 0.175 (PE/EA 5:1). ^{1}H-NMR ($CDCl_3$ 399.79 MHz) δ (ppm): 0.89 (m, 3H, -CH_3), 1.18-1.39 (m, 8H, (-(C**H**$_2$)$_4$-CH_3), 1.47 (m, 2H, =CH-CH_2-C**H**$_2$-), 2.23 (m, 2H, =CH-C**H**$_2$-CH_2-), 5.82 (dt, 1H, *J* = 15.7 Hz, *J* = 1.6 Hz, HOOC-C**H**=CH-), 7.08 (dt, 1H, *J* = 15.7, *J* = 6.9 Hz, HOOC-CH=C**H**-). ^{13}C-NMR ($CDCl_3$ 100.54 MHz) δ (ppm): 14.2 (-CH_3), 28.0 (=CH-CH_2-**C**H$_2$-), 22.8, 29.18, 29.25, 31.9 (-(**C**H$_2$)$_4$-CH_3), 32.5 (=CH-**C**H$_2$-CH_2-), 120.6 (HOOC-**C**H=CH-), 152.6 (HOOC-CH=**C**H-), 171.7 (COOH).

(*E*)-5-phenylpent-2-enoic acid (**7a**)

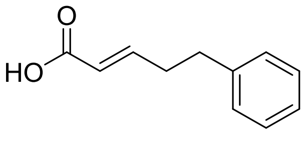

The product was obtained as yellow solid after purification by column chromatography using petrol ether/ethyl acetate 9:1 and 4:1 (43% yield). R_f = 0.2 (PE/EA 4:1). ^{1}H-NMR ($CDCl_3$ 399.79 MHz) δ (ppm): 2.57 (m, 2H, H-4), 2.80 (m, 2H, H-5), 5.85 (dt, 1H, *J* = 15.7, *J* = 1.6 Hz, H-2), 7.11 (dt, 1H, *J* = 15.7, *J* = 7.0 Hz, H-3), 7.19 (m, 2H, H-2´), 7.21 (m, 1H, H-4´), 7.30 (m, 2H, H-3´). ^{13}C-NMR ($CDCl_3$ 100.54 MHz) δ (ppm): 34.1 (C-4), 34.3 (C-5), 121.3 (C-2), 126.4 (C-4´), 128.5 (2C, C-2´), 128.7 (2C, C-3´), 140.7 (C-1´), 151.1 (C-3), 171.3 (C-1).

(*E*)-tridec-2-enoic acid (**8a**)

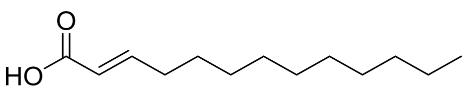

The product was obtained as colorless oil after purification by column chromatography using petrol ether/ethyl acetate 9:1 (61% yield). R_f = 0.2 (PE/EA 5:1). ^{1}H-NMR ($CDCl_3$ 399.79 MHz) δ (ppm): 0.88 (m, 3H, $-CH_3$), 1.23-1.47 (m, 14H, $(-(C\mathbf{H}_2)_7-CH_3)$), 1.47 (m, 2H, $=CH-CH_2-C\mathbf{H}_2-$), 2.23 (m, 2H, $=CH-C\mathbf{H}_2-CH_2-$), 5.82 (dt, 1H, *J* = 15.7 Hz, *J* = 1.7 Hz, HOOC-C**H**=CH-), 7.08 (dt, 1H, *J* = 15.7, *J* = 6.8 Hz, HOOC-CH=C**H**-). ^{13}C-NMR ($CDCl_3$ 100.54 MHz) δ (ppm): 14.2 ($-CH_3$), 28.0 ($=CH-CH_2-\mathbf{C}H_2-$), 22.8, 29.3, 29.45, 29.52, 29.66, 29.7, 32.0 ($-(\mathbf{C}H_2)_7-CH_3$), 32.5 ($=CH-\mathbf{C}H_2-CH_2-$), 120.6 (HOOC-**C**H=CH-), 152.6 (HOOC-CH=**C**H-), 171.4 (COOH).

3. General synthesis of thiochroman-4-ones (**3b**, **7b** and **8b**) from trans-unsaturated acids

The corresponding *trans*-unsaturated acid (1 eq.) was dissolved in dry DCM (1.5 ml for 3 mmol acid) in a Schlenck tube with a septum under nitrogen. Thiophenol (1.eq) was added and the mixture was cooled with an ice-bath to 0°C. Under nitrogen, trifluoromethanesulfonic acid (1 ml for 1 mmol acid) was added and the mixture stirred at 0°C for 5 min. After that, the mixture was heated in a pre-heated oil bath at 130 °C with a reflux cooler for 5 min. The reaction was allowed to cool to room temperature and was poured on ice. DCM was added and the phases separated. The water phase was washed with DCM and the combined organic phases washed twice with sat. $NaHCO_3$ solution and brine. The organic phases was dried with $MgSO_4$, filtered and the solved evaporated. The product was purified by column chromatography on silica 60 using petrol ether/DCM mixtures.

2-heptylthiochroman-4-one (**3b**)

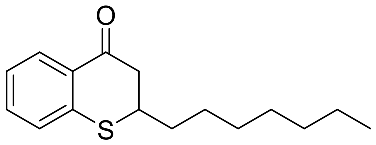

The product was obtained as slightly yellow oil after purification by column chromatography using PE/DCM 4:1 and 1:1 (53% yield). R_f = 0.55 (PE/DCM 1:1). ^{1}H-NMR ($CDCl_3$ 399.79 MHz) δ (ppm): 0.88 (m, 3H, H-15), 1.20-1.37 (m, 8H, H-11-14), 1.46 (m, 2H, H-10), 1.71 (m, 2H, H-9), 2.80 (dd, *J* = 16.2 Hz, *J* = 11.1 Hz, 1H, H-3), 3.04 (dd, *J* = 16.2 Hz, *J* = 3.1 Hz, 1H, H-3), 3.50 (m, 1H, H-2), 7.16 (m, 1H, H-6), 7.27 (m, 1H, H-8), 7.38 (m, 1H, H-7), 8.08 (dd, J = 8.0 Hz, *J* = 1.5 Hz, 1H, H-5). ^{13}C-NMR ($CDCl_3$ 100.53 MHz) δ (ppm): 14.2 (C-15), 22.8, 29.2, 29.3, 31.9 (C-11-14), 26.8 (C-10), 34.7 (C-9), 41.8 (C-2), 46.4 (C-2), 125.0 (C-6), 127.8 (C-8), 129.0 (C-5), 130.8 (C-4a), 133.6 (C-7), 141.9 (C-8a), 194.9 (C-4). ESI-HRMS: m/z = 263.1465 $[M+H]^+$, calc. for $C_{16}H_{22}OS + H^+$ = 263.1464.

2-phenethylthiochroman-4-one (**7b**)

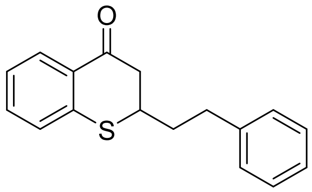

The product was obtained as yellow oil after purification by column chromatography using PE/DCM 1:1 (37% Yield). R_f = 0.5 (PE/DCM 1:1). ^{1}H-NMR ($CDCl_3$ 399.79 MHz) δ (ppm): 1.97 – 2.13 (m, 2H, H-9), 2.73 – 2.89 (m, 3H, H-3a (1H), H-10 (2H)), 3.07 (dd, *J* = 16.3 Hz, 3.2 Hz, 1H, H-3b), 3.43 – 3.51 (m, 1H, H-2), 7.14 – 7.32 (m, 7H, H_{ar}), 7.39 (m, 1H, H-7), 8.08 (dd, *J* = 8.1 Hz, 1.3 Hz, 1H, H-5). ^{13}C-NMR ($CDCl_3$ 100.53 MHz) δ (ppm): 32.9 (C-10), 36.1 (C-9), 40.9 (C-2), 46.3 (C-3), 125.1 (C-6), 126.4 (C-4´), 127.8 (C-8), 128.6 (2C, C-2´), 128.7 (2C, C-3´), 129.1 (C-5), 130.8 (C-4a), 133.7 (C-7), 140.6 (C-1´), 141.4 (C-8a), 194.5 (C-4). ESI-HRMS: m/z = 269.0995 $[M+H]^+$, calc. for $C_{17}H_{16}OS + H^+$ = 269.0995.

2-decylthiochroman-4-one (**8b**)

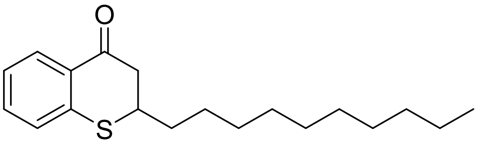

The product was obtained as yellow oil after purification by column chromatography using PE/DCM 4:1 (33% Yield). R_f = 0.1 (PE/DCM 4:1). ^{1}H-NMR ($CDCl_3$ 399.79 MHz) δ (ppm): 0.88 (t, 3H, *J* = 7.0 Hz, H-18), 1.20 – 1.32 (m, 14H, H_{alkyl}), 1.46 (m, 2H, H-10), 1.72 (m, 2H, H-9), 2.80 (dd, *J* = 16.3 Hz, 11.1 Hz, 1H, H-3a), 3.05 (dd, *J* = 16.3 Hz, 3.1 Hz, 1 H, H-3b), 3.50 (m, 1H, H-2), 7.16 (m, 1H, H-6), 7.27 (m, 1H, H-8), 7.38 (m, 1H, H-7), 8.08 (dd, *J* = 8.0 Hz, 1.3 Hz, 1H, H-5). ^{13}C-NMR ($CDCl_3$ 100.53 MHz) δ (ppm): 14.3 (C-18), 22.8 (C-17), 26.8 (C-10), 29.3 (C-11 – C-15), 29.4 (C-11 – C-15), 29.5 (C-11 – C-15), 29.6 (C-11 – C-15), 29.7 (C-11 – C-15), 32.0 (C-16), 34.65 (C-9), 41.8 (C-2), 46.4 (C-3), 125.0 (C-6), 127.8 (C-8), 129.0 (C-5), 130.8 (C-4a), 133.6 (C-7), 141.9 (C-8a), 194.9 (C-4). ESI-HRMS: m/z = 305.1935 $[M+H]^+$, calc. for $C_{19}H_{28}OS + H^+$ = 305.1934.

4. General synthesis of chloropyridinyldecenols (**9a**, **10a** and **11a**)

Chloropyridine was dissolved in dry THF (10 ml for 0.5 g chloropyridine) under nitrogen and the suspension was cooled to -78°C. LDA (2.5 eq.) was slowly added and the reaction stirred at -78°C for 1 h. Afterwards, *trans*-2-decenal (1.2 eq.) was added and the reaction stirred for another 45 min in which the cooling bath temperature was allowed to slowly warm up to -50°C. The reaction was quenched by the addition of sat. NH_4Cl solution. The reaction was extracted with ethyl acetate, the combined organic phases washed with brine, dried with $MgSO_4$, filtered and the solvent evaporated. The products were purified by column chromatography using petrol ether/ethyl acetate mixtures.

(*E*)-1-(2′-chloropyridin-3′-yl)dec-2-en-1-ol (**9a**)

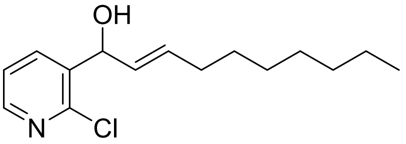

The product was obtained as yellow oil after purification by column chromatography using petrol ether/ethyl acetate 2:1 (42% Yield). R_f = 0.5 (PE/EA 2:1). ^{1}H-NMR ($CDCl_3$ 399.79 MHz) δ (ppm): 0.87 (t, 3H, *J* = 7.0 Hz, H-10), 1.18 – 1.32 (m, 8H, H-6 – H-9), 1.37 (m, 2H, H-5), 2.04 (m, 2H, H-4), 5.50 (m, 1H, H-1), 5.53 (m, 1H, H-2), 5.83 (m, 1H, H-3), 7.27 (dd, 1H, *J* = 7.7 Hz, *J* = 4.8 Hz, H-5′), 7.94 (dd, 1H, *J* = 7.7 Hz, *J* = 2.0 Hz, H-4′), 8.29 (dd, 1H, *J* = 4.8 Hz, *J* = 2.0 Hz, H-6′). ^{13}C-NMR ($CDCl_3$ 100.53 MHz) δ (ppm): 14.2 (C-10), 22.8, 29.1, 29.20 (2C), 31.9, 32.3 (C-4 – C-9), 71.2 (C-1), 123.0 (C-5′), 129.5 (C-2), 134.8 (C-3), 136.5 (C-4′), 137.6 (C-3′), 148.4 (C-6′), 149.4 (C-2′). ESI-HRMS: m/z = 268.1459 $[M+H]^+$, calc. for $C_{15}H_{22}ClNO + H^+$ = 268.1463.

(*E*)-1-(4′-chloropyridin-3′-yl)dec-2-en-1-ol (**10a**)

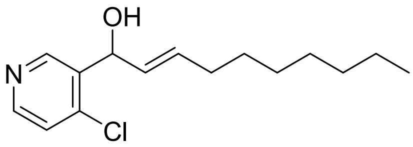

The product was obtained as yellow oil after purification by column chromatography using petrol ether/ethyl acetate 2:1 (86% Yield). R_f = 0.35 (PE/EA 1:1). ^{1}H-NMR ($CDCl_3$ 399.79 MHz) δ (ppm): 0.87 (t, 3H, *J* = 7.0 Hz, H-10), 1.17 – 1.32 (m, 8H, H-6 – H-9), 1.37 (m, 2H, H-5), 2.05 (m, 2H, H-4), 5.56 (m,

1H, H-1), 5.60 (m, 1H, H-2), 5.83 (m, 1H, H-3), 7.31 (d, 1H, *J* = 5.4 Hz, H-5´), 8.41 (d, 1H, *J* = 5.4 Hz, H-6´), 8.78 (s, 1H, H-2´). ^{13}C-NMR ($CDCl_3$ 100.53 MHz) δ (ppm): 14.2 (C-10), 22.8, 29.0, 29.2 (2C), 31.9, 32.2 (C-4 – C-9), 70.6 (C-1), 124.7 (C-5´), 129.5 (C-2), 134.9 (C-3), 137.0 (C-3´), 143.0 (C-4´), 148.7 (C-6´), 148.9 (C-2´). ESI-HRMS: m/z = 268.1458 $[M+H]^+$, calc. for $C_{15}H_{22}ClNO + H^+$ = 268.1463.

(*E*)-1-(3´-chloropyridin-4´-yl)dec-2-en-1-ol (**11a**)

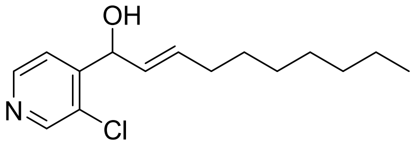

The product was obtained as yellow oil after purification by column chromatography using petrol ether/ethyl acetate 3:1 (50% Yield). R_f = 0.6 (PE/EA 1:1). ^{1}H-NMR ($CDCl_3$ 399.79 MHz) δ (ppm): 0.87 (t, 3H, *J* = 7.0 Hz, H-10), 1.21 – 1.29 (m, 8H, H-6 – H-9), 1.36 (m, 2H, H-5), 2.04 (m, 2H, H-4), 5.50 (m, 1H, H-1), 5.52 (m, 1H, H-2), 5.84 (m, 1H, H-3), 7.55 (d, 1H, *J* = 5.0 Hz, H-5´), 8.49 (d, 1H, *J* = 5.0 Hz, H-6´), 8.51 (s, 1H, H-2´). ^{13}C-NMR ($CDCl_3$ 100.53 MHz) δ (ppm): 14.2 (C-10), 22.8, 29.0, 29.18, 29.20, 31.9, 32.3 (C-4 – C-9), 70.9 (C-1), 121.6 (C-5´), 128.9 (C-2), 135.4 (C-3), 130.0 (C-4´), 148.3 (C-6´), 149.4 (C-2´), 149.5 (C-3´). ESI-HRMS: m/z = 268.1459 $[M+H]^+$, calc. for $C_{15}H_{22}ClNO + H^+$ = 268.1463.

5. General synthesis of chloropyridinyldecenones (**9b**, **10b** and **11b**)

The corresponding alcohol (**9a**, **10a**, and **11a**) was dissolved in acetone (4 ml for 1 mmol alcohol) and MnO_2 (90%, 11 eq.) was added. The mixture was stirred at room temperature for 60 min. The mixture was filtrated and the filtrate evaporated. The residue was purified by column chromatography using petrol ether/ethyl acetate mixtures.

(*E*)-1-(2´-chloropyridin-3´-yl)dec-2-en-1-one (**9b**)

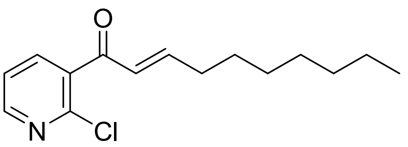

The product was obtained as yellow oil after purification by column chromatography using petrol ether/ethyl acetate 5:1 (45% Yield). R_f = 0.6 (PE/EA 5:1). ^{1}H-NMR ($CDCl_3$ 399.79 MHz) δ (ppm): 0.88 (t, 3H, *J* = 7.0 Hz, H-10), 1.16 – 1.35 (m, 8H, H-6 – H-9), 1.47 (m, 2H, H-5), 2.28 (m, 2H, H-4), 6.49 (dt, 1H, *J* = 15.8 Hz, *J* = 1.5 Hz, H-2), 6.75 (dt, 1H, *J* = 15.8 Hz, *J* = 6.9 Hz, H-3), 7.32 (dd, 1H, J = 7.4 Hz, *J* = 4.9 Hz, H-5´), 7.71 (dd, 1H, *J* = 7.4 Hz, *J* = 2.0 Hz, H-4´), 8.48 (dd, 1H, *J* = 4.9 Hz, *J* = 2.0, H-6´). ^{13}C-NMR ($CDCl_3$ 100.53 MHz) δ (ppm): 14.2 (C-10), 22.7, 29.1, 29.3, 31.8 (C-6 – C-9), 28.0 (C-5), 33.0 (C-4), 122.4 (C-5´), 129.9 (C-2), 135.6 (C-3´), 138.2 (C-4´), 147.8 (C-2´), 150.9 (C-6´), 153.8 (C-3), 192.5 (C-1). ESI-HRMS: m/z = 266.1306 $[M+H]^+$, calc. for $C_{15}H_{20}ClNO + H^+$ = 266.1306.

(*E*)-1-(4´-chloropyridin-3´-yl)dec-2-en-1-one (**10b**)

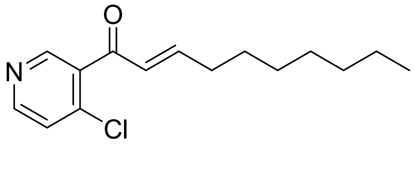

The product was obtained as yellow oil after purification by column chromatography using petrol ether/ethyl acetate 2:1 (63% Yield). R_f = 0.3 (PE/EA 5:1). ^{1}H-NMR ($CDCl_3$ 600.3 MHz) δ (ppm): 0.88 (t, 3H, *J* = 7.0 Hz, H-10), 1.26 – 1.34 (m, 8H, H-6 – H-9), 1.49 (m, 2H, H-5), 2.31 (m, 2H, H-4), 6.50 (dd, 1H, *J* = 15.9 Hz, J = 1.3 Hz, H-2), 6.78 (dt, 1H, *J* = 15.9 Hz, *J* = 6.8 Hz, H-3), 7.50 (d, 1H, *J* = 5.2 Hz, H-5´), 8.61

(s, br, 2H, H-6´ and H-2´). ^{13}C-NMR ($CDCl_3$ 100.53 MHz) δ (ppm): 14.2 (C-10), 22.7, 29.1, 29.3, 31.8 (C-6 – C-9), 28.0 (C-5), 33.0 (C-4), 125.5 (C-5´), 130.1 (C-2), 135.2 (C-3´), 142.4 (C-4´), 149.1 (C-2´), 150.9 (C-6´), 154.4 (C-3), 191.4 (C-1). ESI-HRMS: m/z = 266.1307 $[M+H]^+$, calc. for $C_{15}H_{20}ClNO + H^+$ = 266.1306.

(*E*)-1-(3´-chloropyridin-4´-yl)dec-2-en-1-one (**11b**)

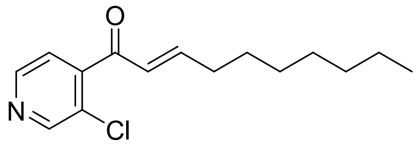

The product was obtained as yellow oil after purification by column chromatography using petrol ether/ethyl acetate 9:1 (50% Yield). R_f = 0.4 (PE/EA 7:1). ^{1}H-NMR ($CDCl_3$ 399.79 MHz) δ (ppm): 0.87 (t, 3H, *J* = 7.0 Hz, H-10), 1.19 – 1.34 (m, 8H, H-6 – H-9), 1.46 (m, 2H, H-5), 2.28 (m, 2H, H-4), 6.41 (dt, 1H, *J* = 15.9 Hz, *J* = 1.5 Hz, H-2), 6.68 (dt, 1H, *J* = 15.9 Hz, *J* = 6.8 Hz, H-3), 7.22 (d, 1H, *J* = 4.8 Hz, H-5´), 8.56 (d, 1H, *J* = 4.8 Hz, H-6´), 8.65 (s, 1H, H-2´). ^{13}C-NMR ($CDCl_3$ 100.53 MHz) δ (ppm): 14.2 (C-10), 22.7, 29.1, 29.2, 31.8 (C-6 – C-9), 27.9 (C-5), 33.1 (C-4), 122.4 (C-5´), 128.2 (C-4´), 129.7 (C-2), 145.9 (C-3´), 148.0 (C-6´), 150.4 (C-2´), 155.2 (C-3), 192.4 (C-1). ESI-HRMS: m/z = 266.1302 $[M+H]^+$, calc. for $C_{15}H_{20}ClNO + H^+$ = 266.1306.

6. General synthesis of thiochroman-4-ones (**9c**, **10c** and **11c**)

The corresponding chloropyridinyldecenone (**9b**, **10b**, and **11b**) was dissolved in DMF (3 ml for 0.7 mmol of educt) and the solution cooled to 0°C. NaSH · n H_2O (1.1 eq.) was added and the mixture stirred at 0°C for 2h. The reaction was quenched by the addition of sat. NH_4Cl solution and extracted with ethyl acetate. The combined organic phases were washed with brine, dried with $MgSO_4$, filtered and the solvent evaporated. The residue was purified by column chromatography using petrol ether/ethyl acetate mixtures.

2-heptyl-2,3-dihydro-4*H*-thiopyrano[2,3-*b*]pyridin-4-one (**9c**)

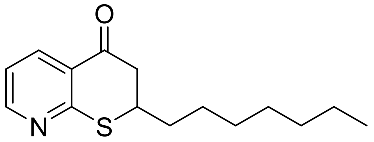

The product was obtained as yellow oil after purification by column chromatography using petrol ether/ethyl acetate 3:1 (35% Yield). R_f = 0.65 (PE/EA 3:1). ^{1}H-NMR ($CDCl_3$ 399.79 MHz) δ (ppm): 0.88 (t, 3H, *J* = 7.0 Hz, H-15), 1.22-1.35 (m, 8H, H-11 – H14), 1.49 (m, 2H, H-10), 1.75 (m, 2H, H-9), 2.84 (dd, *J* = 16.2 Hz, *J* = 10.7 Hz, 1H, H-3a), 3.07 (dd, *J* = 16.2 Hz, *J* = 3.1 Hz, 1H, H-3b), 3.53 (m, 1H, H-2), 7.12 (dd, 1H, *J* = 7.8 Hz, *J* = 4.7 Hz, H-6), 8.27 (dd, 1H, *J* = 7.8 Hz, *J* = 1.9 Hz, H-5), 8.54 (dd, 1H, *J* = 4.7 Hz, *J* = 1.9 Hz, H-7). ^{13}C-NMR ($CDCl_3$ 100.53 MHz) δ (ppm): 14.2 (C-15), 22.7, 29.17, 29.24, 31.8 (C-11-14), 26.8 (C-10), 34.6 (C-9), 40.4 (C-2), 45.3 (C-3), 120.1 (C-6), 127.3 (C-4a), 136.4 (C-5), 153.9 (C-7), 154.7 (C-8a), 194.6 (C-4). ESI-HRMS: m/z = 264.1411 $[M+H]^+$, calc. for $C_{15}H_{21}NOS + H^+$ = 264.1417.

2-heptyl-2,3-dihydro-4*H*-thiopyrano[3,2-c]pyridin-4-one (**10c**)

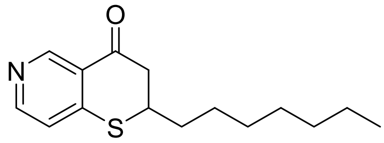

The product was obtained as yellow solid after purification by column chromatography using petrol ether/ethyl acetate 3:1 (20% Yield). R_f = 0.2 (PE/EA 5:1). ^{1}H-NMR ($CDCl_3$ 399.79 MHz) δ (ppm): 0.88 (m,

3H, H-15), 1.22-1.34 (m, 8H, H-11 – H14), 1.46 (m, 2H, H-10), 1.74 (m, 2H, H-9), 2.82 (dd, *J* = 16.2 Hz, *J* = 10.8 Hz, 1H, H-3a), 3.05 (dd, *J* = 16.2 Hz, *J* = 3.1 Hz, 1H, H-3b), 3.55 (m, 1H, H-2), 7.17 (d, 1H, *J* = 5.6 Hz, H-8), 8.41 (d, 1H, *J* = 5.6 Hz, H-7), 9.09 (s, 1H, H-5). ^{13}C-NMR ($CDCl_3$ 100.53 MHz) δ (ppm): 14.2 (C-15), 22.7, 29.15, 29.2, 31.8 (C-11-14), 26.7 (C-10), 34.6 (C-9), 41.9 (C-2), 45.4 (C-3), 122.1 (C-8), 125.9 (C-4a), 149.6 (C-5), 151.1 (C-6), 153.1 (C-8a), 193.6 (C-4). ESI-HRMS: m/z = 264.1412 $[M+H]^+$, calc. for $C_{15}H_{21}NOS + H^+$ = 264.1417.

2-heptyl-2,3-dihydro-4*H*-thiopyrano[2,3-c]pyridin-4-one (**11c**)

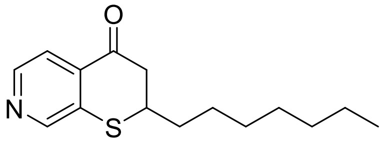

The product was obtained as yellow oil after purification by column chromatography using petrol ether/ethyl acetate 9:1 (15% Yield). R_f = 0.35 (PE/EA 7:1). ^{1}H-NMR ($CDCl_3$ 600.33 MHz) δ (ppm): 0.88 (t, 3H, *J* = 7.0 Hz, H-15), 1.21-1.35 (m, 8H, H-11 – H14), 1.47 (m, 2H, H-10), 1.74 (m, 2H, H-9), 2.84 (dd, *J* = 16.5 Hz, *J* = 11.1 Hz, 1H, H-3a), 3.08 (dd, *J* = 16.5 Hz, *J* = 3.0 Hz, 1H, H-3b), 3.54 (m, 1H, H-2), 7.79 (d, 1H, *J* = 5.2 Hz, H-5), 8.43 (d, 1H, *J* = 5.2 Hz, H-6), 8.62 (s, 1H, H-8). ^{13}C-NMR ($CDCl_3$ 100.53 MHz) δ (ppm): 14.2 (C-15), 22.7, 29.17, 29.22, 31.8 (C-11-14), 26.8 (C-10), 34.5 (C-9), 42.0 (C-2), 46.2 (C-3), 120.6 (C-5), 135.4 (C-4a), 137.1 (C-8a), 146.1 (C-6), 149.9 (C-8), 194.3 (C-4). ESI-HRMS: m/z = 264.1413 $[M+H]^+$, calc. for $C_{15}H_{21}NOS + H^+$ = 264.1417.

7. General synthesis of 3-hydroxy-thiochromen-4-ones (**3** and **7**) from thiochroman-4-ones **3b** and **7b**)

The respective thiochroman-4-one was dissolved in a mixture of MeOH and EtOH (1:1) (10 ml for 1.25 mmol thiochromanone) under a nitrogen atmosphere and isoamyl nitrite (3 eq.) was added. At 0°C, conc. HCl sol. (0.75 ml for 1.25 mmol thiochromanone) was added and the mixture stirred at 0°C for 10 min and 1h at room temperature until the reaction was kept under reflux conditions for 3.5 h. After the mixture was cooled to room temperature and quenched by the addition of water, the mixture was extracted with ether and the combined organic phases were washed with brine, dried with $MgSO_4$, filtered and the solvent was evaporated. The residue was purified by column chromatography using petrol ether/ethyl acetate mixtures.

2-heptyl-3-hydroxy-4*H*-thiochromen-4-one (**3**)

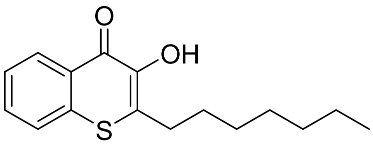

The product was obtained as brown solid after purification by column chromatography using PE/EA 4:1 (20% yield). R_f = 0.65 (PE/EA 4:1). ^{1}H-NMR ($CDCl_3$ 400.1 MHz) δ (ppm): 0.96 (m, 3H, H-15), 1.22 – 1.32 (m, 4H, H-13 and H-14), 1.35 (m, 2H, H-12), 1.41 (m, 2H, H-11), 1.75 (m, 2H, H-10), 2.84 (m, 2H, H-9), 7.51 (m, 1H, H-6), 7.58 (m, 1H, H-7), 7.63 (m, 1H, H-8), 8.53 (d, J = 8.0 Hz, 1H, H-5). ^{13}C-NMR ($CDCl_3$ 100.6 MHz) δ (ppm): 14.2 (C-15), 22.8, 29.07, 29.12, 29.3, 31.8 (4C, C-10 – C-14), 31.3 (C-9), 126.7 (C-6), 128.9 (C-5), 129.0 (C-4a), 129.2 (C-2), 130.8 (C-7), 138.0 (C-8a), 144.1 (C-3), 174.1 (C-4). ESI-HRMS: m/z = 277.1256 $[M+H]^+$, calc. for $C_{16}H_{20}O_2S + H^+$ = 277.1257.

3-hydroxy-2-phenethyl-4*H*-thiochromen-4-one (**7**)

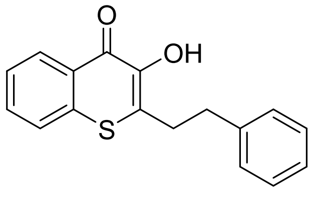

The product was obtained as red/brown solid after purification by column chromatography using PE/EA 5:1 (20% Yield). R_f = 0.6 (PE/EA 5:1). ^{1}H-NMR ($CDCl_3$ 600.3 MHz) δ (ppm): 3.04 – 3.15 (m, 4H, H-9/H-10), 7.22 (m, 1H, H-4´), 7.24 – 7.32 (m, 4H, H-2' and H-3'), 7.53 (ddd, 1H, J = 8.2 Hz, J = 7.5 Hz, J = 1.4 Hz, H-6), 7.59 (ddd, 1H, J = 8.3 Hz, J = 7.5 Hz, J = 1.5 Hz, H-7), 7.62 (dd, 1H, J = 8.3 HZ, J = 1.4 Hz, H-8), 8.54 (dd, J = 8.2 Hz, J = 1.5 Hz, H-5). ^{13}C-NMR ($CDCl_3$ 150.9 MHz) δ (ppm): 33.4 (C-9), 34.9 (C-10), 126.2 (C-8), 126.5 (C-4´), 126.8 (C-6), 127.7 (C-2), 128.6 and 128.7 (4C, C-2´ and C-3´), 128.9 (C-5), 129.0 (C-4a), 131.0 (C-7), 137.9 (C-8a), 140.5 (C-1'), 144.3 (C-3), 174.2 (C-4). ESI-HRMS: m/z = 283.0784 $[M+H]^+$, calc. for $C_{17}H_{14}O_2S + H^+$ = 283.0787.

<u>8. General synthesis of 3-hydroxy-thiochromen-4-ones (**8**, **9** and **10**) from thiochroman-4-ones (**8b**, **9c** and **10c**)</u>

The corresponding thiochroman-4-one was dissolved in EtOH (10 ml for 1.0 mmol thiochromanone) under a nitrogen atmosphere and conc. HCl sol. (0.5 ml for 1.0 mmol thiochromanone) was added. At room temperature, isoamyl nitrite (4.3 ml for 1.0 mmol thiochromanone) was added and the mixture stirred overnight at room temperature. The reaction was quenched by the addition of water, extracted with ethyl acetate and the combined organic phases were washed with brine, dried with $MgSO_4$, filtered and the solvent evaporated. The residue was purified by column chromatography using petrol ether/ethyl acetate mixtures.

2-decyl-3-hydroxy-4*H*-thiochromen-4-one (**8**)

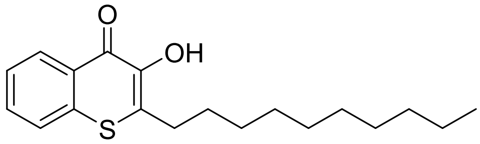

The product was obtained as yellow solid after purification by column chromatography using PE/EA 7:1 (15% Yield). R_f = 0.55 (PE/EA 7:1). ^{1}H-NMR ($CDCl_3$ 399.79 MHz) δ (ppm): ^{1}H-NMR ($CDCl_3$ 399.79 MHz) δ (ppm): 0.88 (m, 3H, H-18), 1.16 – 1.38 (m, 14H, H-11 – H-17), 1.75 (m, 2H, H-10), 2.84 (m, 2H, H-9), 7.52 (m, 1H, H-6), 7.59 (m, 1H, H-7), 7.64 (d, 1H, *J* = 7.7 Hz, H-8), 8.53 (d, 1H, *J* = 8.2 Hz, H-5). ^{13}C-NMR ($CDCl_3$ 100.53 MHz) δ (ppm): 14.2 (C-18), 22.8, 29.37, 29.45 (2C), 29.6, 29.7, 32.0 (C-11-17), 29.1 (C-10), 31.1 (C-9), 126.1 (C-8), 126.7 (C-6), 128.9 (C-5), 129.0 (C-4a), 129.5 (C-2), 130.9 (C-7), 138.0 (C-8a), 144.1 (C-3), 174.1 (C-4). ESI-HRMS: m/z = 319.1722 $[M+H]^+$, calc. for $C_{19}H_{26}O_2S + H^+$ = 319.1726.

2-heptyl-3-hydroxy-4*H*-thiopyrano[2,3-b]pyridin-4-one (**9**)

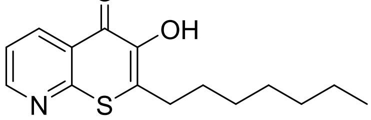

The product was obtained as slightly yellow solid after pre-purification by column chromatography using petrol ether/ethyl acetate 5:1 and eventually purification by prep. HPLC using H_2O + 0.1% FA (A)

and MeCN + 0.1% FA (B) with a linear gradient 5% A→ 95% B in 35 min on a Reprosil-Pur 120 C-18-AQ column (250x20 mm, Dr. Maisch) (25% Yield). R_f = 0.42 (PE/EA 5:1). ^{1}H-NMR ($CDCl_3$ 399.79 MHz) δ (ppm): 0.88 (t, 3H, *J* = 7.0 Hz, H-15), 1.24-1.32 (m, 4H, H-13-14), 1.35 (m, 2H, H-12), 1.43 (m, 2H, H-11), 1.77 (m, 2H, H-10), 2.87 (m, 2H, H-9), 7.42 (s, br, -OH), 7.47 (dd, 1H, *J* = 8.2 Hz, *J* = 4.5 Hz, H-6), 8.77 (dd, 1H, *J* = 8.2 Hz, *J* = 1.8 Hz, H-5), 8.79 (dd, 1H, *J* = 4.5 Hz, *J* = 1.8 Hz, H-7). ^{13}C-NMR ($CDCl_3$ 100.53 MHz) δ (ppm): 14.2 (C-15), 22.7, 29.0, 29.1, 29.3, 31.8 (C-10-14), 31.2 (C-9), 121.8 (C-6), 126.1 (C-4a), 130.9 (C-2), 137.1 (C-5), 144.3 (C-3), 152.5 (C-7), 158.6 (C-8a), 174.6 (C-4). ESI-HRMS: m/z = 278.1208 $[M+H]^+$, calc. for $C_{15}H_{19}NO_2S + H^+$ = 278.1209.

2-heptyl-3-hydroxy-4*H*-thiopyrano[3,2-c]pyridin-4-one (**10**)

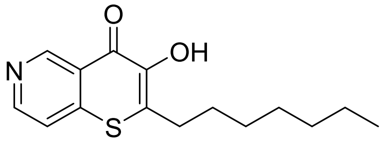

The product was obtained as yellow solid after purification by column chromatography using petrol ether/ethyl acetate 3:1 (33% Yield). R_f = 0.2 (PE/EA 3:1). ^{1}H-NMR ($CDCl_3$ 400.1 MHz) δ (ppm): 0.88 (m, 3H, H-15), 1.23 – 1.31 (m, 4H, H-13 and H-14), 1.34 (m, 2H, H-12), 1.41 (m, 2H, H-11), 1.74 (m, 2H, H-10), 2.83 (m, 2H, H-9), 7.50 (d, 1H, *J* = 5.4 Hz, H-8), 7.61 (s, br, -OH), 8.64 (s, br, 1H, H-7), 9.69 (s, br, 1H, H-5). ^{13}C-NMR ($CDCl_3$ 100.6 MHz) δ (ppm): 14.2 (C-15), 22.8, 28.9, 29.1, 29.3, 31.8 (C-10-14), 31.2 (C-9), 119.8 (C-8), 124.0 (C-4a), 128.9 (C-2), 145.7 and 146.0 (C-3 and C8a), 148.2 (C-7), 173.8 (C-4). ESI-HRMS: m/z = 278.1206 $[M+H]^+$, calc. for $C_{15}H_{19}NO_2S + H^+$ = 278.1209.

9. Synthesis of 2-heptyl-3-hydroxy-4*H*-thiochromen-4-one 1-oxide (**5**) and 2-heptyl-3-hydroxy-4H-thiochromen-4-one 1,1-dioxide (**6**)

2-Heptyl-3-hydroxy-4H-thiochromen-4-one (**3**) (205 mg, 0.9 mmol) was dissolved in 15 ml DCM and 200 mg mCPBA (1 eq.) was added. The reaction was stirred at room temperature for 1 h and quenched by the addition of water, extracted with DCM and the combined organic phases were washed with brine, dried with $MgSO_4$, filtered and the solvent was evaporated. The residue was pre-purified by column chromatography using petrol ether/ethyl acetate 2:1 which resulted in 3 fraction. The first fraction contained the clean educt (m = 73.9 mg, 36%). Fraction number 2 and 3 contained the mono- and dioxidized products (**5**) and (**6**) respectively. The second and third fraction were separately purified by column chromatography using DCM/MeOH mixtures.

2-heptyl-3-hydroxy-4*H*-thiochromen-4-one 1-oxide (**5**)

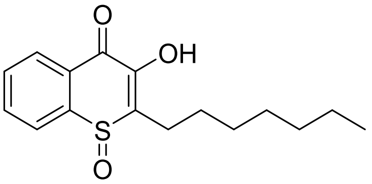

The product was obtained as slightly yellow solid after purification by column chromatography using DCM/MeOH 97:3 (12% Yield). R_f = 0.36 (DCM/MeOH 97:3). ^{1}H-NMR ($CDCl_3$ 400.1 MHz) δ (ppm): 0.88 (m, 3H, H-15), 1.22 – 1.33 (m, 4H, H-13 and H-14), 1.37 (m, 2H, H-12), 1.46 (m, 2H, H-11), 1.79 (m, 2H, H-10), 2.91 (m, 2H, H-9), 7.36 (s, 1H, OH), 7.72 (t, *J* = 7.6 Hz, 1H, H-6), 7.85 (t, *J* = 7.6 Hz, 1H, H-7), 8.13 (d, *J* = 8.0 Hz, 1H, H-8), 8.35 (d, *J* = 7.8 Hz, 1H, H-5). ^{13}C-NMR ($CDCl_3$ 100.6 MHz) δ (ppm): 14.2 (C-15), 22.8, 29.1, 31.8 (3C, C-12 – C14), 26.8 (C-9), 28.3 (C-10), 29.6 (C-11), 126.2 (C-4a), 129.0 (C-8), 129.1 (C-5), 131.7 (C-6), 134.3 (C-7), 134.5 (C-2), 144.4 (C-3), 146.1 (C-8a), 176.0 (C-4). ESI-HRMS: m/z =

293.1205 [M+H]$^+$, calc. for $C_{16}H_{20}O_3S + H^+ = 293.1206$, m/z = 291.1064 [M-H]$^-$, calc. for $C_{16}H_{20}O_3S - H^+ =$ 291.1060.

2-heptyl-3-hydroxy-4*H*-thiochromen-4-one 1,1-dioxide (**6**)

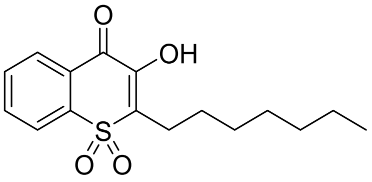

The product was obtained as white solid after purification by column chromatography using DCM/MeOH 99:1 (25% Yield). $R_f = 0.67$ (DCM/MeOH 99:1). ^{1}H-NMR ($CDCl_3$ 399.79 MHz) δ (ppm): 0.87 (m, 3H, H-15), 1.23 – 1.40 (H-12 – H-14), 1.40 – 1.51 (m, 2H, H-11), 1.76 – 1.87 (m, 2H, H-10), 2.76 (m, 2H, H-9), 7.27 (s, 1H, OH), 7.75 (t, *J* = 7.7 Hz, 1H, H-6), 7.89 (t, *J* = 7.6 Hz, 1H, H-7), 8.10 (d, *J* = 8.0 Hz, 1H, H-8), 8.28 (d, *J* = 7.8 Hz, 1H, H-5). ^{13}C-NMR ($CDCl_3$ 100.53 MHz) δ (ppm): 14.2 (C-15), 22.8 (C-12 – C-14), 23.7 (C-9), 28.4 (C-10), 29.0 (C-12 – C-14), 29.8 (C-11), 31.9 (C-12 – C-14), 123.9 (C-8), 125.6 (C-4a), 128.6 (C-5), 129.6 (C-2), 133.0 (C-6), 135.7 (C-7), 143.3 (C-3), 145.8 (C-8a), 176.0 (C-4). ESI-HRMS: m/z = 307.1011 [M-H]$^-$, calc. for $C_{16}H_{20}O_4S - H^+ = 307.1010$.

10. General synthesis of 3-((phenyl)thio)propanoic acids (**11a** – **14a**)

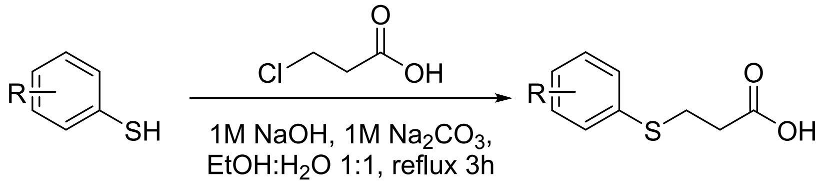

The substituted thiophenol (1 eq.) was dissolved in ethanol (15 ml/ 7 mmol thiophenol) and added to a mixture of 1 M NaOH (5.4 ml/7 mmol thiophenol) and 1 M Na_2CO_3 (5.4 ml/7 mmol thiophenol). The mixture was stirred for 5 min at room temperature and 3-chloropropanoic acid (1.02 eq.) in H_2O (4 ml/7 mmol acid) was added. The mixture was stirred for 3 h under reflux. After the reaction was allowed to cool to room temperature, the ethanol was evaporated under reduced pressure and the water phase acidified to pH = 1 with 6 M HCl solution. The purification procedure varied and is described for the individual compounds.

3-((2,5-dimethylphenyl)thio)propanoic acid (**11a**)

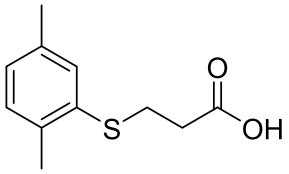

After acidification, the mixture was extracted with DCM and the combined organic layers washed with brine, dried with Na_2SO_4, filtered and the solvent evaporated. The residue was purified by column chromatography on silica gel with petrol ether/ethyl acetate 4:1 and 1:1. The compound was obtained as white solid (m = 1.21 g, 80.7%). $R_f = 0.37$ (petrol ether/ethyl acetate 1:1). ^{1}H-NMR ($CDCl_3$ 400.13 MHz) δ (ppm): 2.31 (s, 3H, C5-C**H**$_3$), 2.35 (s, 3H, C2-C**H**$_3$), 2.68 (t, 2H, *J* = 7.4 Hz, -S-CH$_2$-C**H**$_2$-), 3.13 (t, 2H, *J* = 7.4 Hz, -S-C**H**$_2$-CH$_2$-), 6.95 (m, 1H, H-4), 7.07 (d, 1H, *J* = 7.7 Hz, H-3), 7.14 (m, 1H, H-6). ^{13}C-NMR ($CDCl_3$ 100.54 MHz) δ (ppm): 20.1 (C2-CH$_3$), 21.1 (C5-CH$_3$), 28.3 (-S-**C**H$_2$-CH$_2$-), 34.1 (-S-CH$_2$-**C**H$_2$-), 127,7 (C-4), 130.36 (C-6), 130.44 (C-3), 133.9 (C-1), 135.8 (C-2), 136.3 (C-5), 177.5 (COOH).

3-((4-bromophenyl)thio)propanoic acid (**12a**)

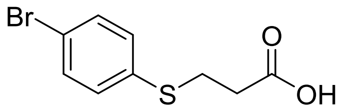

After acidification, the white precipitate was collected by filtration, washed with water and petrol ether and dried under vacuum to obtain the product as white solid (m = 1.85 g, 99.2%). ^{1}H-NMR ($CDCl_3$ 400.13 MHz) δ (ppm): 2.67 (t, 2H, *J* = 7.3 Hz, -S-CH_2-C**H**$_2$-), 3.14 (t, 2H, *J* = 7.3 Hz, -S-C**H**$_2$-CH_2-), 7.24 (m, 2H, H-2), 7.42 (m, 2H, H-3). ^{13}C-NMR ($CDCl_3$ 100.54 MHz) δ (ppm): 29.1 (-S-**C**H_2-CH_2-), 34.1 (-S-CH_2-**C**H_2-), 121.0 (C-4), 132.0 (C-2), 132.2 (C-3), 134.3 (C-1), 177.1 (COOH).

3-(naphthalen-2-ylthio)propanoic acid (**13a**)

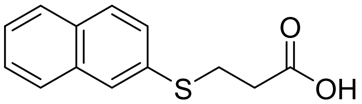

After acidification, the white precipitate was collected by filtration, washed with water and petrol ether and dried under vacuum to obtain the product as white solid (m = 1.64 g, 99.0%). ^{1}H-NMR ($CDCl_3$ 400.13 MHz) δ (ppm): 2.72 (t, 2H, *J* = 7.4 Hz), 3.26 (t, 2H, *J* = 7.4 Hz), 7.40 – 7.54 (m, 3H), 7.74 – 7.87 (m, 4H). ^{13}C-NMR ($CDCl_3$ 100.54 MHz) δ (ppm): 29.0 (-S-**C**H_2-CH_2-), 34.2 (-S-CH_2-**C**H_2-), 126.2, 126.8, 127.4, 127.7, 128.1, 128.7, 128.9, 132.3, 132.5, 133.9 (10C, naphthalene), 177.1 (COOH).

3-((3-methoxyphenyl)thio)propanoic acid (**14a**)

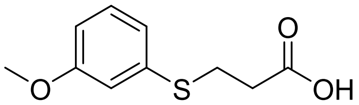

After acidification, the mixture was extracted with DCM and the combined organic layers were washed with brine, dried with Na_2SO_4, filtered and the solvent was evaporated. The residue was purified by column chromatography on silica gel with petrol ether/ethyl acetate 4:1 and 1:1. The compound was obtained as slightly yellow oil (m = 2.98 g, 98.4%). R_f = 0.5 (petrol ether/ethyl acetate 1:1). ^{1}H-NMR ($CDCl_3$ 400.13 MHz) δ (ppm): 2.69 (t, 2H, *J* = 7.4 Hz, -S-CH_2-C**H**$_2$-COOH), 3.16 (t, 2H, *J* = 7.4 Hz, -S-C**H**$_2$-CH_2-COOH), 6.76 (dd, 1H, *J* = 8.3 Hz, *J* = 2.2 Hz, H-4), 6.91 (m, 1H, H-2), 6.95 (d, 1H, *J* = 7.6 Hz, H-6), 7.21 (dd, 1H, *J* = 8.3 Hz, *J* = 7.6 Hz, H-5). ^{13}C-NMR ($CDCl_3$ 100.54 MHz) δ (ppm): 28.7 (-S-**C**H_2-CH_2-COOH), 34.3 (-S-CH_2-**C**H_2-COOH), 112.6 (C-4), 115.6 (C-2), 122.3 (C-6), 130.0 (C-5), 136.4 (C-1), 160.1 (C-3), 177.9 (COOH).

11. General synthesis of thiochroman-4-ones (**11b** – **14b**)

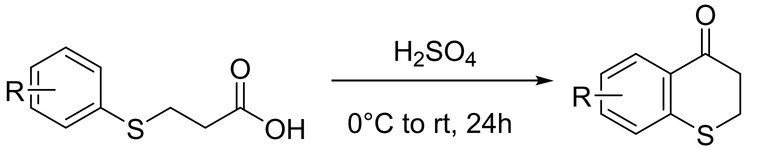

The substituted 3-(phenylthio)propanoic acid was added portion wise to conc. H_2SO_4 (10 ml/5 mmol 3-(phenylthio)thiopropanoic acid) at 0°C. The mixture was stirred at 0°C for 20 min and afterwards 24 h at room temperature. The reaction was poured on ice and extracted with DCM. The combined organic layers were washed with brine, dried with Na_2SO_4, filtered and the solvent evaporated. The residues were purified as described for the individual compounds.

5,8-dimethylthiochroman-4-one (**11b**)

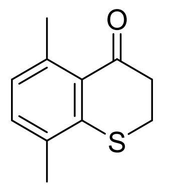

The residue was purified by column chromatography on silica gel with petrol ether/ethyl acetate 9:1. The product was obtained as slightly yellow oil (m = 340 mg, 33.5%). R_f = 0.57 (ether/ethyl acetate 9:1). ^{1}H-NMR ($CDCl_3$ 400.13 MHz) δ (ppm): 2.28 (s, 3H, C8-CH_3), 2.56 (s, 3H, C5-CH_3), 2.97 (m, 2H, H-3), 3.18 (m, 2H, H-2), 6.88 (d, 1H, *J* = 7.6 Hz, H-6), 7.12 (d, 1H, *J* = 7.6 Hz, H-7). ^{13}C-NMR ($CDCl_3$ 100.54 MHz) δ (ppm): 20.3 (C8-**C**H_3), 23.8 (C5-**C**H_3), 25.8 (C-2), 41.3 (C-3), 128.4 (C-6), 130.5 (C-4a), 133.18 (C-8), 133.24 (C-7), 140.2 (C-5), 142.3 (C-8a), 197.1 (C-4).

6-bromothiochroman-4-one (**12b**)

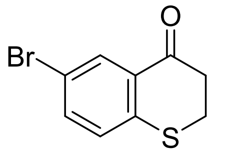

The residue was purified by column chromatography on silica gel with petrol ether/DCM 1:1. The product was obtained as yellow solid (m = 770 mg, 58.6%), R_f = 0.32 (petrol ether/DCM 1:1). ^{1}H-NMR ($CDCl_3$ 400.13 MHz) δ (ppm): 2.97 (m, 2H, H-3), 3.24 (m, 2H, H-2), 7.16 (d, 1H, *J* = 8.5 Hz, H-8), 7.47 (dd, 1H, *J* = 8.5 Hz, *J* = 2.3 Hz, H-7), 8.22 (d, 1H, *J* = 2.3 Hz, H-5). ^{13}C-NMR ($CDCl_3$ 100.54 MHz) δ (ppm): 26.6 (C-2), 39.3 (C-3), 118.9 (C-4a), 129.4 (C-8), 132.0 (C-5), 132.3 (C-6), 136.1 (C-7), 141.3 (C-8a), 192.8 (C-4).

2,3-dihydro-1*H*-benzo[*f*]thiochromen-1-one (**13b**)

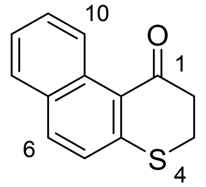

The residue was purified by column chromatography on silica gel with petrol ether/DCM 1:1. The product was obtained as yellow oil (m = 405 mg, 33.7%). R_f = 0.13 (petrol ether/DCM 1:1). ^{1}H-NMR ($CDCl_3$ 400.13 MHz) δ (ppm): 3.11 (t, 2H, *J* = 7.4 Hz, H-3), 3.29 (t, 2H, *J* = 6.3 Hz, H-2), 7.28 (d, 2H, *J* = 8.7 Hz, H-5), 7.45 (m, 1H, H-8), 7.60 (m, 1H, H-9), 7.74 (d, 1H, *J* = 8.1 Hz, H-7), 7.77 (d, 1H, *J* = 8.8 Hz, H-6), 9.16 (d, 1H, *J* = 8.8 Hz, H-10). ^{13}C-NMR ($CDCl_3$ 100.54 MHz) δ (ppm): 26.5 (C-3), 41.4 (C-2), 125.5 (C-5), 125.8 (2C, C-10b, C-9), 126.2 (C-10), 128.6 (C-7), 129.3 (C-8), 131.9 (C-6a), 132.6 (C-10a), 133.7 (C-6), 145.3 (C-4a), 196.3 (C-1).

7-methoxythiochroman-4-one (**14b**)

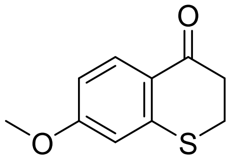

The residue was purified by column chromatography on silica gel with petrol ether/ethyl acetate 4:1 and 1:1. Two fractions were collected. The first fraction was obtained with PE/EA 4:1 as yellow solid and identified as 7-methoxythiochroman-4-one (m = 504 mg, 18.9%). R_f = 0.38 (petrol ether/ethyl acetate 4:1). ^{1}H-NMR ($CDCl_3$ 400.13 MHz) δ (ppm): 2.92 (m, 2H, H-3), 3.22 (m, 2H, H-2), 3.83 (s, -CH_3), 6.70 (dd, 1H, *J* = 8.7 Hz, *J* = 2.3 Hz, H-6), 6.72 (d, 1H, *J* = 2.3 Hz, H-8), 8.07 (d, 1H, *J* = 8.7 Hz, H-5). ^{13}C-NMR ($CDCl_3$ 100.54 MHz) δ (ppm): 27.0 (C-2), 39.5 (C-3), 55.7 (-CH_3) 110.9 (C-8), 112.7 (C-6), 124.8 (C-4a), 131.5 (C-5), 144.5 (C-8a), 163.3 (C-7), 193.0 (C-4). The second fraction was obtained with PE/EA 3:2 as yellow oil and identified as 5-methoxythiochroman-4-one (m = 166 mg, 6.2%). R_f = 0.4 (petrol ether/ethyl acetate 3:2). ^{1}H-NMR ($CDCl_3$ 400.13 MHz) δ (ppm): 2.97 (m, 2H, H-3), 3.17 (m, 2H, H-2), 3.88 (s, -CH_3), 6.70 (d, 1H, *J* = 8.3 Hz, H-6), 6.85 (d, 1H, *J* = 8.0 Hz, H-8), 7.26 (dd, 1H, *J* = 8.0 Hz, 8.3 Hz, H-7). ^{13}C-NMR ($CDCl_3$ 100.54 MHz) δ (ppm): 26.5 (C-2), 41.7 (C-3), 56.2 (-CH_3) 108.8 (C-6), 119.9 (C-8), 121.5 (C-4a), 133.6 (C-7), 144.3 (C-8a), 161.4 (C-5), 194.2 (C-4).

12. General synthesis of thiochromen-4-ones (**11c** – **14c**)

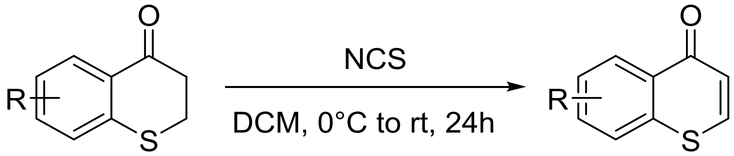

The thiochoman-4-one (1eq.) was dissolved in 10 ml DCM and cooled in an ice-bath. To the solution was added *N*-chlorosuccinimide (NCS, 1 eq.) and the mixture was stirred for 20 min at 4°C. The mixture was allowed to reach room temperature and was stirred for 24 h. The reaction was quenched by the addition of water and the water phase extracted with DCM. The combined organic layers were washed with brine, dried with Na_2SO_4, filtered and the solvent was evaporated. The residues were purified as described for the individual compounds.

5,8-dimethyl-4*H*-thiochromen-4-one (**11c**)

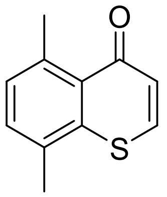

The residue was purified by column chromatography on silica gel using DCM and DCM + 1% MeOH. The product was eluted with DCM + 1% MeOH and obtained as off-white crystalline solid (m = 224 mg, 68.6%). R_f = 0.125 (DCM). ^{1}H-NMR ($CDCl_3$ 400.13 MHz) δ (ppm): 2.47 (s, 3H, C8-CH_3), 2.85 (s, 3H, C5-CH_3), 7.00 (d, 1H, *J* = 10.4 Hz, H-3), 7.20 (d, 1H, *J* = 7.6 Hz, H-6), 7.31 (d, 1H, *J* = 7.6 Hz, H-7), 7.72 (d, 1H, *J* = 10.4 Hz, H-2). ^{13}C-NMR ($CDCl_3$ 100.54 MHz) δ (ppm): 20.0 (C8-**C**H_3), 25.1 (C5-**C**H_3), 127.6 (C-3), 130.9

(C-6), 131.2 (C-4a), 132.1 (C-7), 132.6 (C-8), 135.3 (C-2), 138.8 (C-8a), 141.2 (C-5), 183.0 (C-4). ESI-HRMS: m/z = 191.0523 $[M+H]^+$, calc. for $C_{11}H_{10}OS + H^+$ = 191.0525.

6-bromo-4*H*-thiochromen-4-one (**12c**)

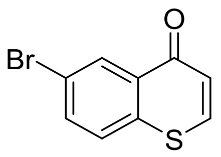

The residue was purified by column chromatography on silica gel using DCM. The product was obtained as pink crystalline solid (m = 558 mg, 80.4%). R_f = 0.22 (DCM). ^{1}H-NMR ($CDCl_3$ 400.13 MHz) δ (ppm): 7.02 (d, 1H, *J* = 10.4 Hz, H-3), 7.48 (d, 1H, *J* = 8.6 Hz, H-8), 7.70 (dd, 1H, *J* = 8.6 Hz, *J* = 2.1 Hz, H-7), 7.83 (d, 1H, *J* = 10.4 Hz, H-2), 8.67 (d, 1H, *J* = 2.1 Hz, H-5). ^{13}C-NMR ($CDCl_3$ 100.54 MHz) δ (ppm): 122.4 (C-6), 126.0 (C-3), 128.4 (C-8), 131.6 (C-5), 133.7 (C-4a), 134.7 (C-7), 136.3 (C-8a), 137.9 (C-2), 178.5 (C-4). ESI-HRMS: m/z = 240.9318 $[M+H]^+$, calc. for $C_9H_5{}^{79}BrOS + H^+$ = 240.9317, m/z = 242.9291 $[M+H]^+$, calc. for $C_9H_5{}^{81}BrOS + H^+$ = 242.9297.

1*H*-benzo[*f*]thiochromen-1-one (**13c**)

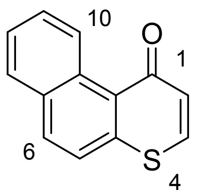

The residue was purified by column chromatography on silica gel using DCM + 1% MeOH. Two fractions were isolated. Fraction 1 was obtained as yellow crystalline solid and identified as the product (m = 333 mg, 86.7%), R_f = 0.175 (DCM + 1% MeOH). ^{1}H-NMR ($CDCl_3$ 400.13 MHz) δ (ppm): ^{1}H-NMR ($CDCl_3$ 400.13 MHz) δ (ppm): 7.18 (d, 1H, *J* = 10.5 Hz, H-2), 7.55 (d, 1H, *J* = 8.7 Hz, H-5), 7.64 (m, 1H, H-8), 7.75 (m, 1H, H-9), 7.77 (d, 1H, *J* = 10.5 Hz, H-3), 7.89 (d, 1H, *J* = 7.8 Hz, H-7), 7.89 (d, 1H, *J* = 8.7 HZ, H-6), 10.13 (d, 1H, *J* = 8.9 HZ, H-10). ^{13}C-NMR ($CDCl_3$ 100.54 MHz) δ (ppm): 123.9 (C-5), 127.4 (C-10), 127.6 (C-8), 127.7 (C-10b), 128.5 (C-7), 129.2 (C-9), 129.8 (C-2), 132.5 (C-6a), 132.8 (C-10a), 133.0 (C-6), 133.9 (C-3), 140.7 (C-4a), 182.1 (C-1). ESI-HRMS: m/z = 213.0366 $[M+H]^+$, calc. for $C_{13}H_8OS + H^+$ = 213.0369.

7-methoxy-4*H*-thiochromen-4-one (**14c**)

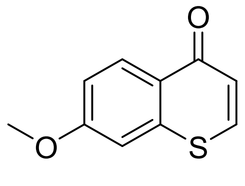

The residue was purified by column chromatography on silica gel using DCM + 2% MeOH. The product was obtained as of-white solid and identified as the product (m = 443 mg, 90.6%), R_f = 0.25 (DCM + 2% MeOH). ^{1}H-NMR ($CDCl_3$ 400.13 MHz) δ (ppm): 3.90 (s, 3H, $-OCH_3$), 6.96 (d, 1H, *J* = 10.5 Hz, H-3), 7.00 (d, 1H, *J* = 2.4 Hz, H-8), 7.09 (dd, 1H, *J* = 9.0 Hz, *J* = 2.4 Hz, H-6), 7.72 (d, 1H, *J* = 10.5 Hz, H-2), 8.47 (d, 1H, *J* = 9.0 Hz, H-5). ^{13}C-NMR ($CDCl_3$ 100.54 MHz) δ (ppm): 55.8 ($-OCH_3$), 110.1 (C-8), 116.8 (C-6), 126.1 (C-3), 126.3 (C-4a), 130.9 (C-5), 137.1 (C-2), 139.9 (C-8a), 161.9 (C-7), 179.3 (C-4). ESI-HRMS: m/z = 193.0315 $[M+H]^+$, calc. for $C_{10}H_8O_2S + H^+$ = 193.0318.

13. General synthesis of 2-heptylthiochroman-4-ones (**11d** – **14d**)

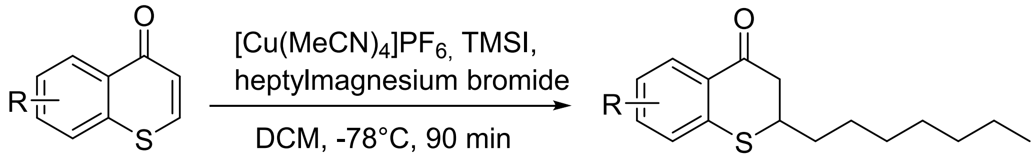

A solution of heptylmagnesium bromide was prepared by adding 1.5 eq. 1-bromoheptane to 1.575 eq. magnesium in dry diethyl ether (1.0 ml / 1.0 mmol 1-bromoheptane) under nitrogen. The reaction started spontaneously and was complete within 20 min. Separately, 1 eq. thiochroman-4-one was dissolved in dry DCM (20.0 ml / 1.0 mmol thiochroman-4-one) under nitrogen, cooled to -78°C and 0.05 eq. $[Cu(MeCN)_4]PF_6$ were added. The reaction was stirred for 10 min at -78°C. To this mixture were added simultaneously the aforementioned reaction mixture with heptylmagnesium bromide and 3 eq. trimethylsilyl iodide (TMSI). The reaction was kept at -78°C for 90 min and afterwards quenched by the addition of 10% HCl solution at -78°C. After reaching room temperature, the mixture was extracted three times with DCM, the combined organic phases were washed with sat. $Na_2S_2O_3$ and brine, dried with Na_2SO_4, filtered and the solvent evaporated. The residues were purified as described for the individual compound.

2-heptyl-5,8-dimethylthiochroman-4-one (**11d**)

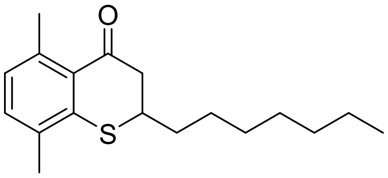

The residue was purified by column chromatography on silica gel using n-hexane/DCM 1:1. The product was obtained as white solid, 82.6% yield, R_f = 0.45 (hexane/DCM 1:1). ^{1}H-NMR ($CDCl_3$ 400.13 MHz) δ (ppm): 0.89 (m, 3H, H-15), 1.21 – 1.38 (m, 8H, H-11-14), 1.48 (m, 2H, H-10), 1.72 (m, 2H, H-9), 2.28 (s, 3H, C8-CH_3), 2.56 (s, 3H, C5-CH_3), 2.79 (dd, 1H, *J* = 15.0 Hz, *J* = 11.1 Hz, H-3a), 3.00 (dd, 1H, *J* = 15.0 Hz, *J* = 3.2 Hz, H-3b), 3.42 (m, 1H, H-2), 6.86 (d. 1H, *J* = 7.7 Hz, H-6), 7.11 (d, 1H, *J* = 7.7 Hz, H-7). ^{13}C-NMR ($CDCl_3$ 100.54 MHz) δ (ppm): 14.2 (C-15), 20.3 (C8-CH_3), 23.6 (C5-CH_3), 22.7, 29.2, 29.4, 31.9 (4C, C-11-14), 26.7 (C-10), 35.0 (C-9), 40.3 (C-2), 47.7 (C-3), 128.0 (C-6), 130.1 (C-4a), 133.0 (C-8), 133.3 (C-7), 139.8 (C-5), 141.8 (C-8a), 197.8 (C-4). ESI-HRMS: m/z = 291.1771 $[M+H]^+$, calc. for $C_{18}H_{26}OS + H^+$ = 291.1777.

6-bromo-2-heptylthiochroman-4-one (**12d**)

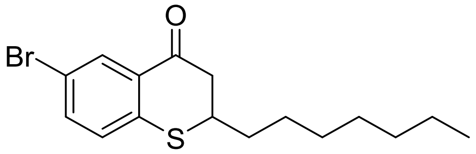

The residue was purified by column chromatography on silica gel using n-hexane/DCM 1:1. The product was obtained as white solid, 80.8% yield, R_f = 0.375 (hexane/DCM 2:1). ^{1}H-NMR ($CDCl_3$ 400.13 MHz) δ (ppm): 0.88 (m, 3H, H-15), 1.20 – 1.35 (m, 8H, H-11-14), 1.45 (m, 2H, H-10), 1.70 (m, 2H, H-9), 2.78 (dd, 1H, *J* = 16.3 Hz, *J* = 11.1 Hz, H-3a), 3.04 (dd, 1H, *J* = 16.3 Hz, J = 3.1 Hz, H-3b), 3.48 (m, 1H, H-2), 7.14 (d, 1H, *J* = 8.5 Hz, H-8), 7.47 (d, 1H, *J* = 8.5 Hz, *J* = 2.3 Hz, H-7), 8.19 (d, 1H, *J* = 2.3 Hz, H-5). ^{13}C-NMR ($CDCl_3$ 100.54 MHz) δ (ppm): 14.2 (C-15), 22.7, 29.2, 29.3, 31.8 (4C, C-11-14), 26.8 (C-10), 34.5 (C-9), 41.8 (C-2), 45.9 (C-3), 118.7 (C-6), 129.4 (C-8), 131.7 (C-5), 132.0 (C-4a), 136.3 (C-7), 140.8 (C-8a),

193.6 (C-4). ESI-HRMS: m/z = 341.0558 [M+H]$^+$, calc. for $C_{16}H_{21}{}^{79}BrOS + H^+$ = 341.0569, m/z = 343.0540 [M+H]$^+$, calc. for $C_{16}H_{21}{}^{81}BrOS + H^+$ = 343.0549.

3-heptyl-2,3-dihydrobenzo[*f*]thiochromen-1-one (**13d**)

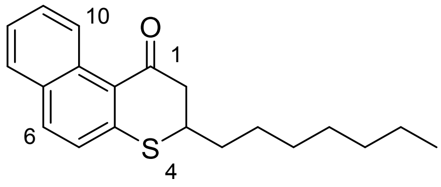

The residue was purified by column chromatography on silica gel using n-hexane/DCM 1:1. The product was obtained as yellow oil, 74.4% yield, R_f = 0.25 (hexane/DCM 1:1). ^{1}H-NMR ($CDCl_3$ 400.13 MHz) δ (ppm): 0.89 (m, 3H, H-15), 1.21 – 1.40 (m, 8H, H-11-14), 1.49 (m, 2H, H-10), 1.77 (m, 2H, H-9), 2.94 (dd, 1H, *J* = 15.4 Hz, *J* = 11.4 Hz, H-2a), 3.16 (dd, 1H, *J* = 15.4 Hz, *J* = 3.3 Hz, H-2b), 3.57 (m, 1H, H-3), 7.26 (d. 1H, *J* = 8.7 Hz, H-5), 7.44 (m, 1H, H-8), 7.59 (m, 1H, H-9), 7.74 (d, 1H, *J* = 8.1 Hz, H-7), 7.77 (d, 1H, *J* = 8.7 Hz, H-6), 9.20 (d, 1H, *J* = 8.8 Hz, H-10). ^{13}C-NMR ($CDCl_3$ 100.54 MHz) δ (ppm): 14.2 (C-15), 22.8, 29.2, 29.3, 31.9 (4C, C-11-14), 26.8 (C-10), 34.6 (C-9), 41.4 (C-3), 47.9 (C-2), 125.2 (C-10b), 125.5 (C-5), 125.7 (C-8), 126.0 (C-10), 128.5 (C-7), 129.3 (C-9), 131.8 (C-6a), 132.4 (C-10a), 133.8 (C-6), 144.9 (C-4a), 197.1 (C-1). ESI-HRMS: m/z = 313.1611 [M+H]$^+$, calc. for $C_{20}H_{24}OS + H^+$ = 313.1621.

2-heptyl-7-methoxythiochroman-4-one (**14d**)

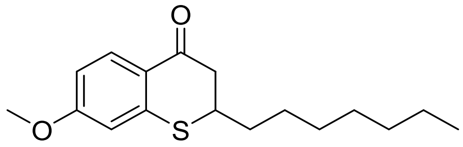

The residue was purified by column chromatography on silica gel using DCM. The product was obtained as yellow oil product, 90.6% yield, R_f = 0.425 (DCM). ^{1}H-NMR ($CDCl_3$ 400.13 MHz) δ (ppm): 0.87 (m, 3H, H-15), 1.20 – 1.35 (m, 8H, H-11-14), 1.45 (m, 2H, H-10), 1.69 (m, 2H, H-9), 2.73 (dd, 1H, *J* = 16.4 Hz, *J* = 11.1 Hz, H-3a), 2.98 (dd, 1H, *J* = 16.4 Hz, *J* = 3.1 Hz, H-3b), 3.47 (m, 1H, H-2), 6.69 (dd, 1H, *J* = 8.7 Hz, *J* = 2.5 Hz, H-6), 6.71 (d, 1H, *J* = 2.5 Hz, H-8), 8.04 (d, 1H, *J* = 8.7 Hz, H-5). ^{13}C-NMR ($CDCl_3$ 100.54 MHz) δ (ppm): 14.2 (C-15), 22.7, 29.2, 29.3, 31.8 (4C, C-11-14), 26.8 (C-10), 34.6 (C-9), 42.0 (C-2), 46.2 (C-3), 55.6 (-OCH_3), 110.9 (C-8), 112.5 (C-6), 124.5 (C-4a), 131.2 (C-5), 144.1 (C-8a), 163.5 (C-7), 193.6 (C-4). ESI-HRMS: m/z = 293.1563 [M+H]$^+$, calc. for $C_{17}H_{24}O_2S + H^+$ = 293.1570.

<u>14. General synthesis of 2-heptyl-3-hydroxy-4*H*-thiochromen-4-ones (**11 – 14**)</u>

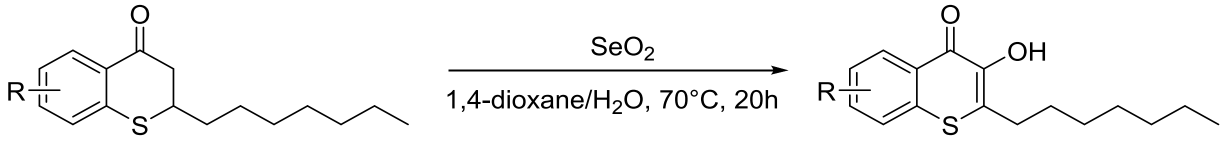

The 2-heptyl-thiochroman-4-one (1 eq.) was dissolved in 1,4-dioxane (6 ml / 1 mmol 2-heptyl-thiochroman-4-one) and H_2O (30 µl / 1 ml 1,4-dioxane) and SeO_2 (1.1 eq.) was added. The mixture was heated to 70 °C and stirred for 20 h. Afterwards the mixture was extracted three times with ethyl acetate, the combined organic phases washed brine, dried with Na_2SO_4, filtered and the solvent evaporated. The residues were purified as described for the individual compound.

2-heptyl-3-hydroxy-5,8-dimethyl-4*H*-thiochromen-4-one (**11**)

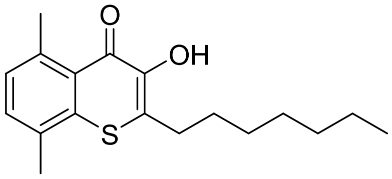

The residue was pre-purified by column chromatography on silica gel using n-hexane/ethyl acetate 9:1 which gave a mixture of product with the starting material that were inseparable by normal phase low pressure column chromatography, R_f = 0.8 (n-hexane/ethyl acetate 9:1). The compounds were separated by preparative RP-HPLC (Reprosil-Pur 120 C18-AQ, 10 µm, 250 x 20 mm, Dr. Maisch GmbH, A: H_2O + 0.1 % formic acid, B: MeOH + 0.1 % formic acid, 0-5 min 5% B, 5-30 min 5-95% B, 30-35 min 95% B). The product was obtained as yellow crystalline solid (15.3% yield). ^{1}H-NMR ($CDCl_3$ 800.2 MHz) δ (ppm): 0.89 (m, 3H, H-15), 1.25 – 1.32 (m, 4H, H-13-14), 1.35 (m, 2H, H-12), 1.42 (m, 2H, H-11), 1.76 (m, 2H, H-10), 2.49 (s, 3H, C8-CH_3), 2.82 (m, 2H, H-9), 2.92 (s, 3H, C5-CH_3), 2.79 (dd, 1H, *J* = 15.0 Hz, *J* = 11.1 Hz, H-3a), 3.00 (dd, 1H, *J* = 15.0 Hz, *J* = 3.2 Hz, H-3b), 3.42 (m, 1H, H-2), 7.17 (d. 1H, *J* = 7.4 Hz, H-6), 7.29 (d, 1H, *J* = 7.4 Hz, H-7). ^{13}C-NMR ($CDCl_3$ 201.2 MHz) δ (ppm): 14.2 (C-15), 19.6 (C8-CH_3), 24.8 (C5-CH_3), 22.8, 29.0, 29.2, 29.4, 31.9 (5C, C-10-14), 31.2 (C-9), 125.6 (C-2), 127.5 (C-4a), 129.6 (C-6), 131.4 (C-7), 132.0 (C-8), 138.8 (C-8a), 141.3 (C-5), 144.8 (C-3), 176.6 (C-4). ESI-HRMS: m/z = 305.1560 $[M+H]^+$, calc. for $C_{18}H_{24}O_2S + H^+$ = 305.1570.

6-bromo-2-heptyl-3-hydroxy-4*H*-thiochromen-4-one (**12**)

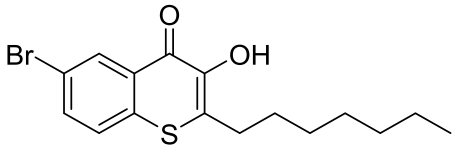

The residue was purified by column chromatography on silica gel using n-hexane/ethyl acetate 95:5. The product was obtained as yellow solid, 17.5% yield, R_f = 0.375 (n-hexane/ethyl acetate 95:5). ^{1}H-NMR ($CDCl_3$ 600.3 MHz) δ (ppm): 0.88 (m, 3H, H-15), 1.23 – 1.31 (m, 4H, H-13-14), 1.34 (m, 2H, H-12), 1.41 (m, 2H, H-11), 1.74 (m, 2H, H-10), 2.83 (m, 2H, H-9), 7.50 (d, 1H, *J* = 8.7 Hz, H-8), 7.51 (s, br, 1H, -OH), 7.66 (dd, 1H, *J* = 8.7 Hz, *J* = 1.9 HZ, H-7), 8.66 (d, 1H, *J* = 1.9 Hz, H-5). ^{13}C-NMR ($CDCl_3$ 150.9 MHz) δ (ppm): 14.3 (C-15), 22.8, 29.0, 29.1, 29.3, 31.8 (5C, C-10-14), 31.3 (C-9), 120.9 (C-6), 127.7 (C-8), 129.7 (C-2), 130.3 (C-4a), 131.4 (C-5), 133.9 (C-7), 136.5 (C-8a), 144.3 (C-3), 173.0 (C-4). ESI-HRMS: m/z = 355.0360 $[M+H]^+$, calc. for $C_{16}H_{19}{}^{79}BrO_2S + H^+$ = 355.0362, m/z = 357.0338 $[M+H]^+$, calc. for $C_{16}H_{19}{}^{81}BrO_2S + H^+$ = 357.0341.

3-heptyl-2-hydroxybenzo[*f*]-4*H*-thiochromen-1-one (**13**)

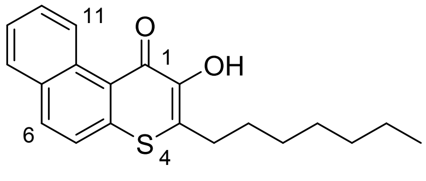

The residue was pre-purified by column chromatography on silica gel using n-hexane/ethyl acetate 95:5 which gave a mixture of product with the starting material that were inseparable by normal phase low pressure column chromatography (R_f = 0.4 (n-hexane/ethyl acetate 95:5). The compounds were separated by preparative RP-HPLC (Reprosil-Pur 120 C18-AQ, 10 µm, 250 x 20 mm, Dr. Maisch GmbH, A: H_2O + 0.1 % formic acid, B: MeOH + 0.1 % formic acid, 0-5 min 5% B, 5-30 min 5-95% B, 30-35 min

95% B). The product was obtained as yellow crystalline solid (2.7 % yield). ^{1}H-NMR ($CDCl_3$ 600.3 MHz) δ (ppm): 0.89 (m, 3H, H-17), 1.23 – 1.33 (m, 4H, H-15-16), 1.37 (m, 2H, H-14), 1.44 (m, 2H, H-13), 1.79 (m, 2H, H-12), 2.90 (m, 2H, H-11), 7.57 (d, 1H, *J* = 8.7 Hz, H-5), 7.66 (m, 1H, H-8), 7.78 (m, 1H, H-9), 7.91 (d, 1H, *J* = 8.0 Hz, H-7), 7.94 (d, 1H, *J* = 8.7 Hz, H-6), 8.41 (s, br, 1H, -OH), 10.26 (d, 1H, *J* = 8.6 Hz, H-10). ^{13}C-NMR ($CDCl_3$ 150.9 MHz) δ (ppm): 14.1 (C-17), 22.6, 28.96, 29.02, 29.2, 31.7 (5C, C-12-16), 30.8 (C-11), 123.1 (C-10b), 123.7 (C-5), 125.0 (C-3), 127.1 (C-10), 127.2 (C-8), 128.4 (C-7), 129.0 (C-9), 132.1 (C-6a), 132.2 (C-6), 132.5 (C-10a), 140.8 (C-4a), 146.6 (C-2), 175.1 (C-1). ESI-HRMS: m/z = 327.1406 $[M+H]^+$, calc. for $C_{20}H_{22}O_2S + H^+$ = 327.1413.

2-heptyl-3-hydroxy-7-methoxy-4*H*-thiochromen-4-one (**14**)

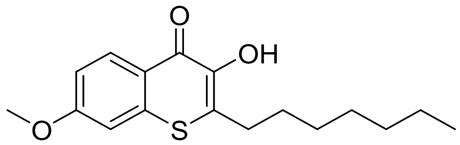

The residue was purified by column chromatography on silica gel using n-hexane/ethyl acetate 9:1. The product was obtained as yellow crystalline solid, 25.0% yield, R_f = 0.5 (n-hexane/ethyl acetate 9:1). ^{1}H-NMR ($CDCl_3$ 600.3 MHz) δ (ppm): 0.89 (m, 3H, H-15), 1.40 – 1.48 (m, 8H, H-11-14), 1.73 (m, 2H, H-10), 2.79 (m, 2H, H-9), 3.91 (s, 3H, $-OCH_3$), 7.00 (d, 1H, *J* = 2.4 Hz, H-8), 7.08 (dd, 1H, *J* = 9.1 Hz, *J* = 2.4 Hz, H-6), 8.43 (d, 1H, *J* = 9.1 Hz, H-5). ^{13}C-NMR ($CDCl_3$ 150.9 MHz) δ (ppm): 14.2 (C-15), 22.8, 29.0, 29.1, 29.3, 31.9 (5C, C-10-14), 31.1 (C-9), 55.8 ($-OCH_3$), 107.4 (C-8), 116.6 (C-6), 122.9 (C-4a), 127.3 (C-2), 130.9 (C-5), 140.5 (C-8a), 143.5 (C-3), 161.6 (C-7), 173.7 (C-4). ESI-HRMS: m/z = 307.1352 $[M+H]^+$, calc. for $C_{17}H_{22}O_3S + H^+$ = 307.1362.

4. NMR spectra

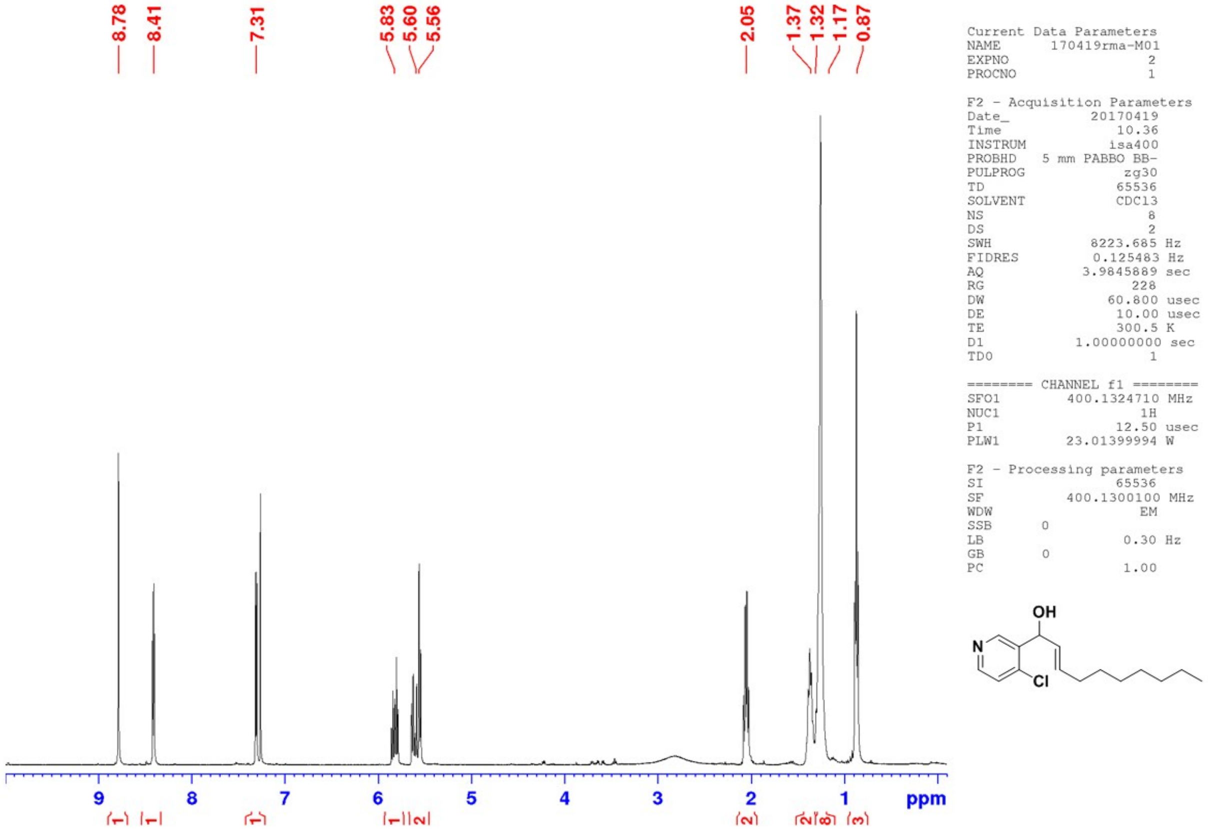

^{1}H-NMR spectra of (**10a**) in $CDCl_3$

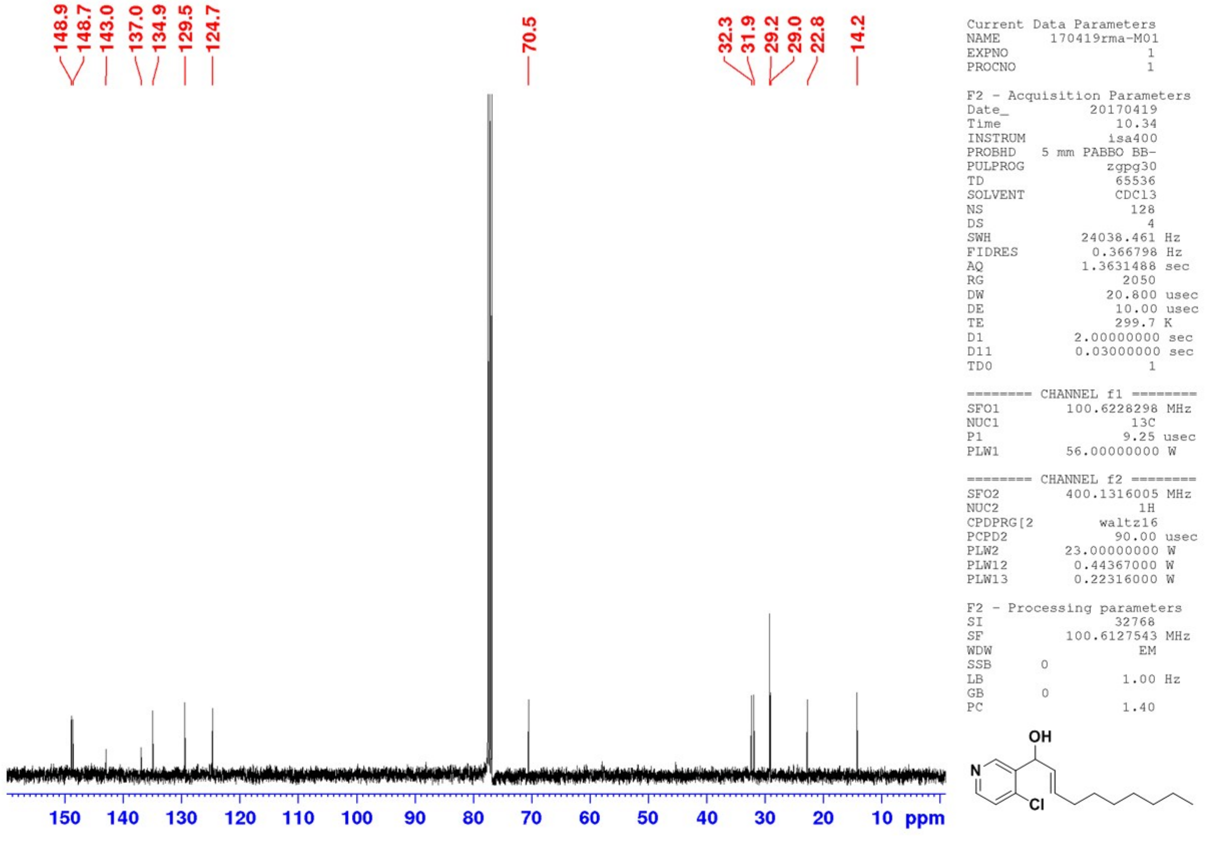

^{13}C-NMR spectra of (**10a**) in $CDCl_3$

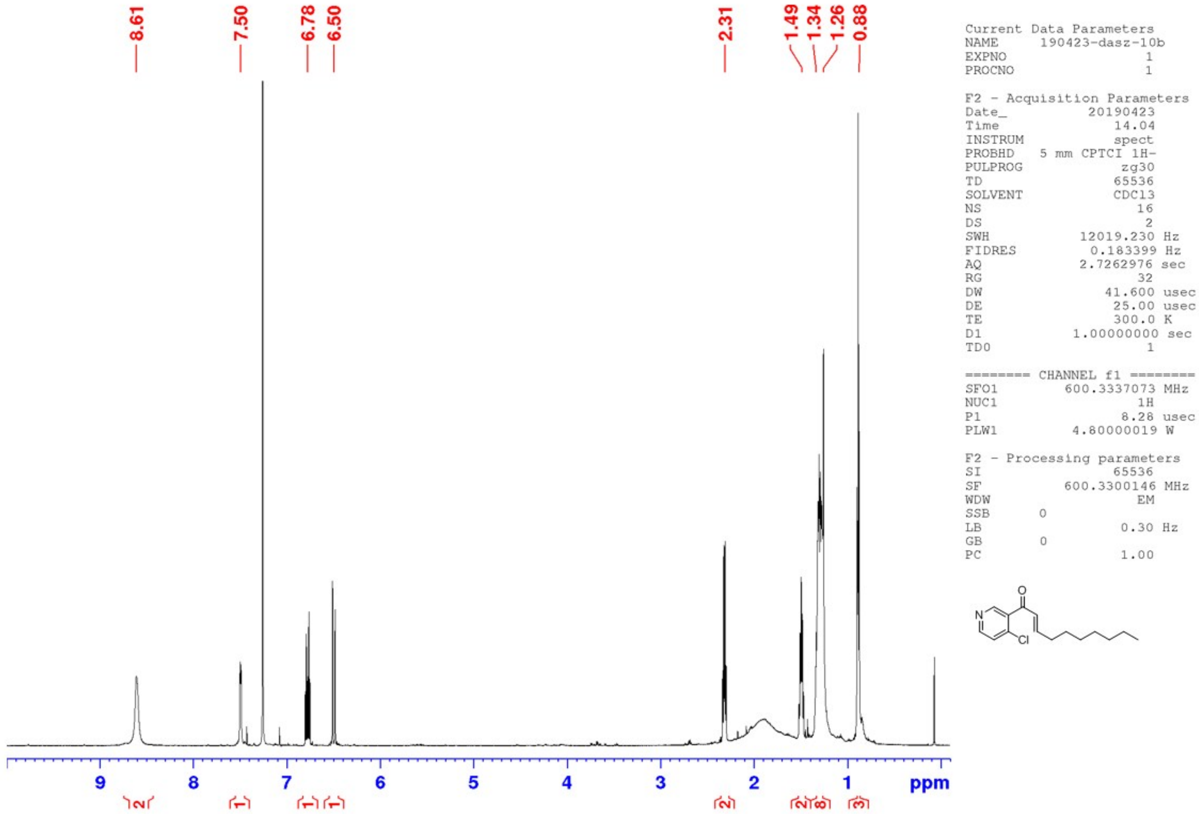

^{1}H-NMR spectra of (**10b**) in $CDCl_3$

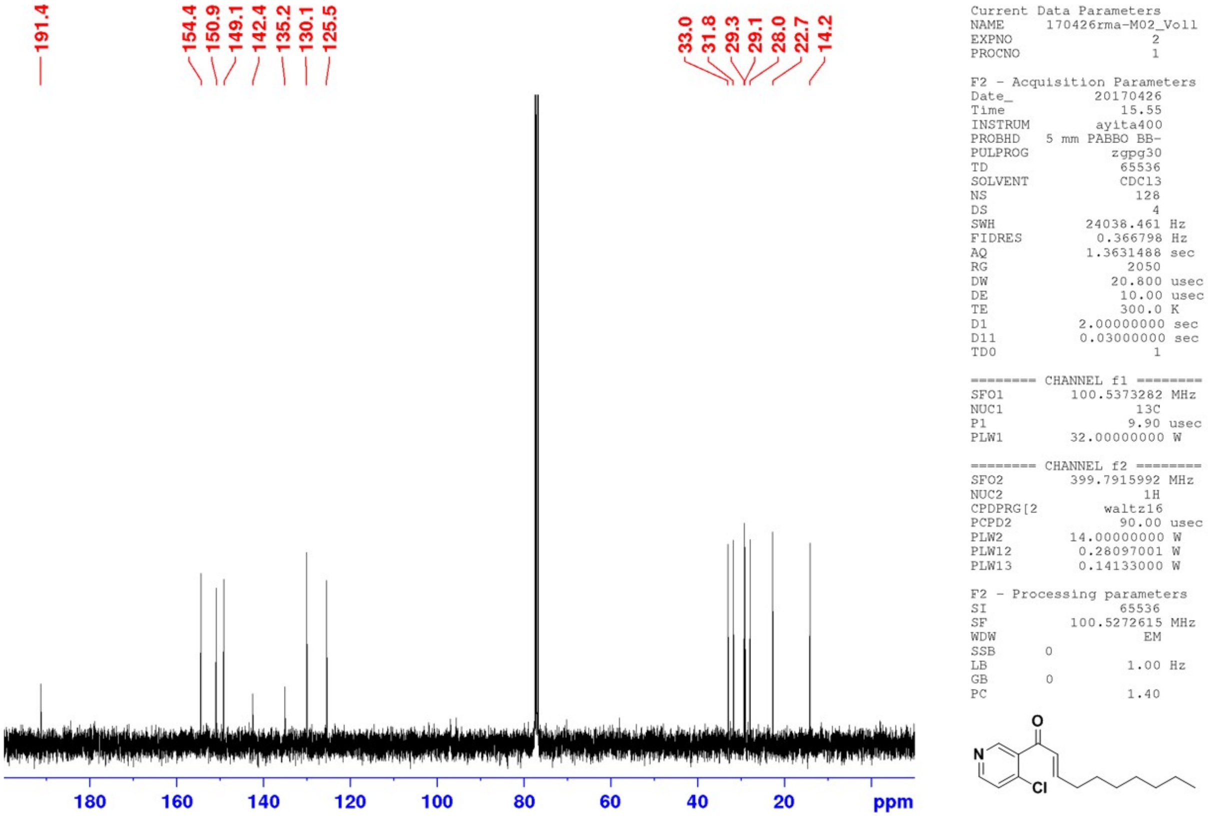

^{13}C-NMR spectra of (**10b**) in $CDCl_3$

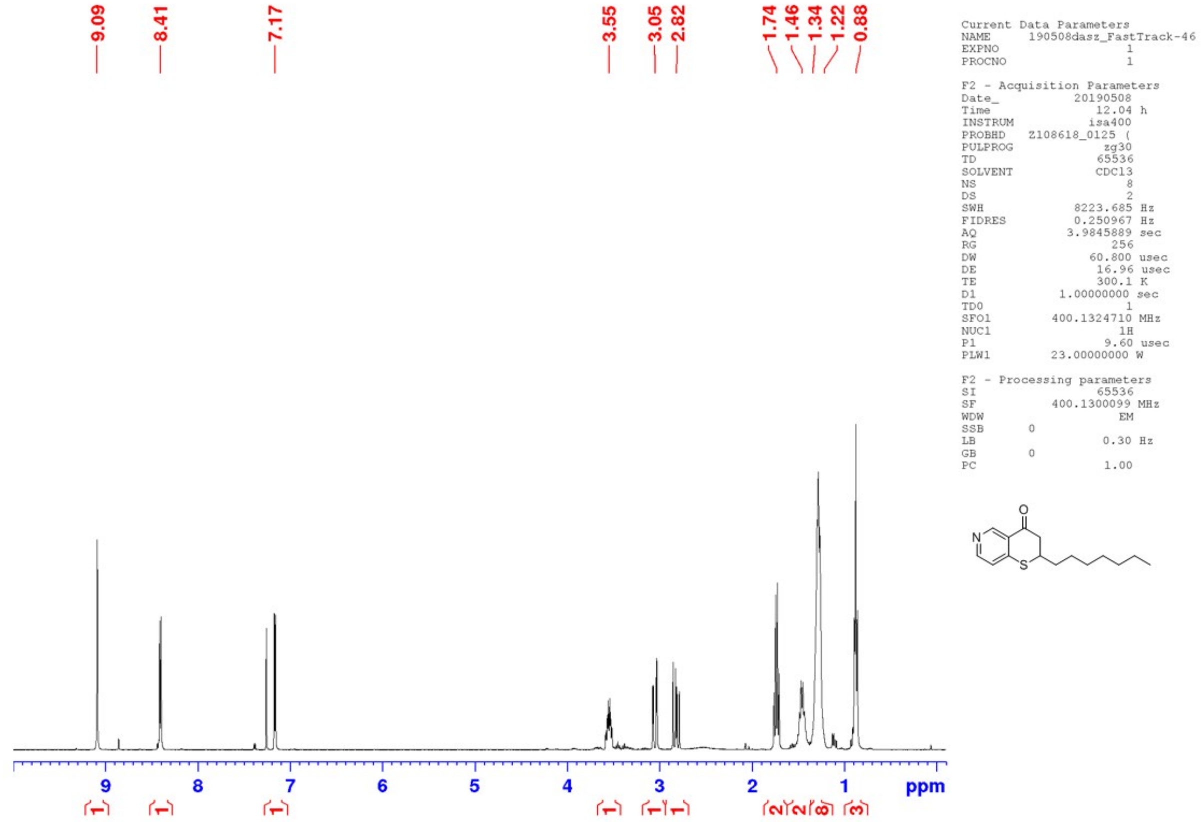

^{1}H-NMR spectra of (**10c**) in $CDCl_3$

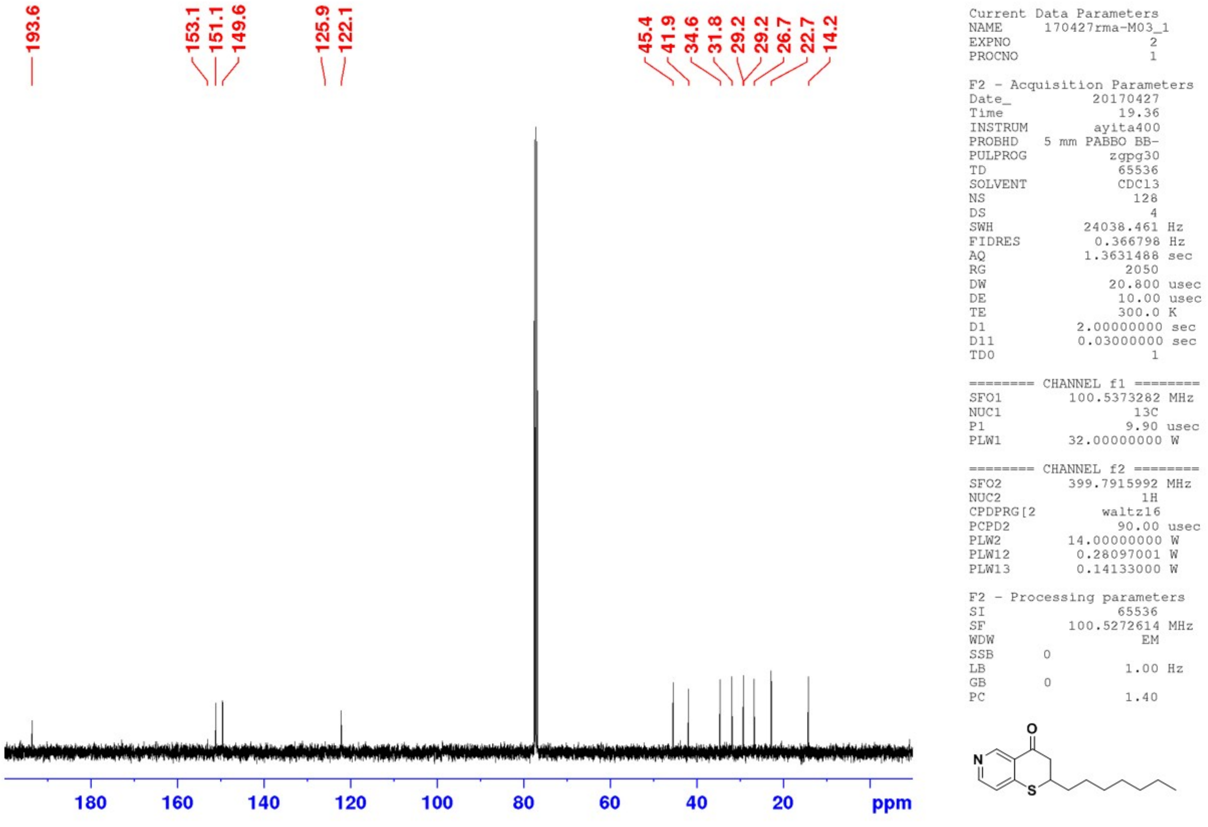

^{13}C-NMR spectra of (**10c**) in $CDCl_3$

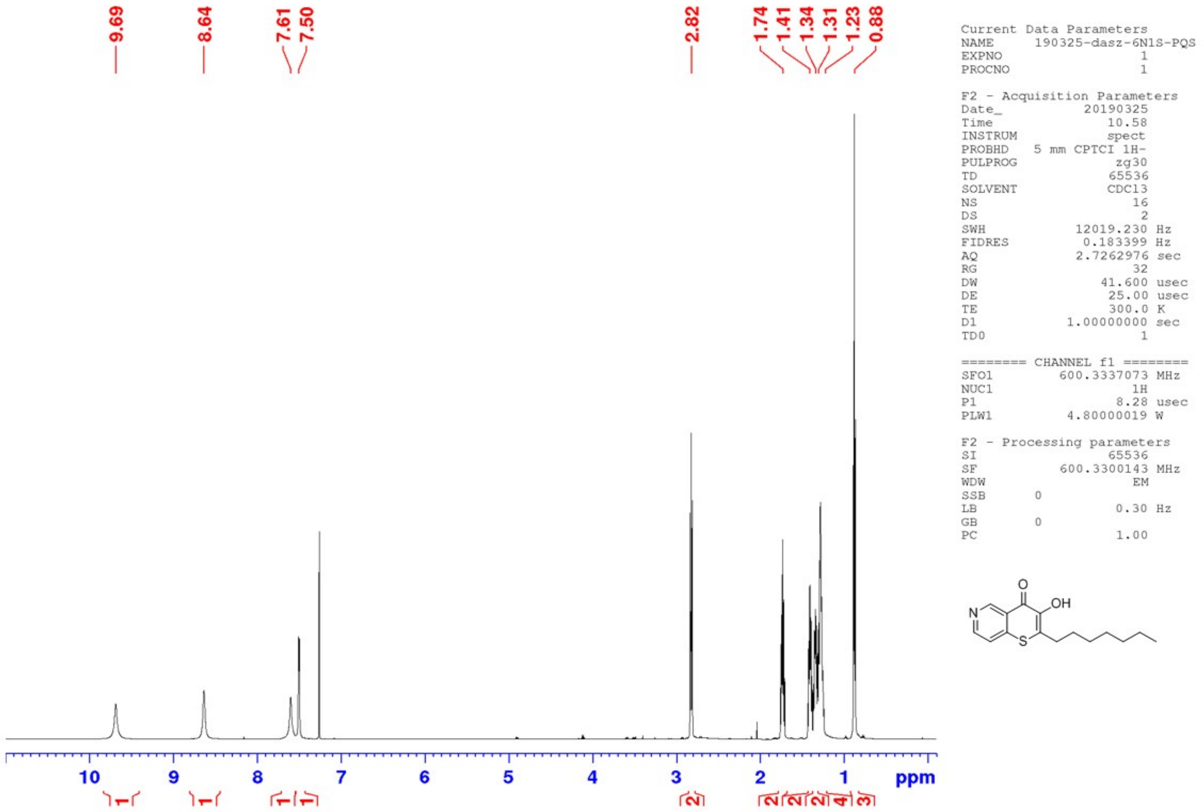

^{1}H-NMR spectra of (**10**) in $CDCl_3$

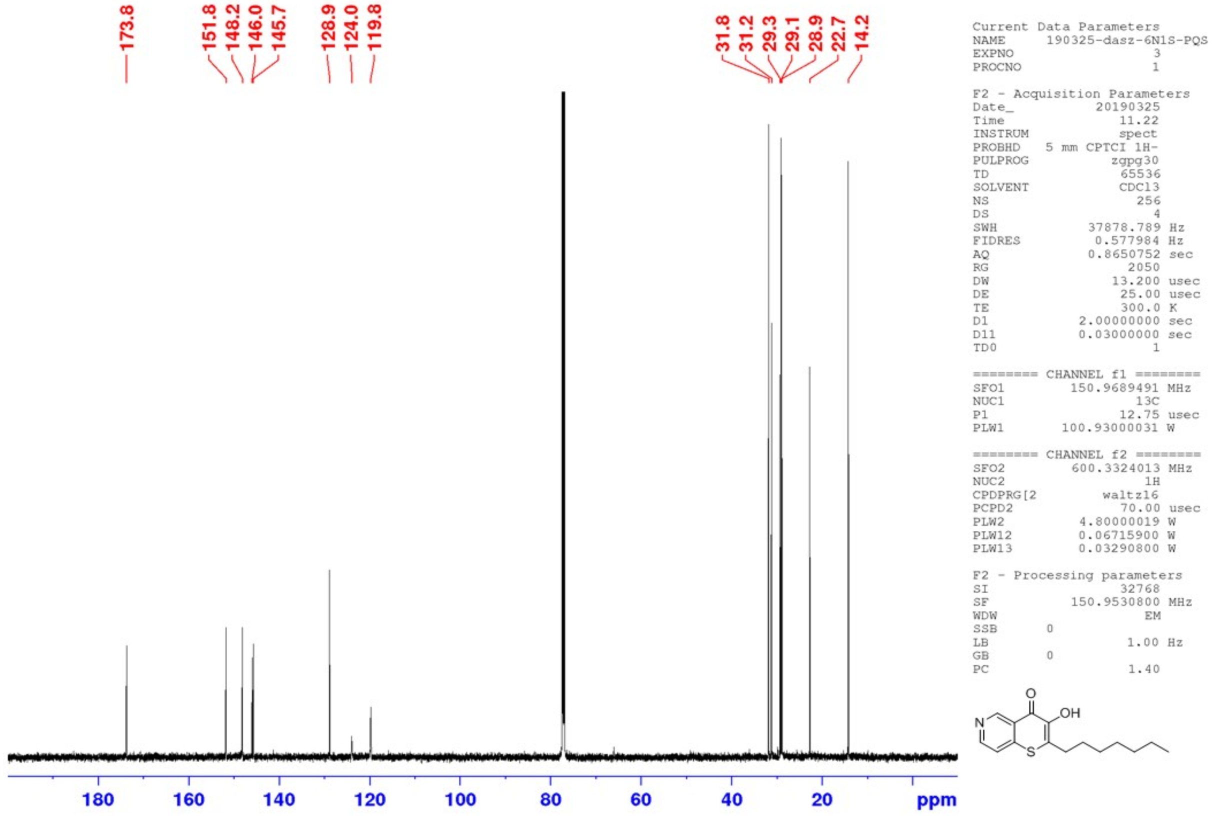

^{13}C-NMR spectra of (**10**) in $CDCl_3$

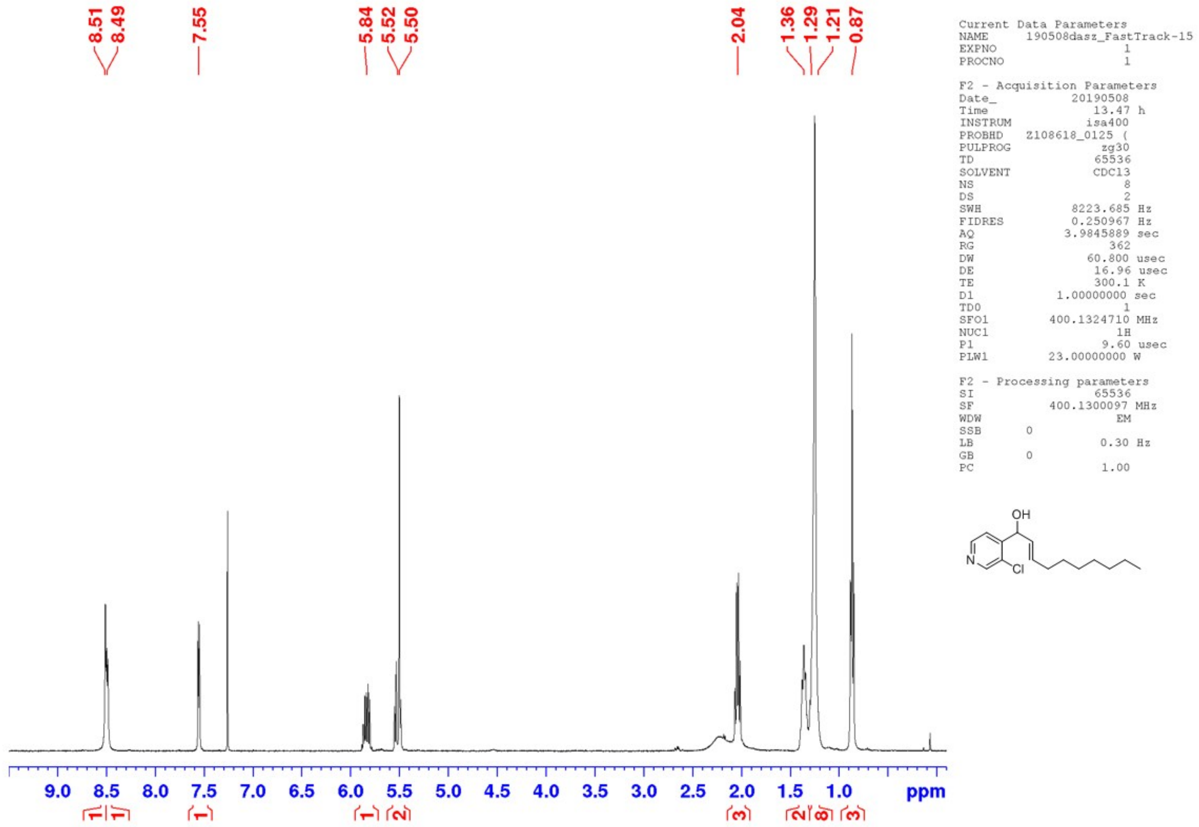

^{1}H-NMR spectra of (**11a**) in $CDCl_3$

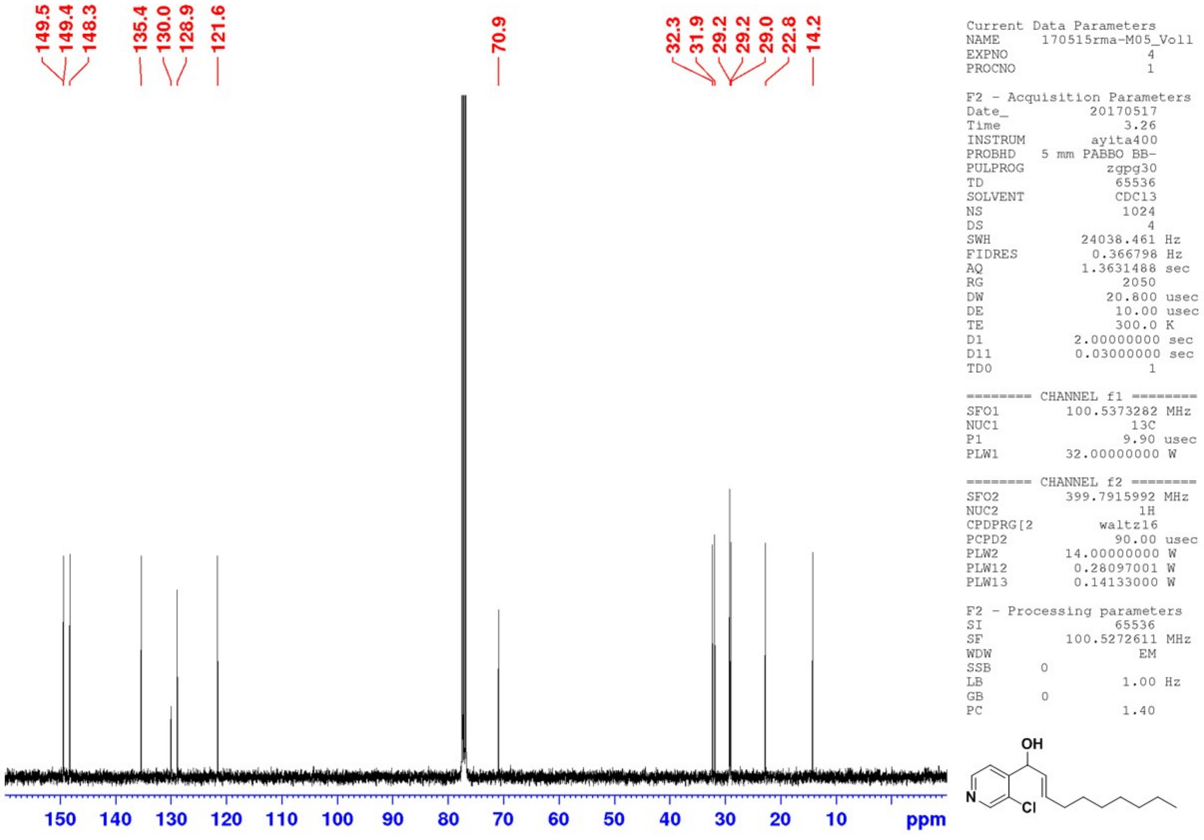

^{13}C-NMR spectra of (**11a**) in $CDCl_3$

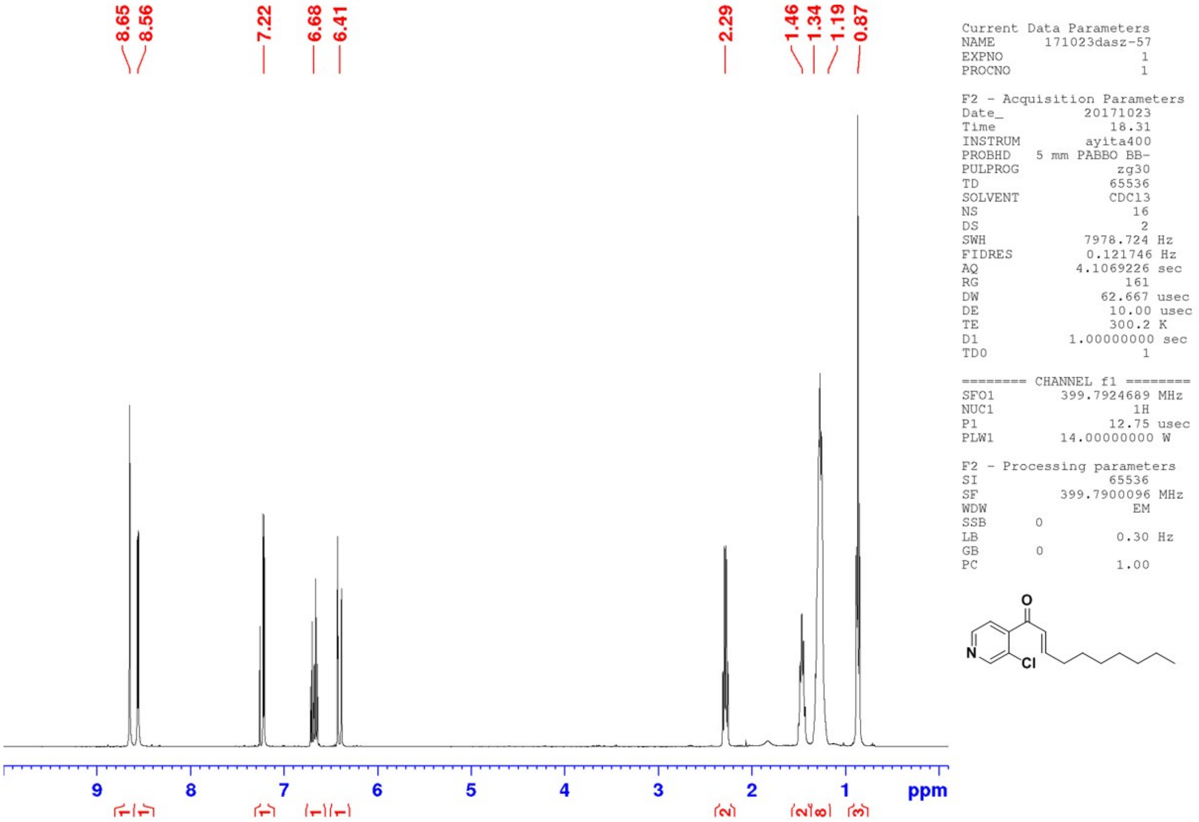

^{1}H-NMR spectra of (**11b**) in $CDCl_3$

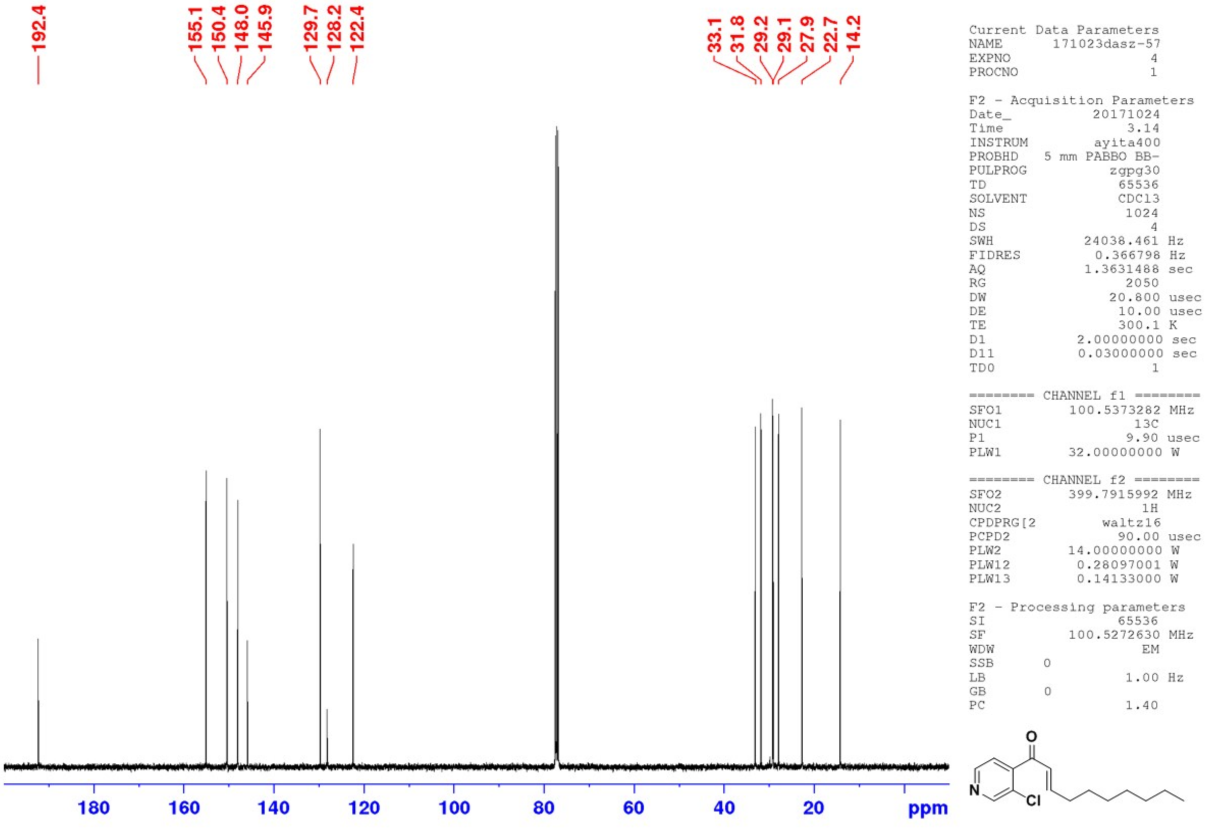

^{13}C-NMR spectra of (**11b**) in $CDCl_3$

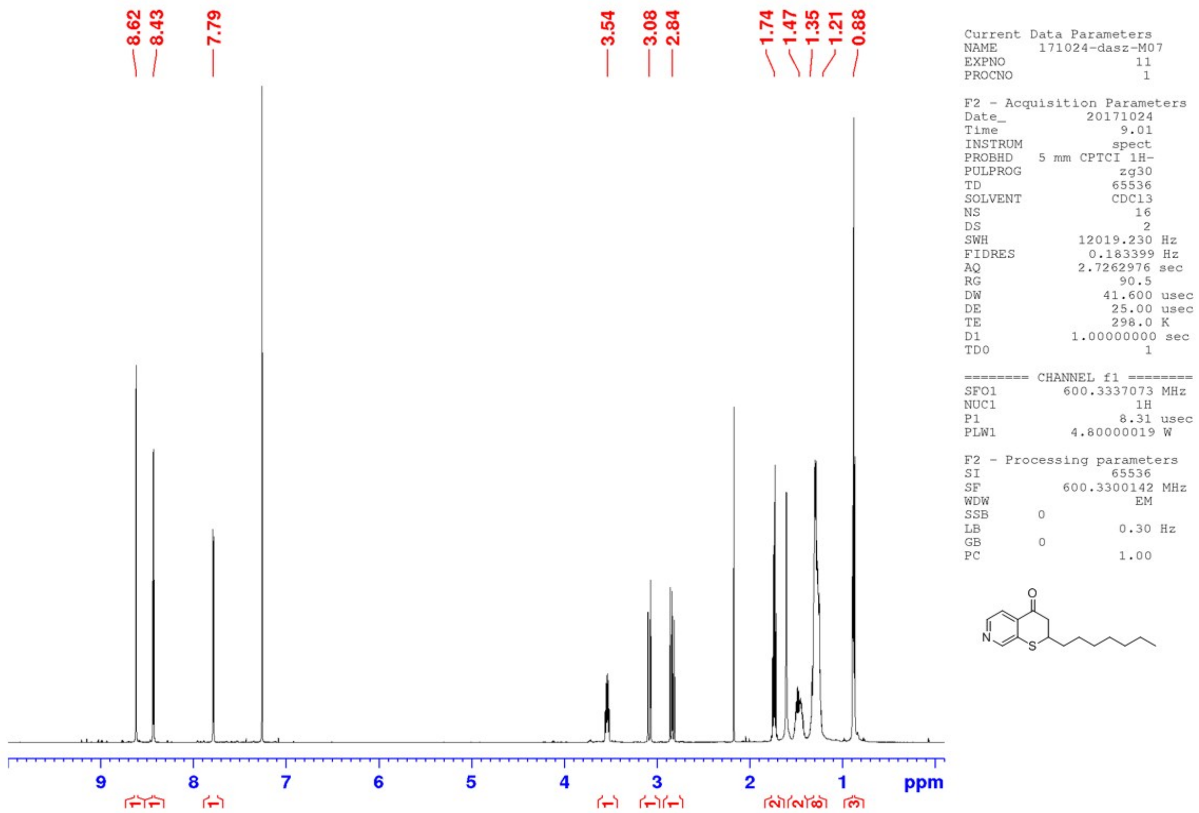

^{1}H-NMR spectra of (**11c**) in $CDCl_3$

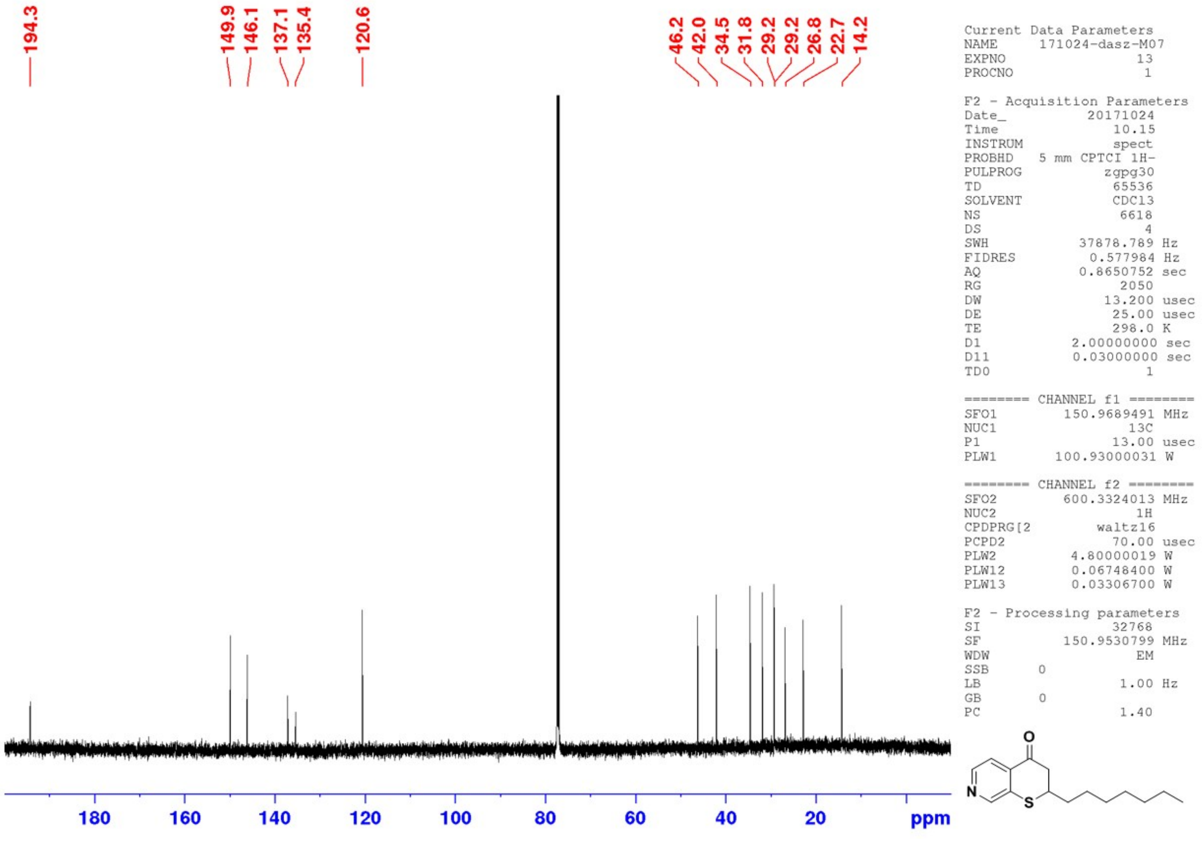

^{13}C-NMR spectra of (**11c**) in $CDCl_3$

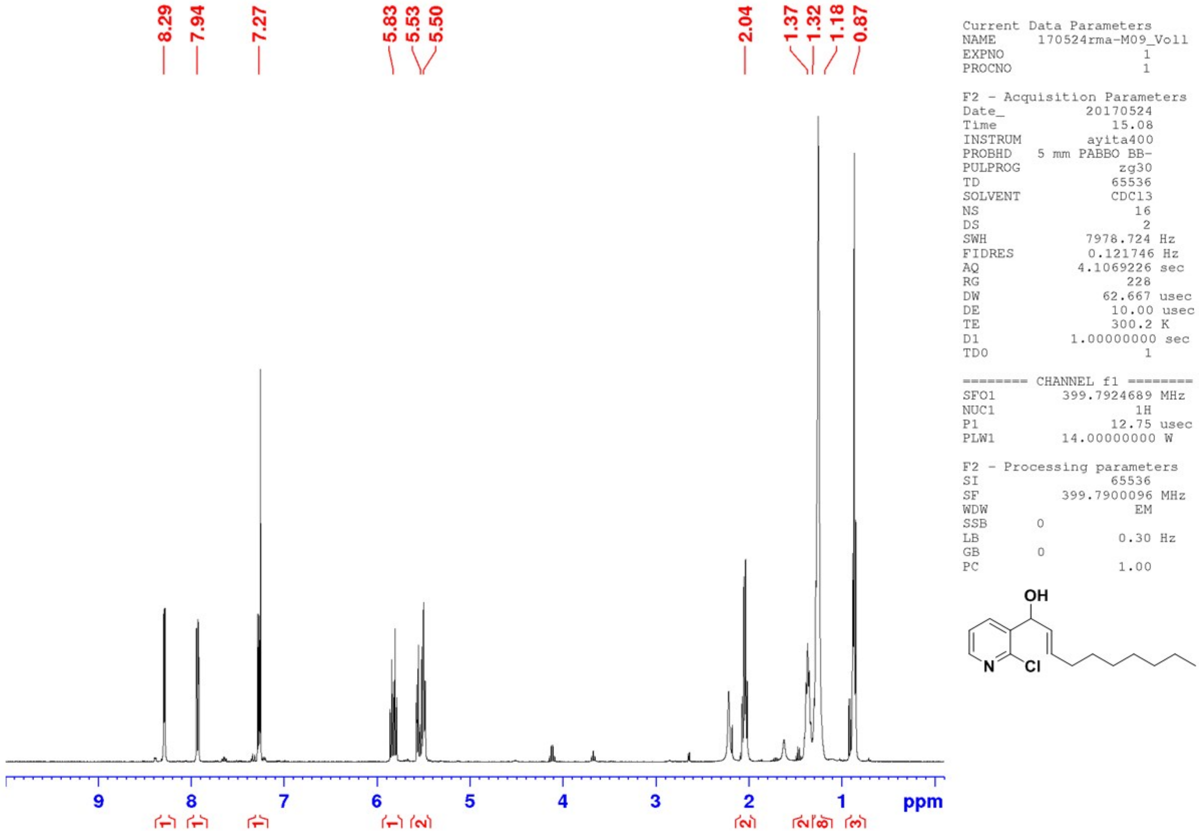

^{1}H-NMR spectra of (**9a**) in $CDCl_3$

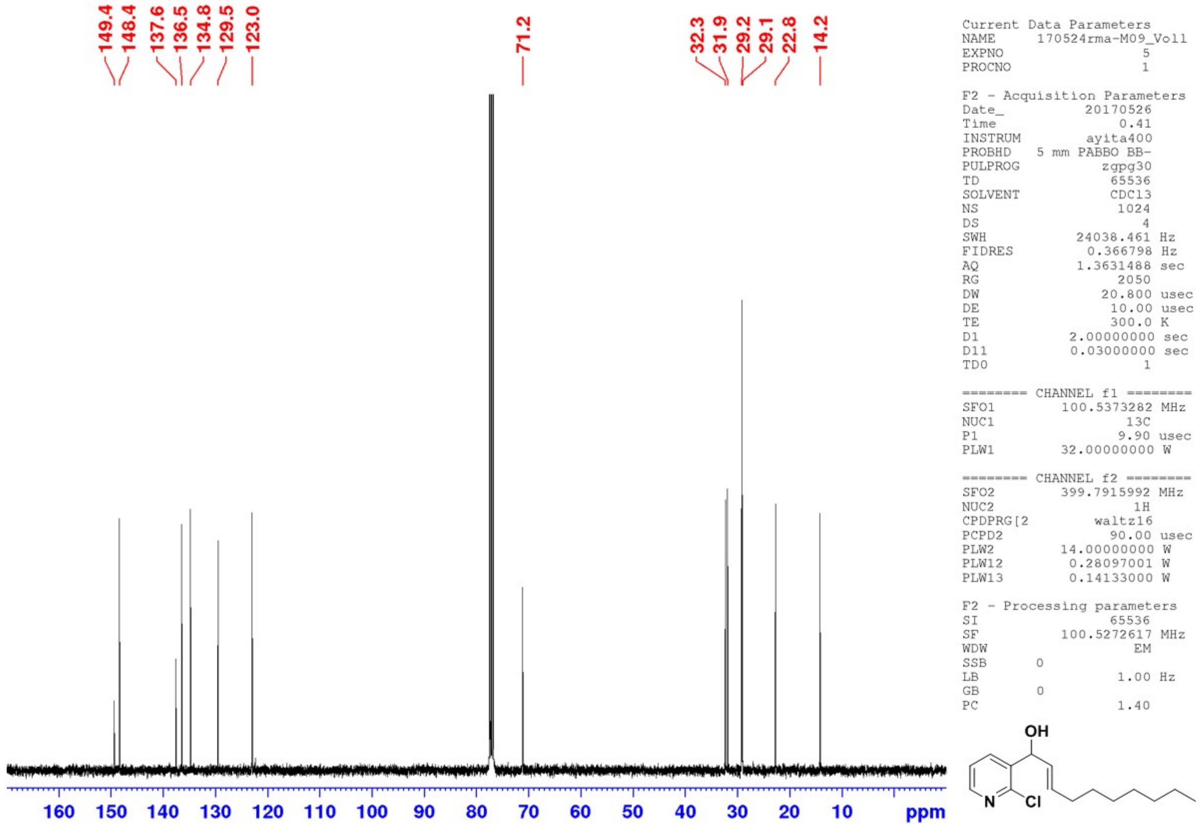

^{13}C-NMR spectra of (**9a**) in $CDCl_3$

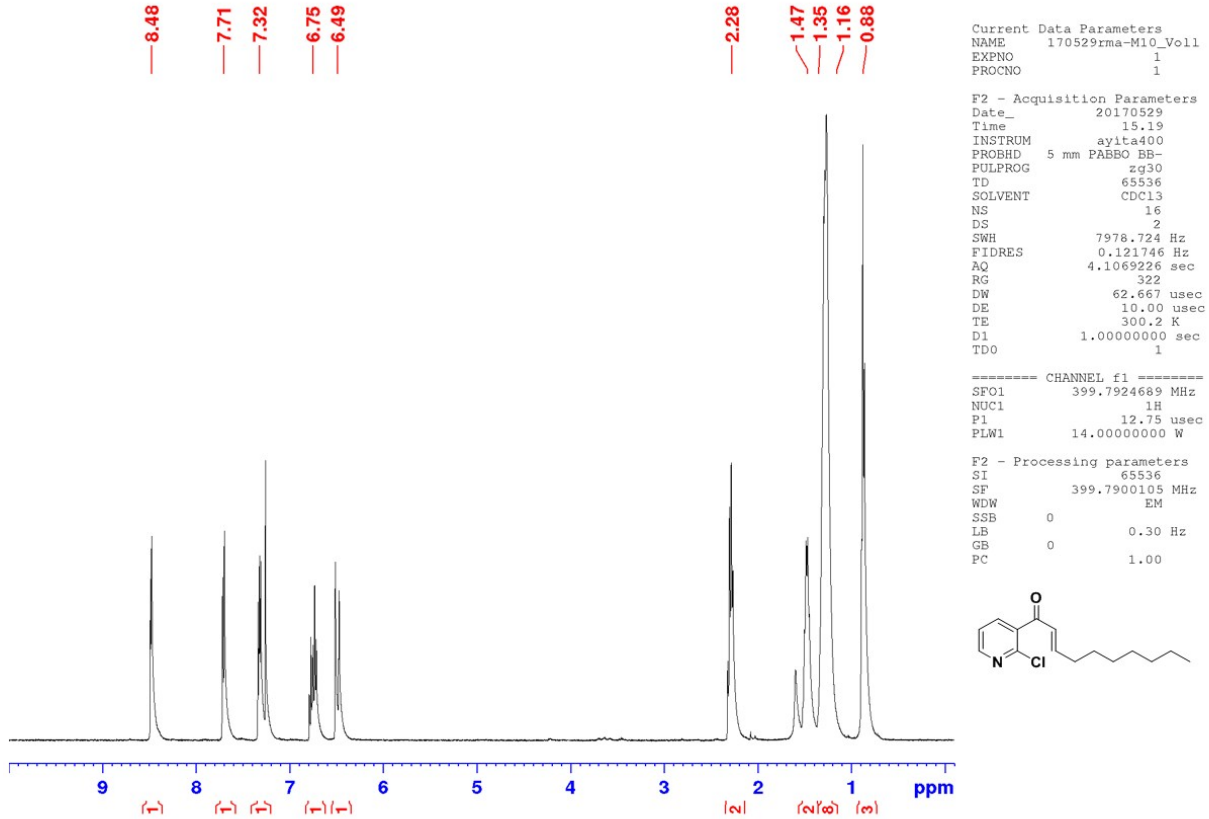

^{1}H-NMR spectra of (**9b**) in $CDCl_3$

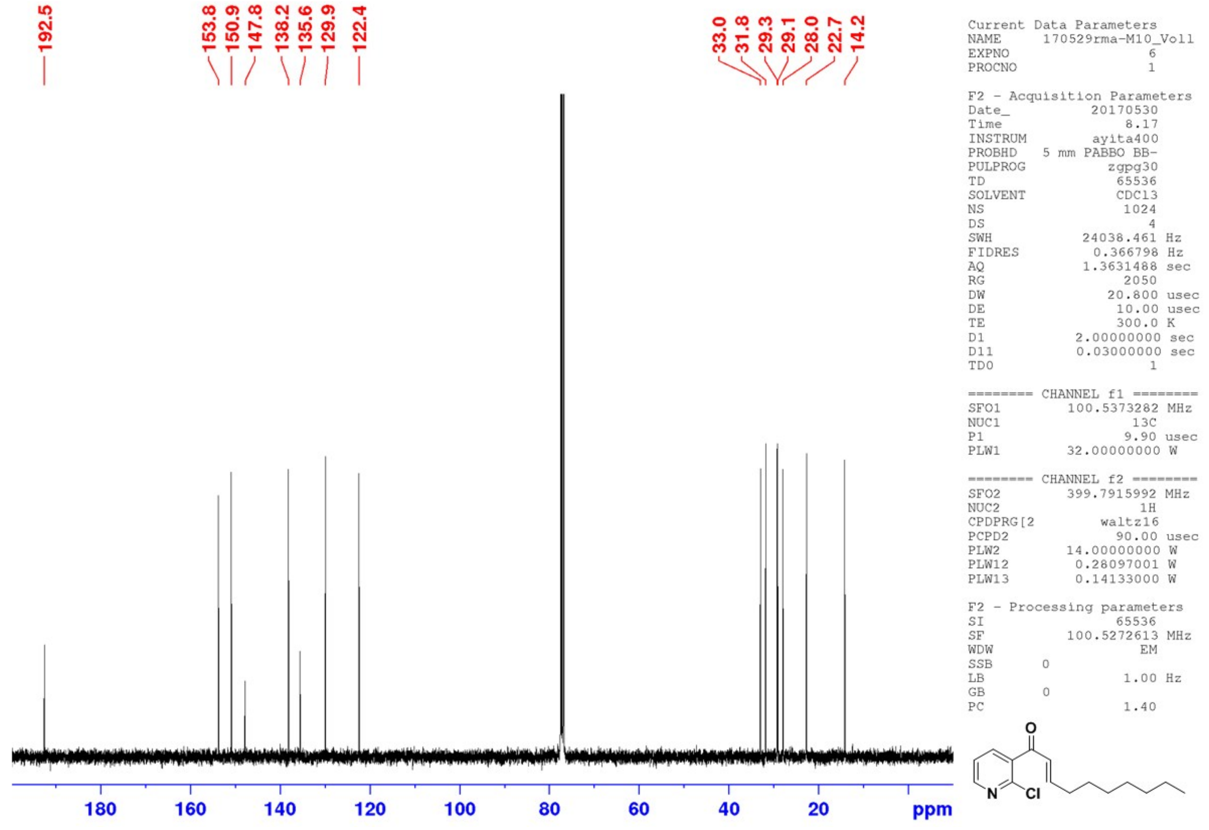

^{13}C-NMR spectra of (**9b**) in $CDCl_3$

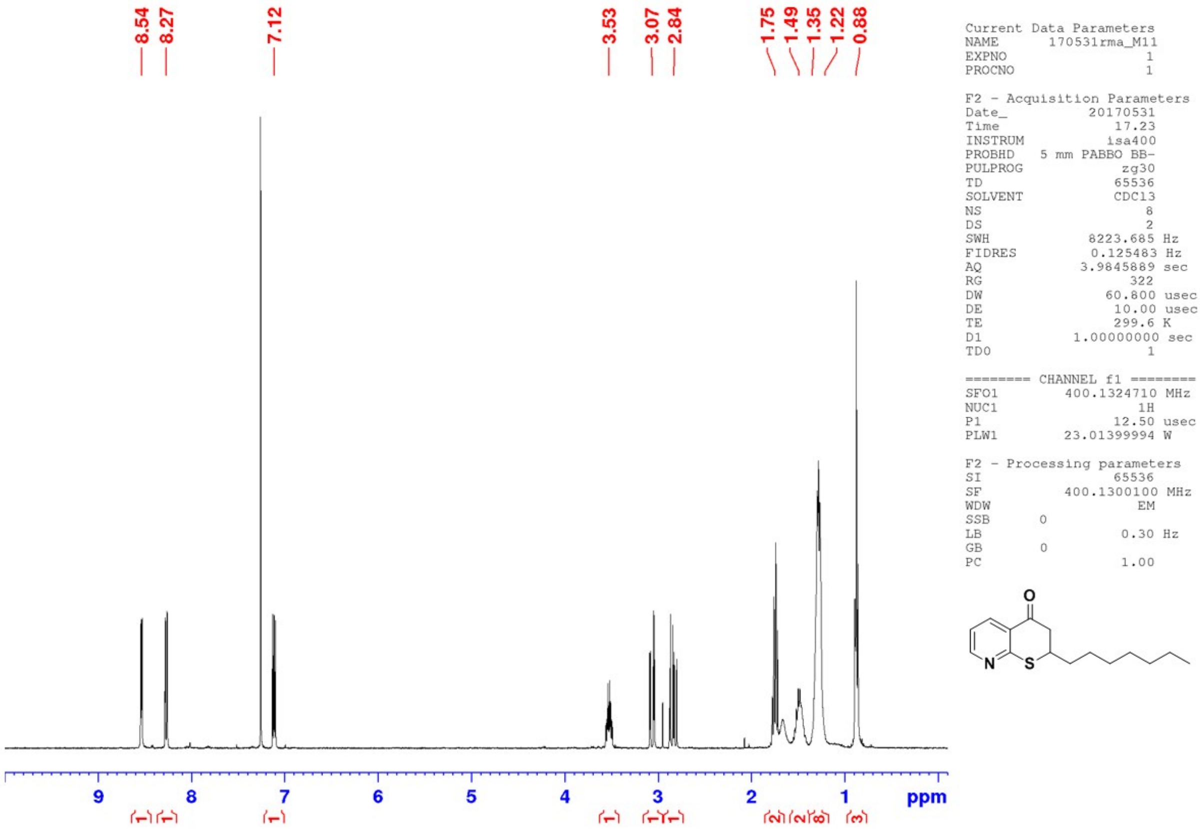

^{1}H-NMR spectra of (**9c**) in $CDCl_3$

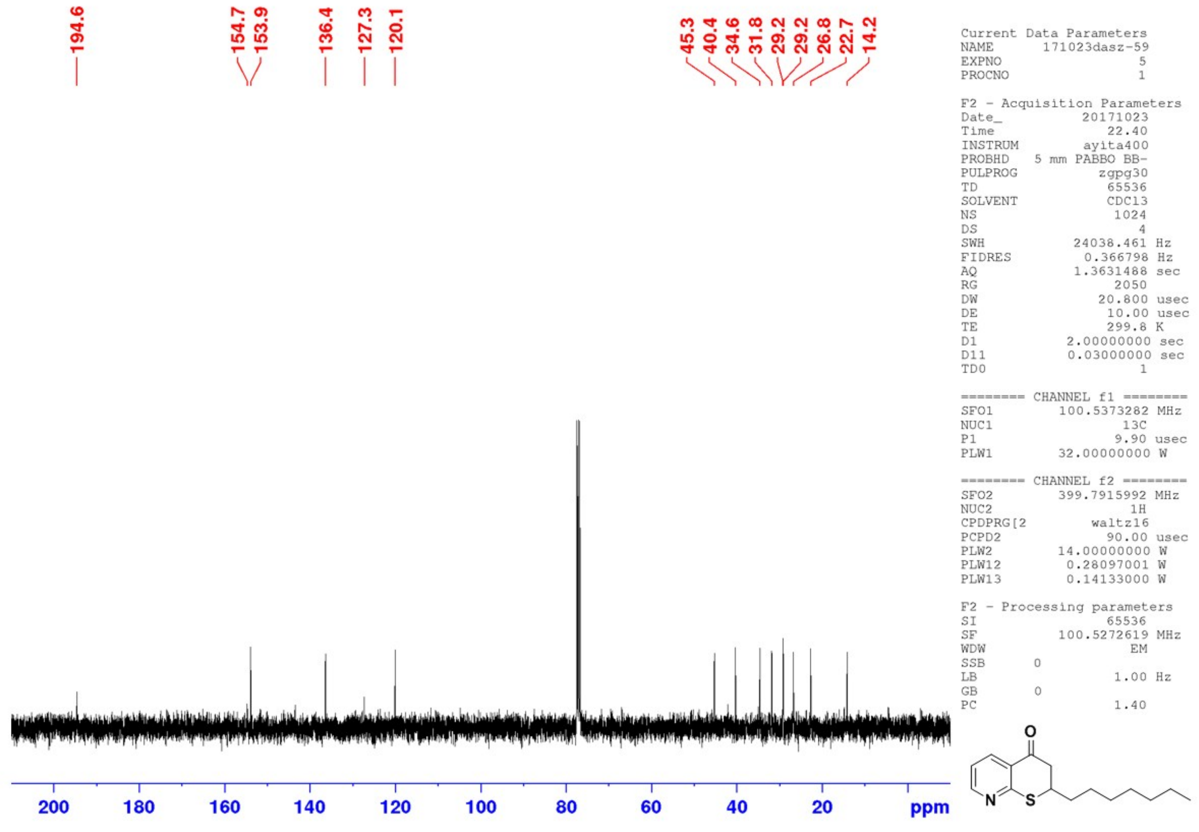

^{13}C-NMR spectra of (**9c**) in $CDCl_3$

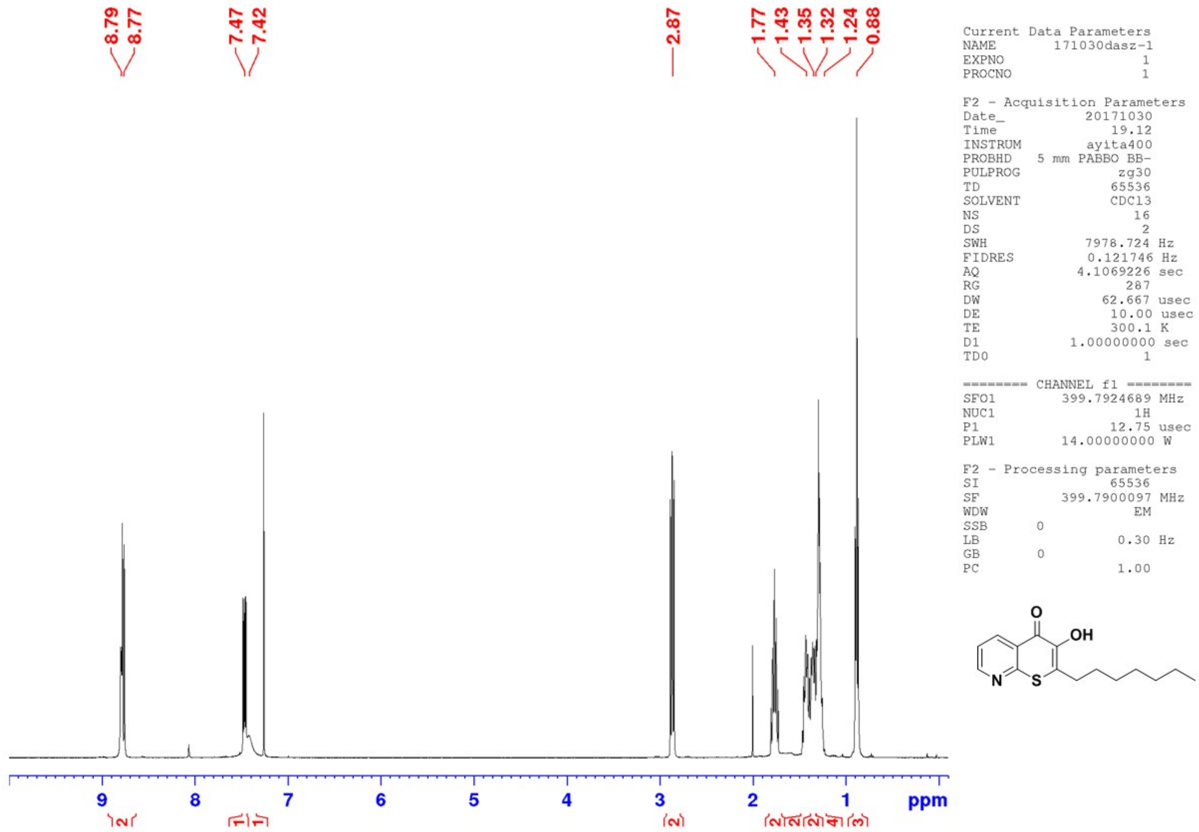

^{1}H-NMR spectra of (**9**) in $CDCl_3$

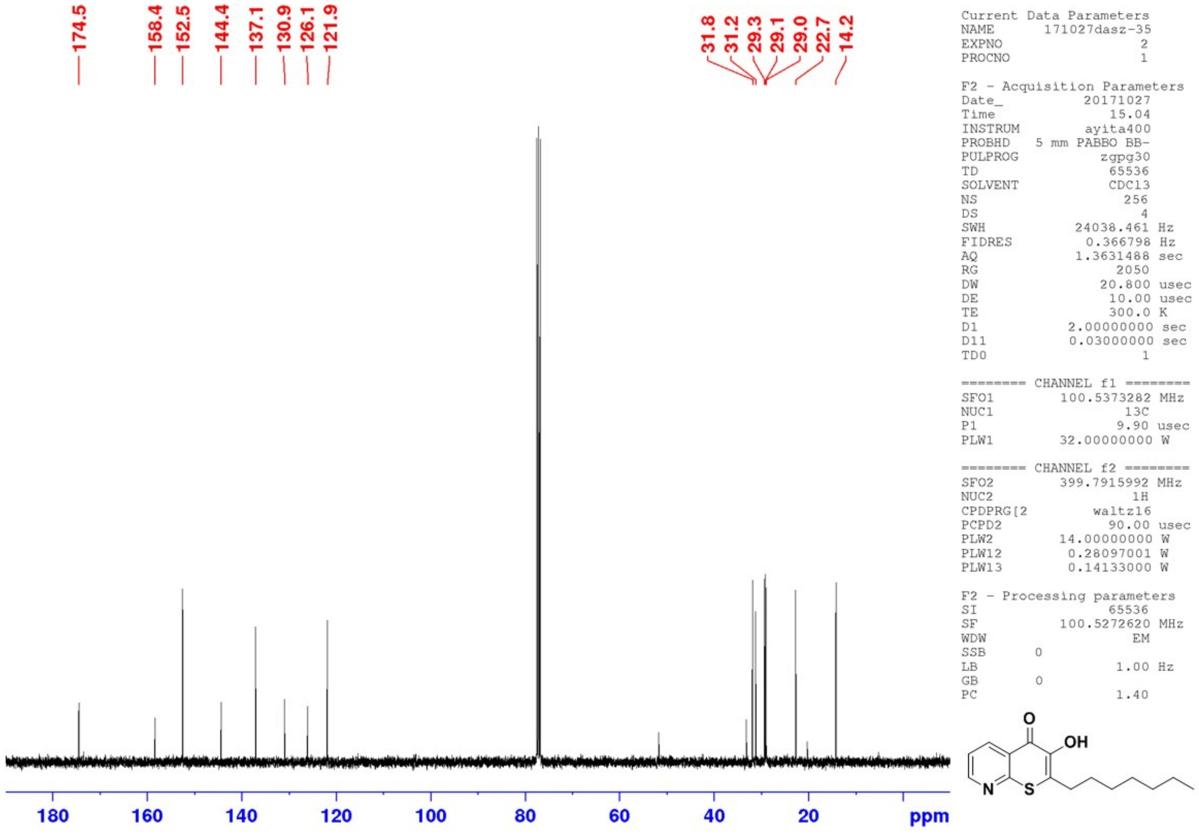

^{13}C-NMR spectra of (**9**) in $CDCl_3$

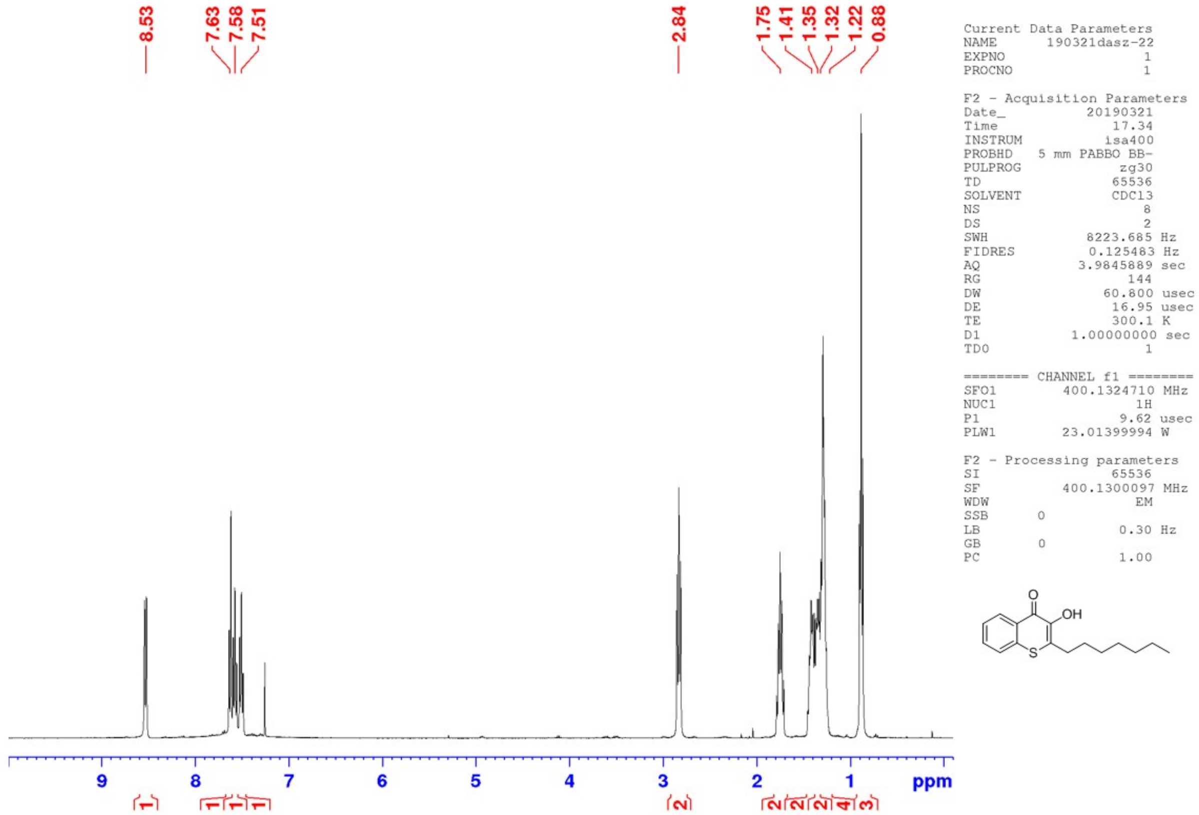

^{1}H-NMR spectra of (**3**) in $CDCl_3$

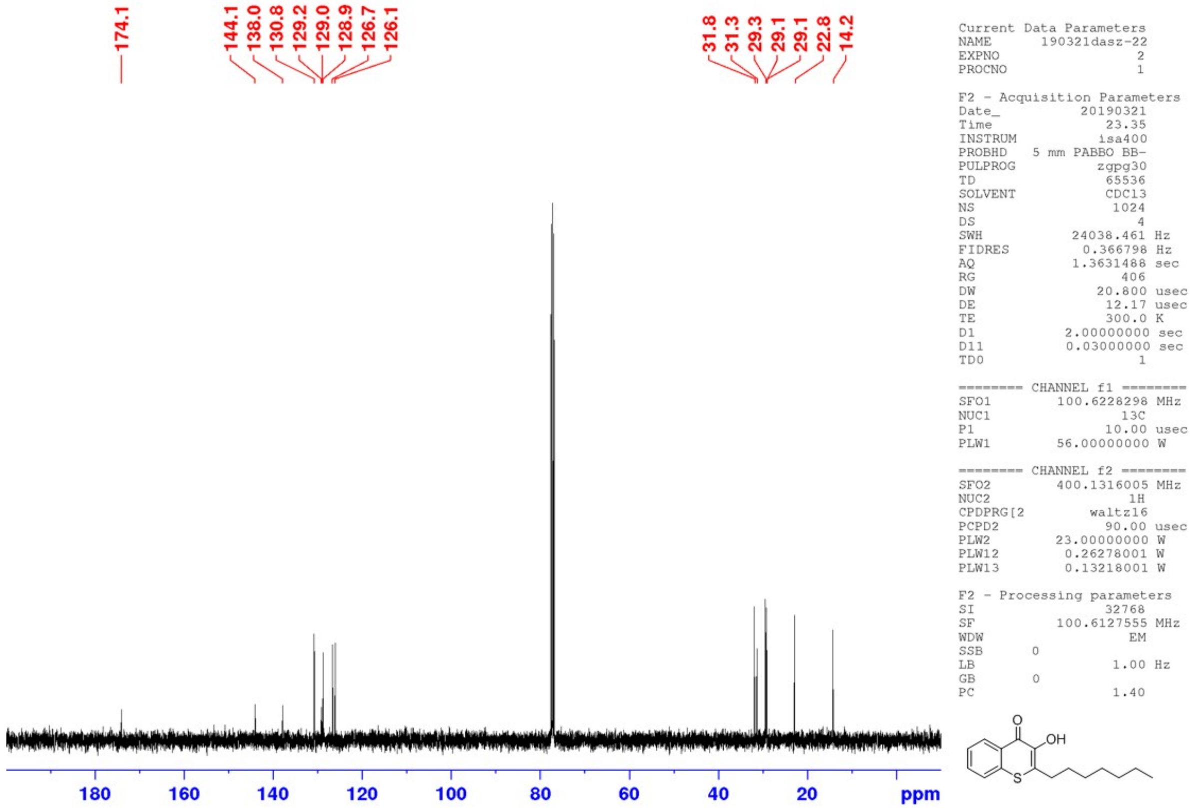

^{13}C-NMR spectra of (**3**) in $CDCl_3$

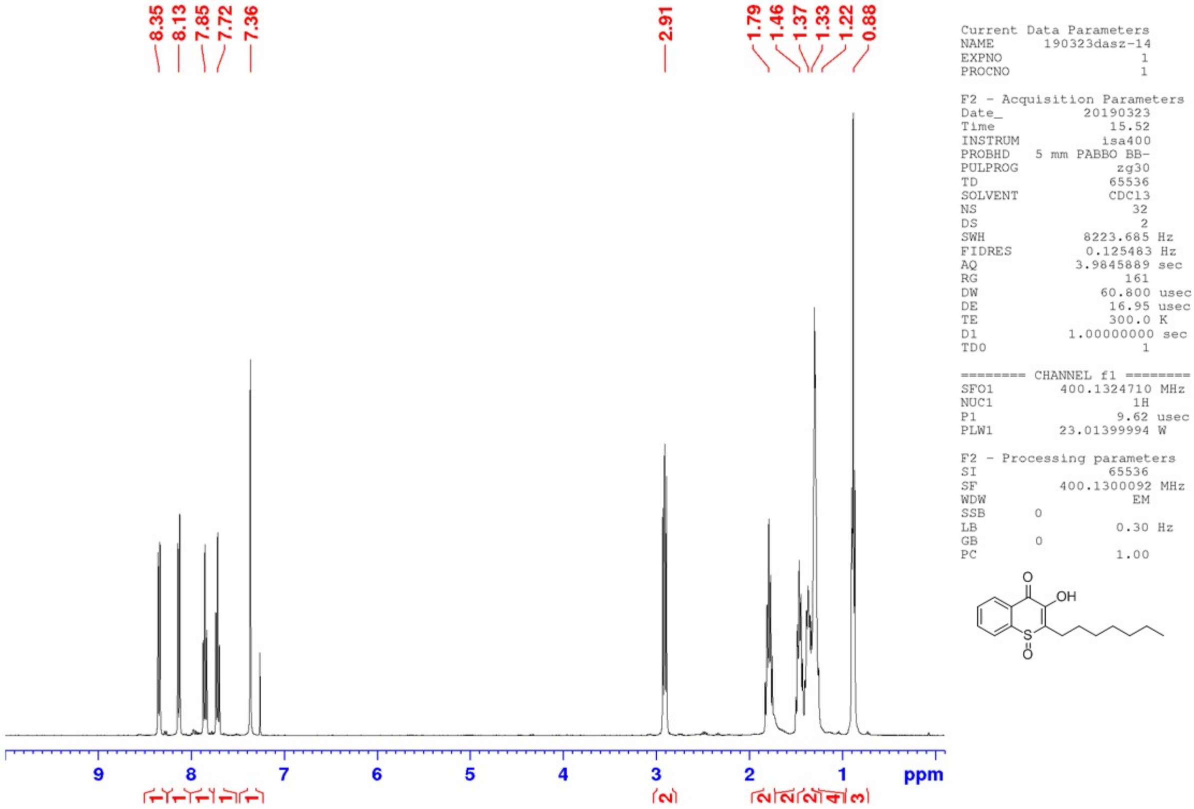

^{1}H-NMR spectra of (**5**) in $CDCl_3$

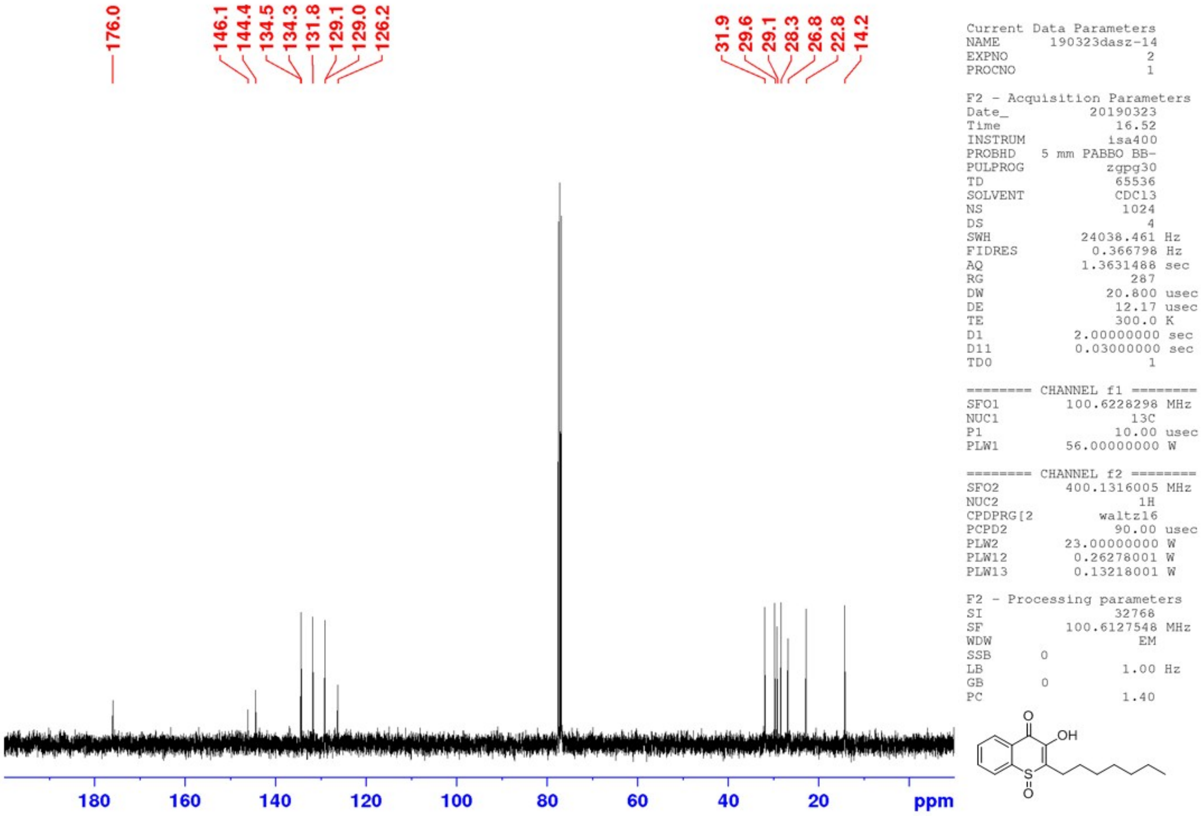

^{13}C-NMR spectra of (**5**) in $CDCl_3$

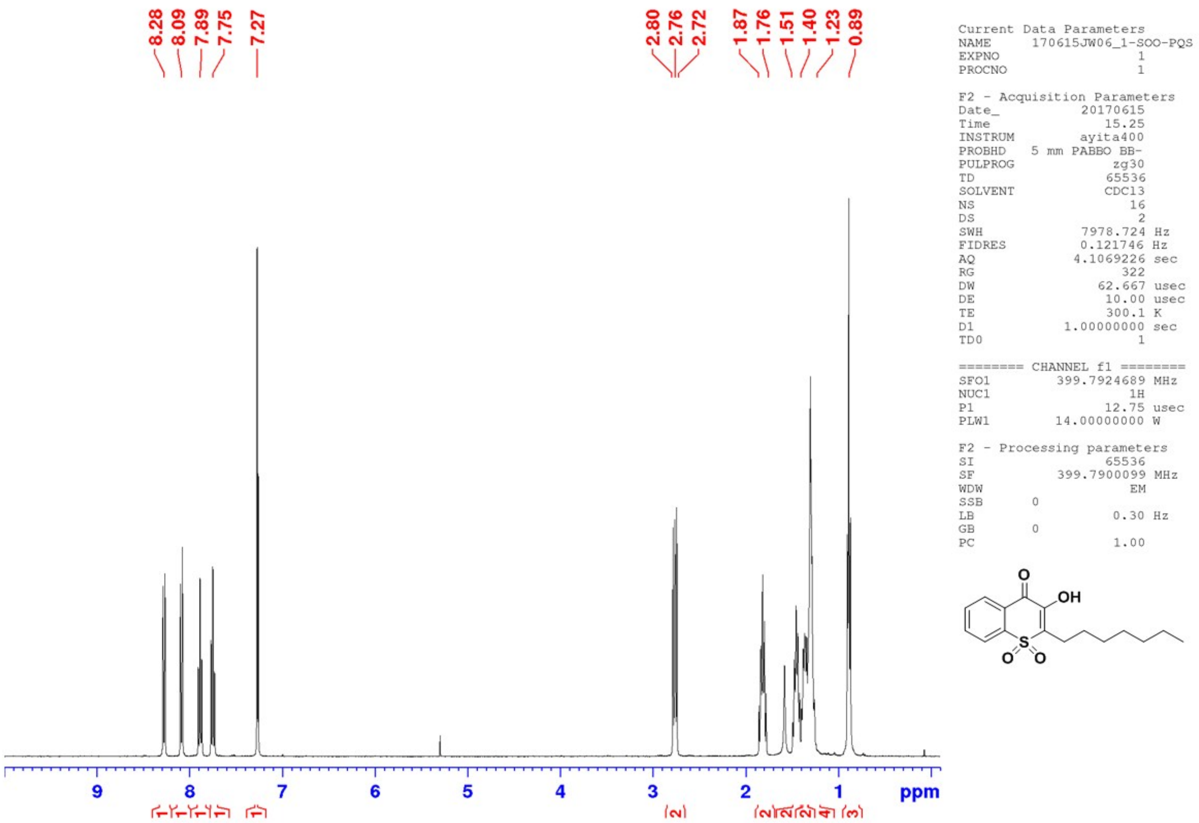

^{1}H-NMR spectra of (**6**) in $CDCl_3$

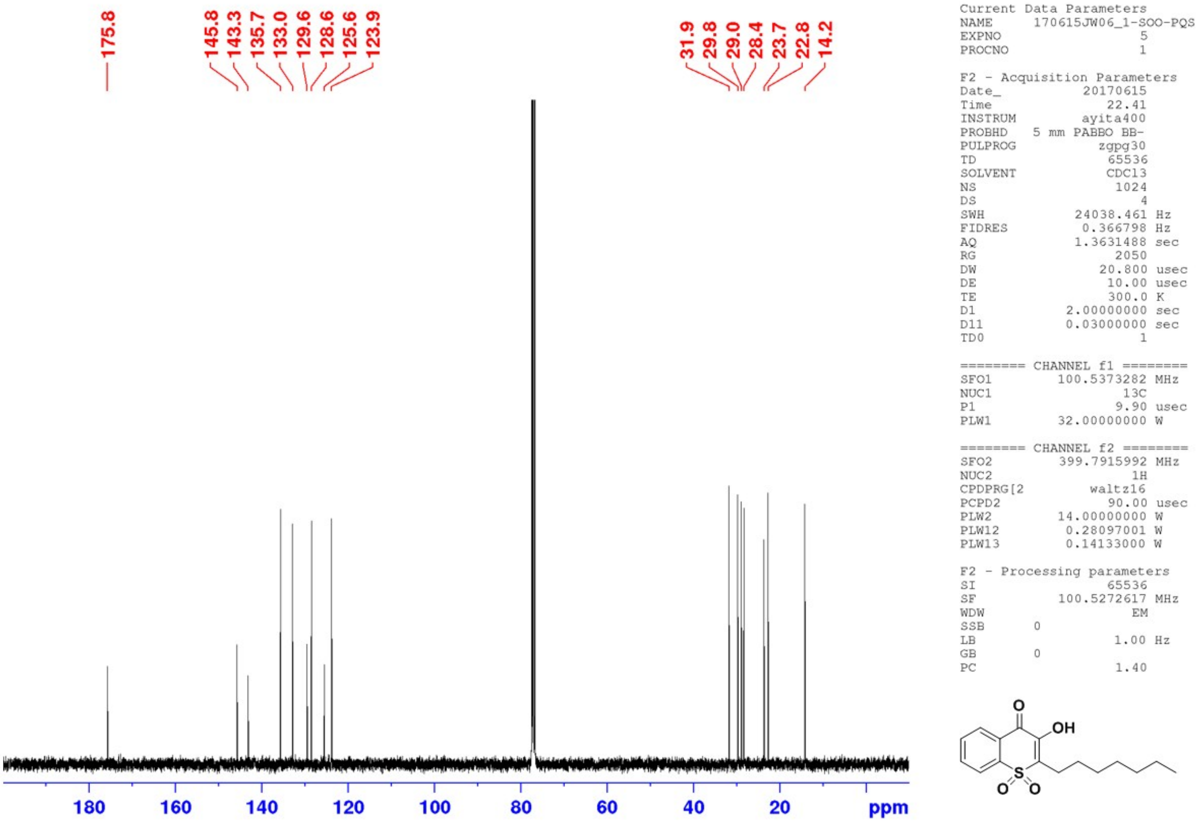

^{13}C-NMR spectra of (**6**) in $CDCl_3$

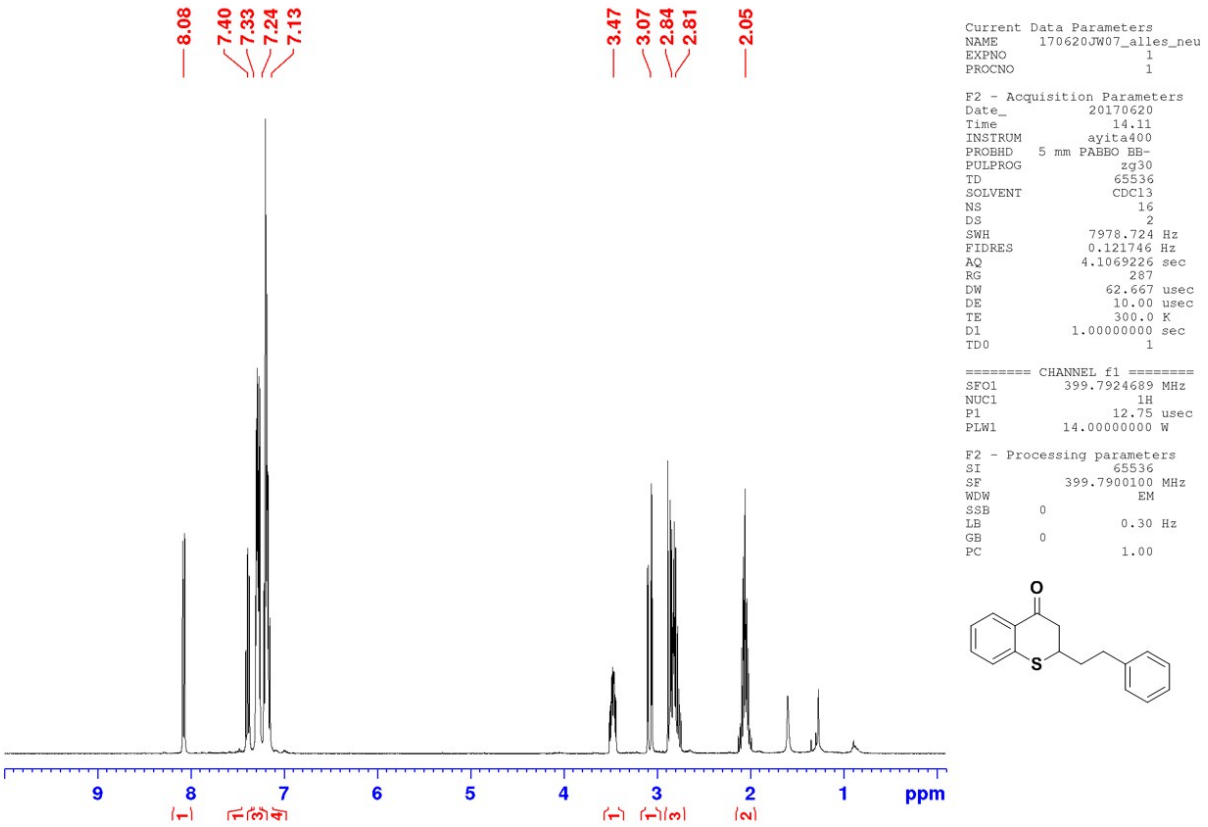

^{1}H-NMR spectra of (**7b**) in $CDCl_3$

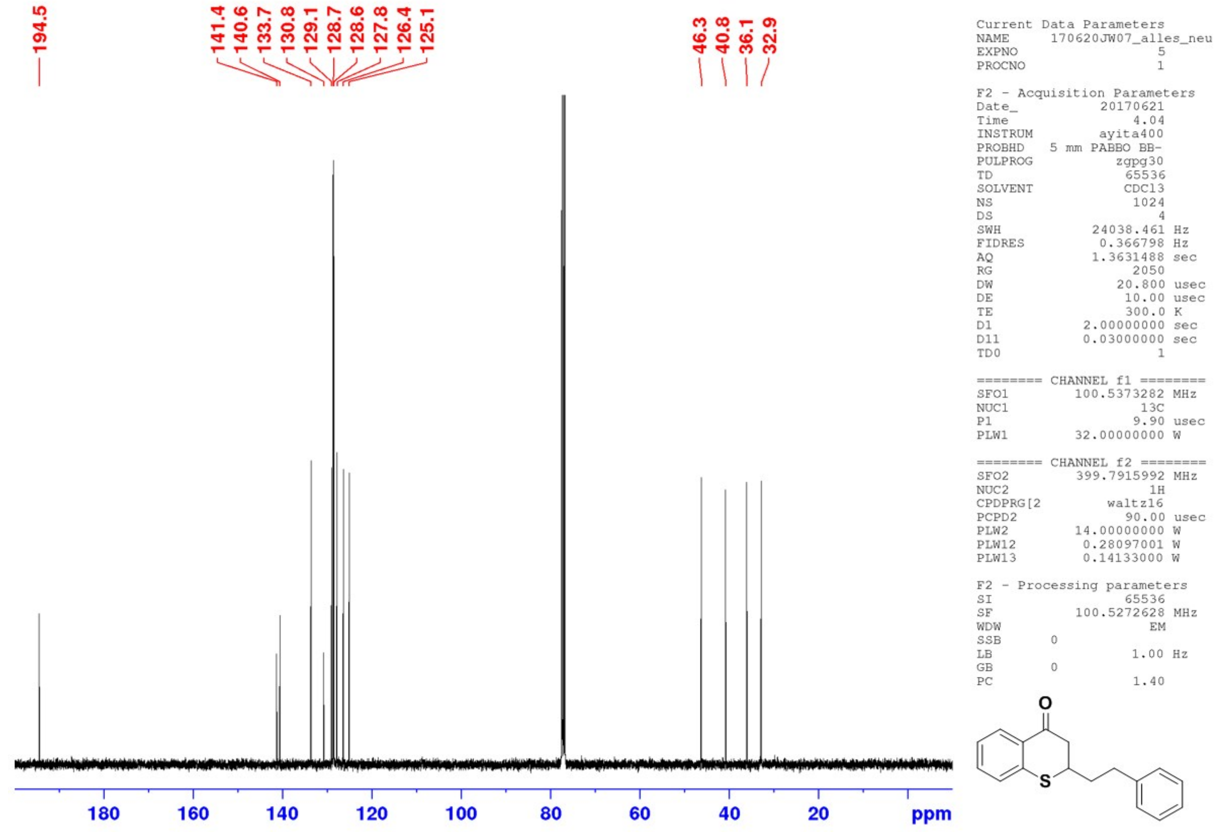

^{13}C-NMR spectra of (**7b**) in $CDCl_3$

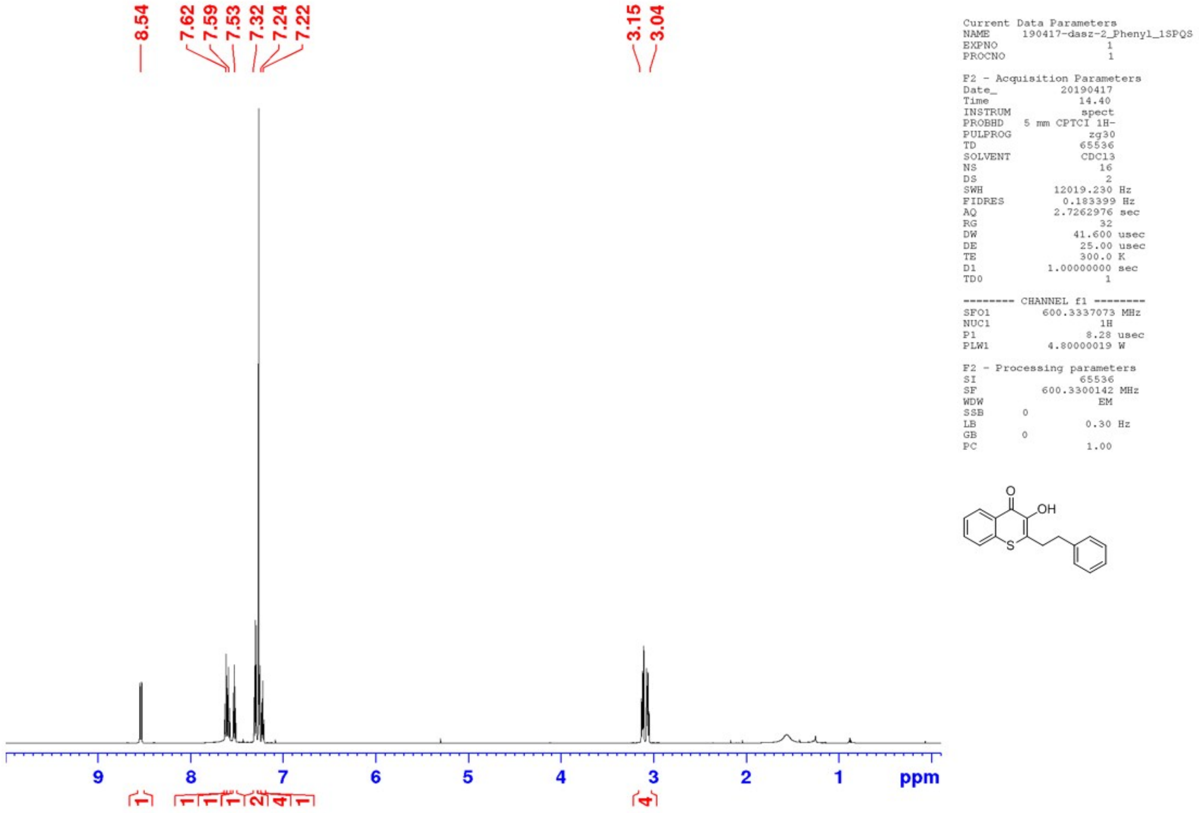

^{1}H-NMR spectra of (**7**) in $CDCl_3$

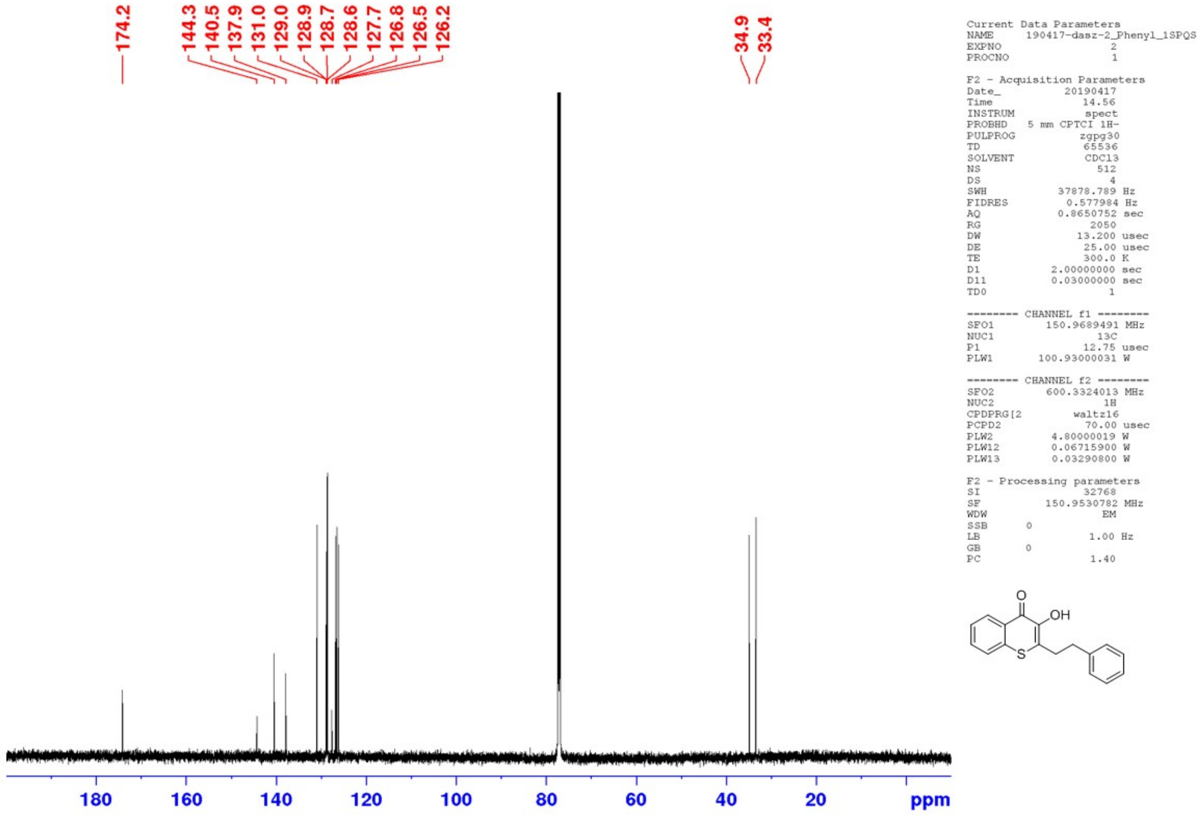

^{13}C-NMR spectra of (**7**) in $CDCl_3$

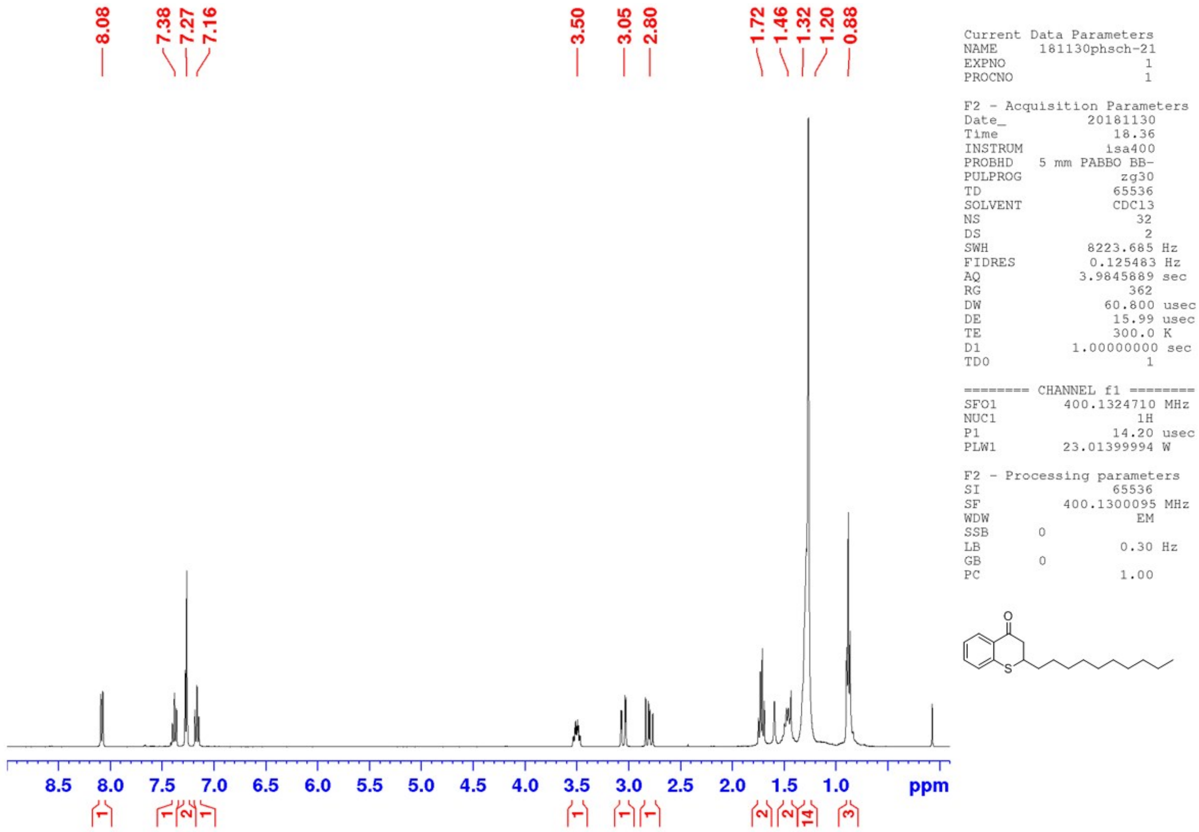

^{1}H-NMR spectra of (**8b**) in $CDCl_3$

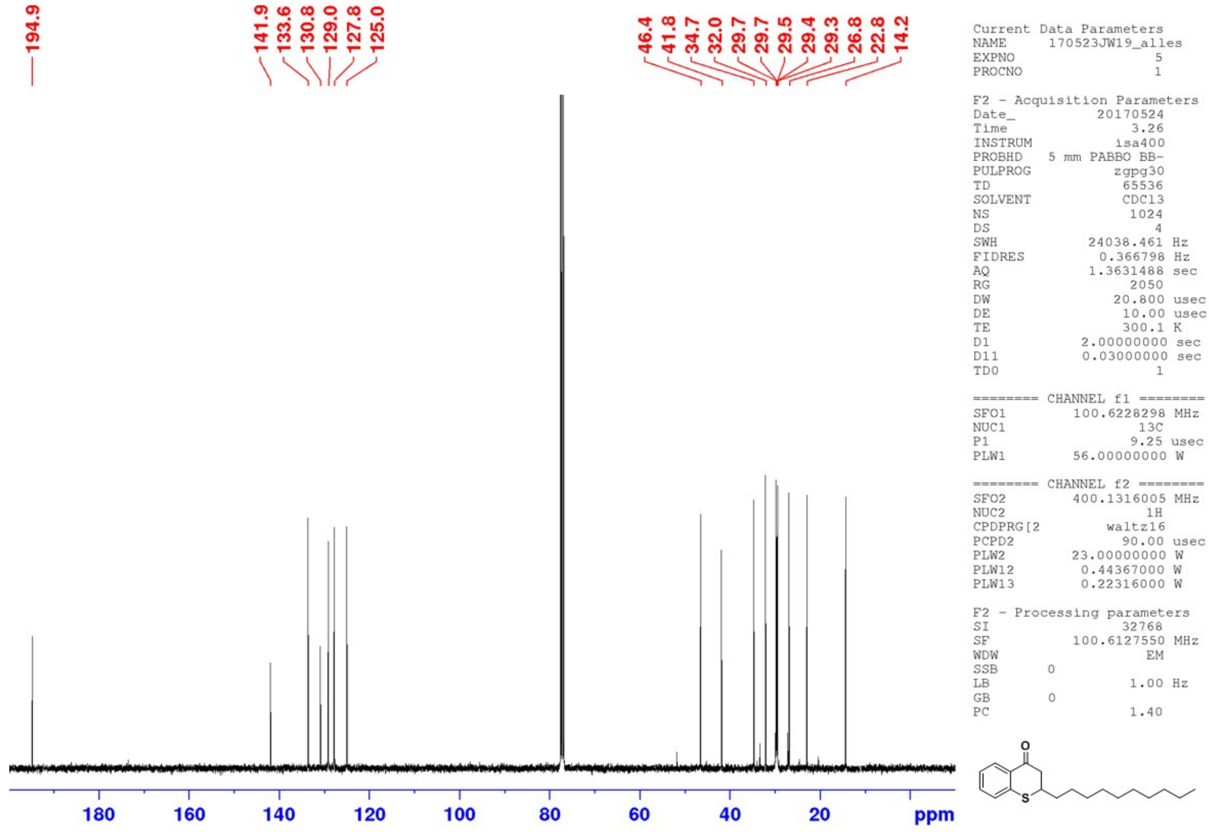

^{13}C-NMR spectra of (**8b**) in $CDCl_3$

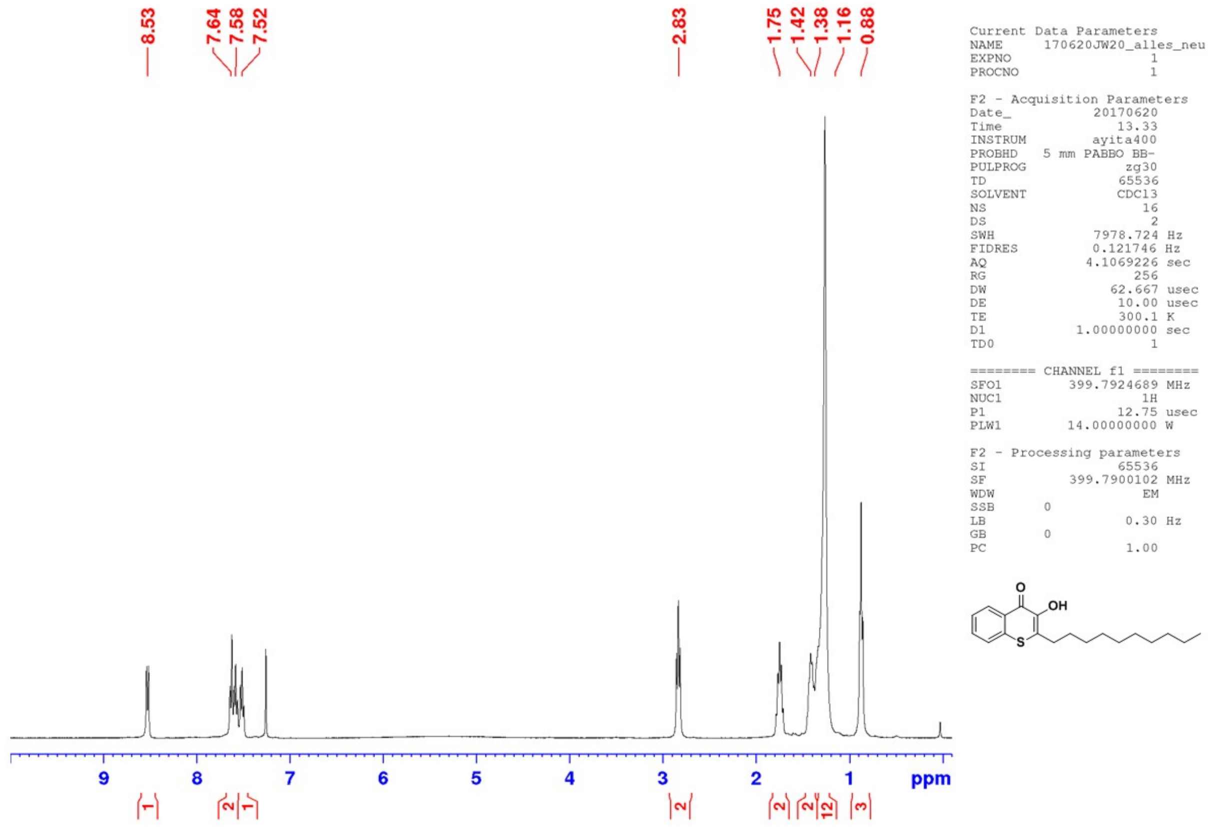

^{1}H-NMR spectra of (**8**) in $CDCl_3$

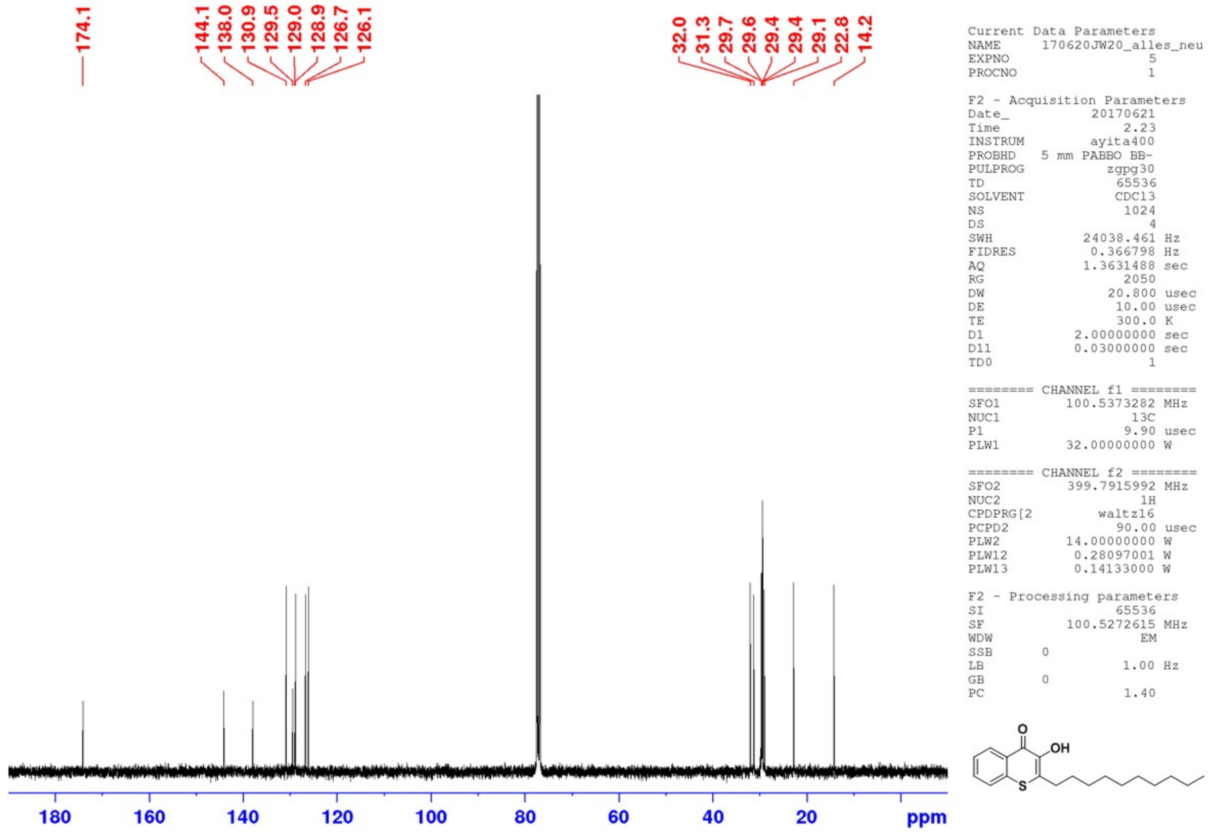

^{13}C-NMR spectra of (**8**) in $CDCl_3$

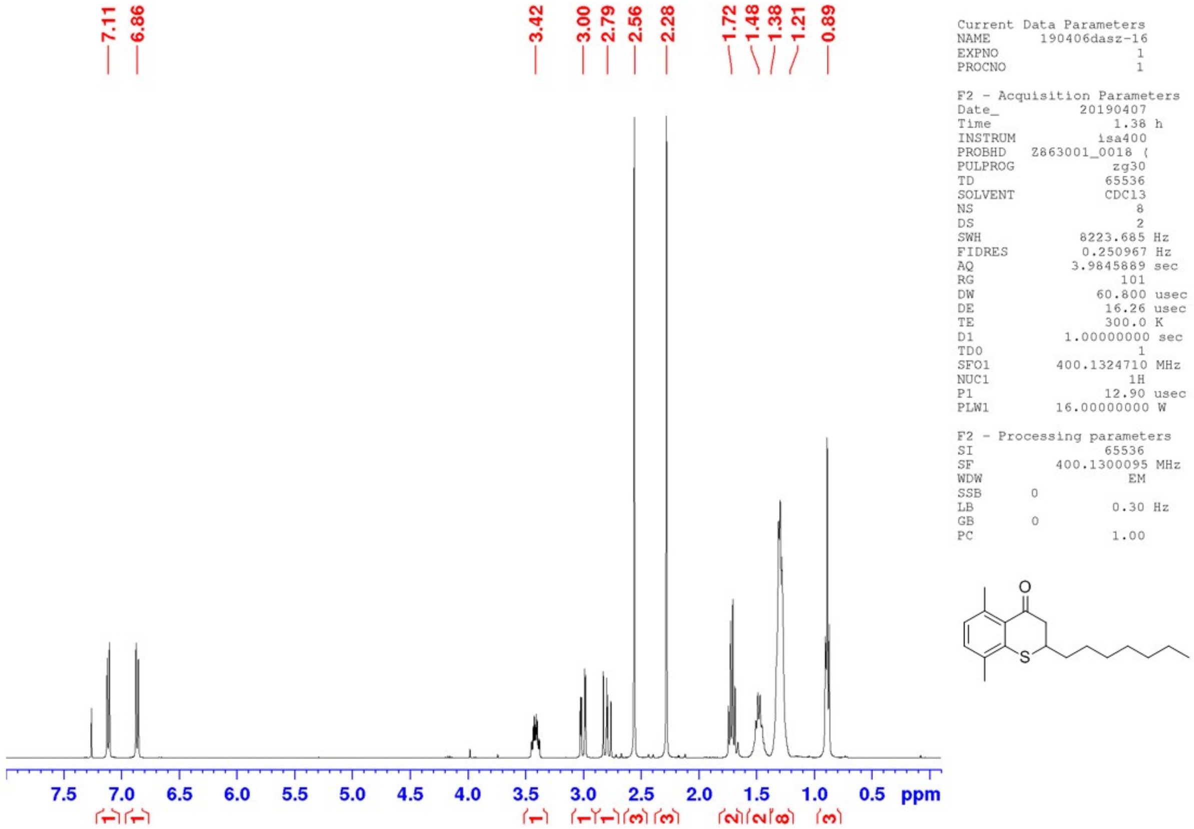

^1H-NMR spectra of (**11d**) in $CDCl_3$

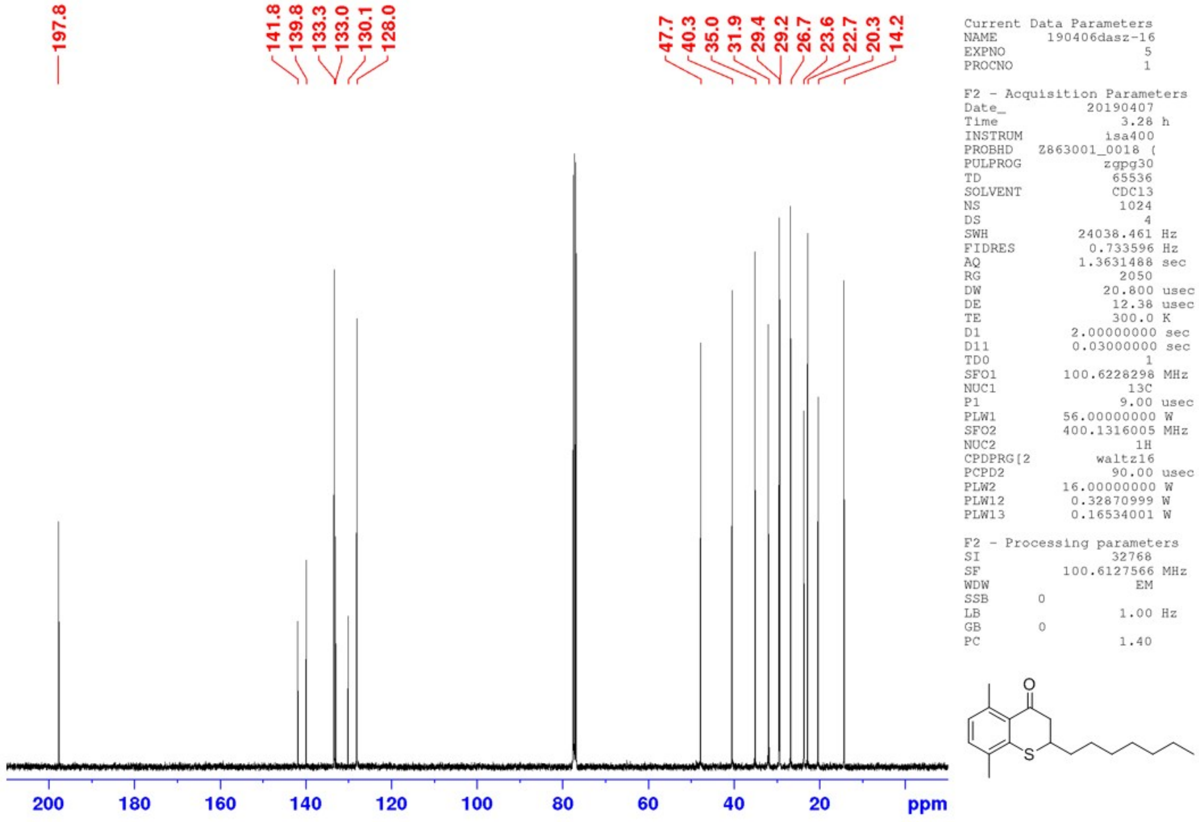

^{13}C-NMR spectra of (**11d**) in $CDCl_3$

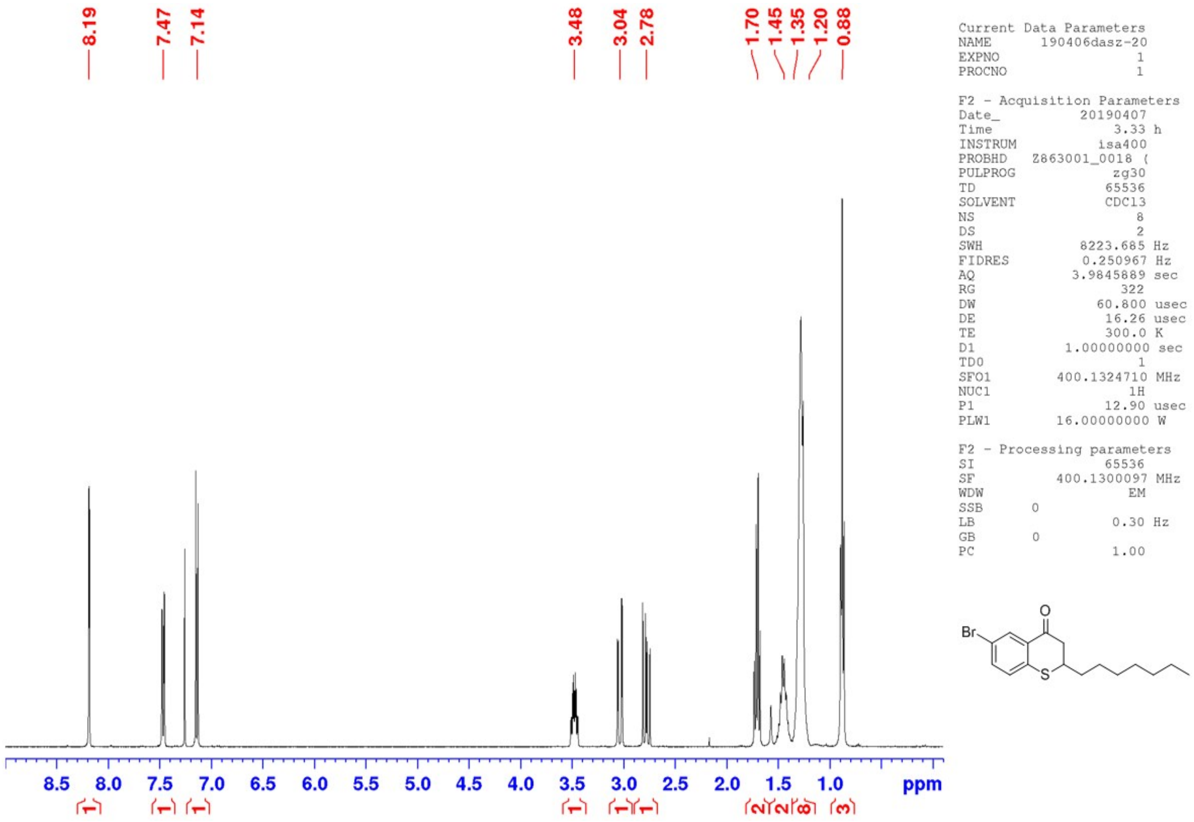

^{1}H-NMR spectra of (**12d**) in $CDCl_3$

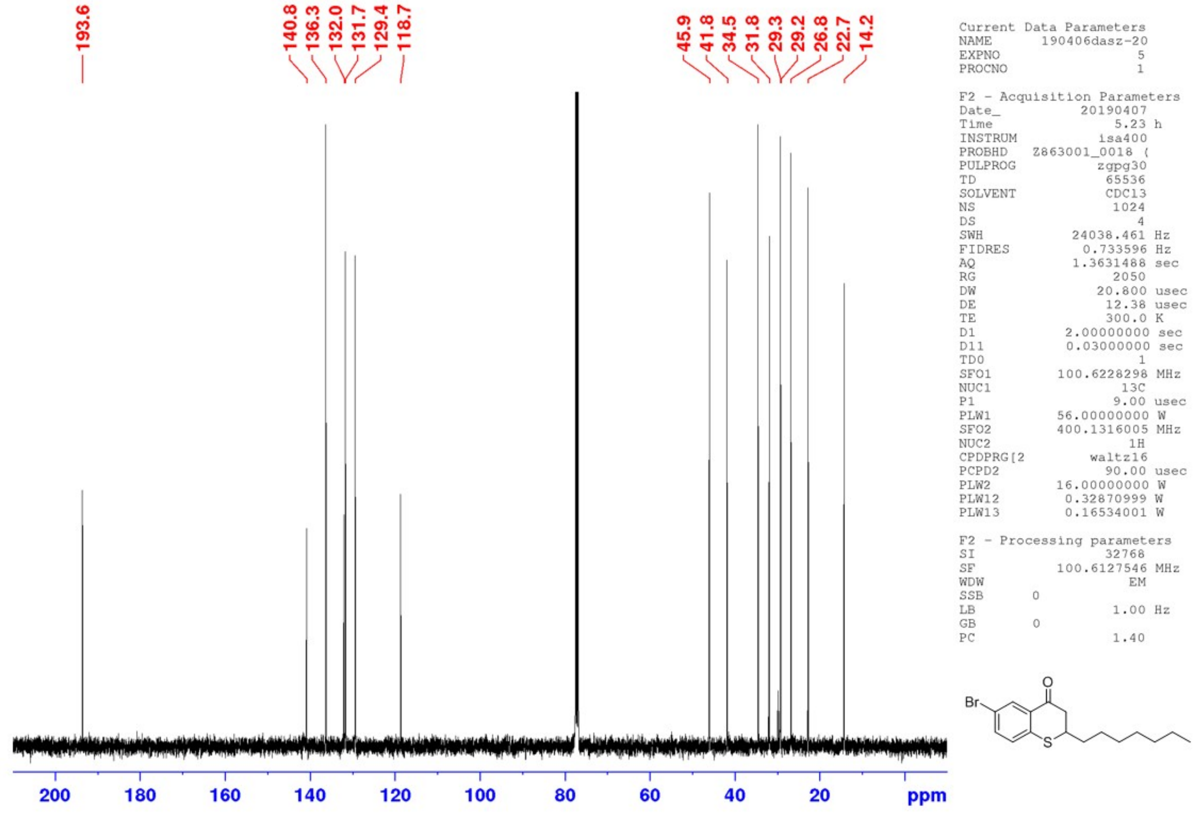

^{13}C-NMR spectra of (**12d**) in $CDCl_3$

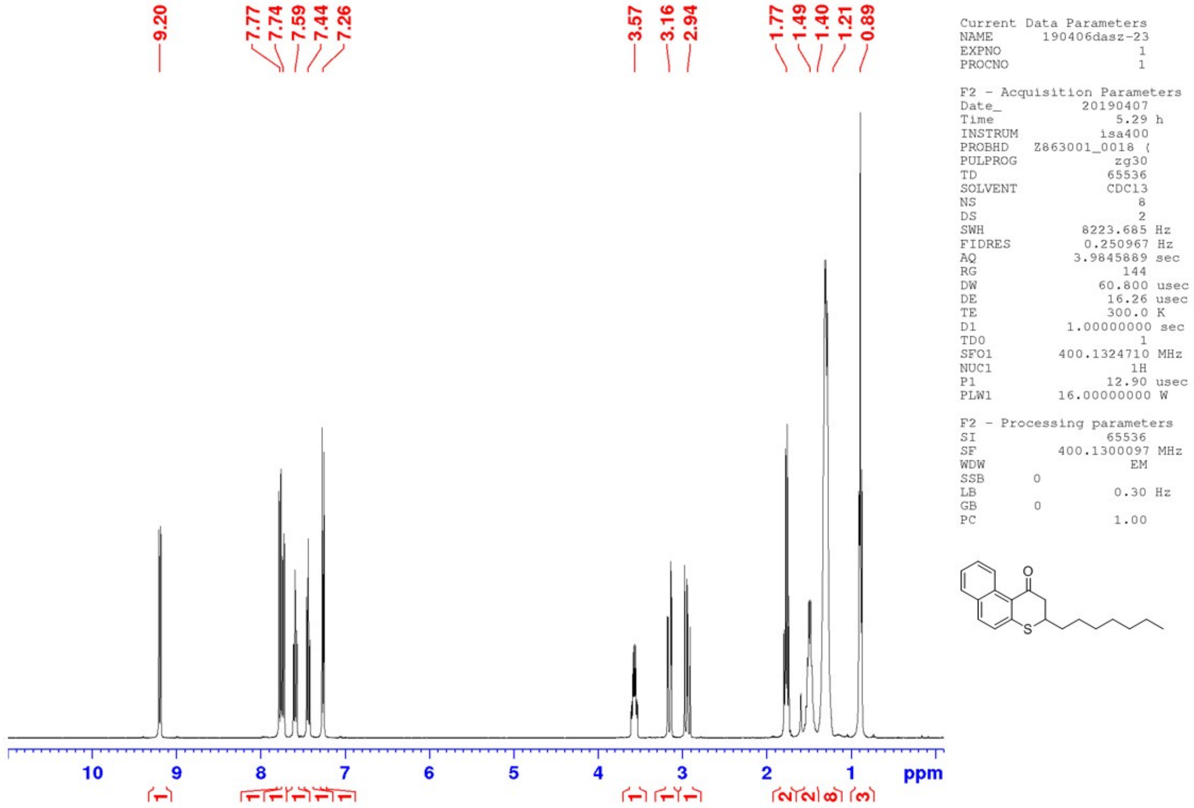

^{1}H-NMR spectra of (**13d**) in $CDCl_3$

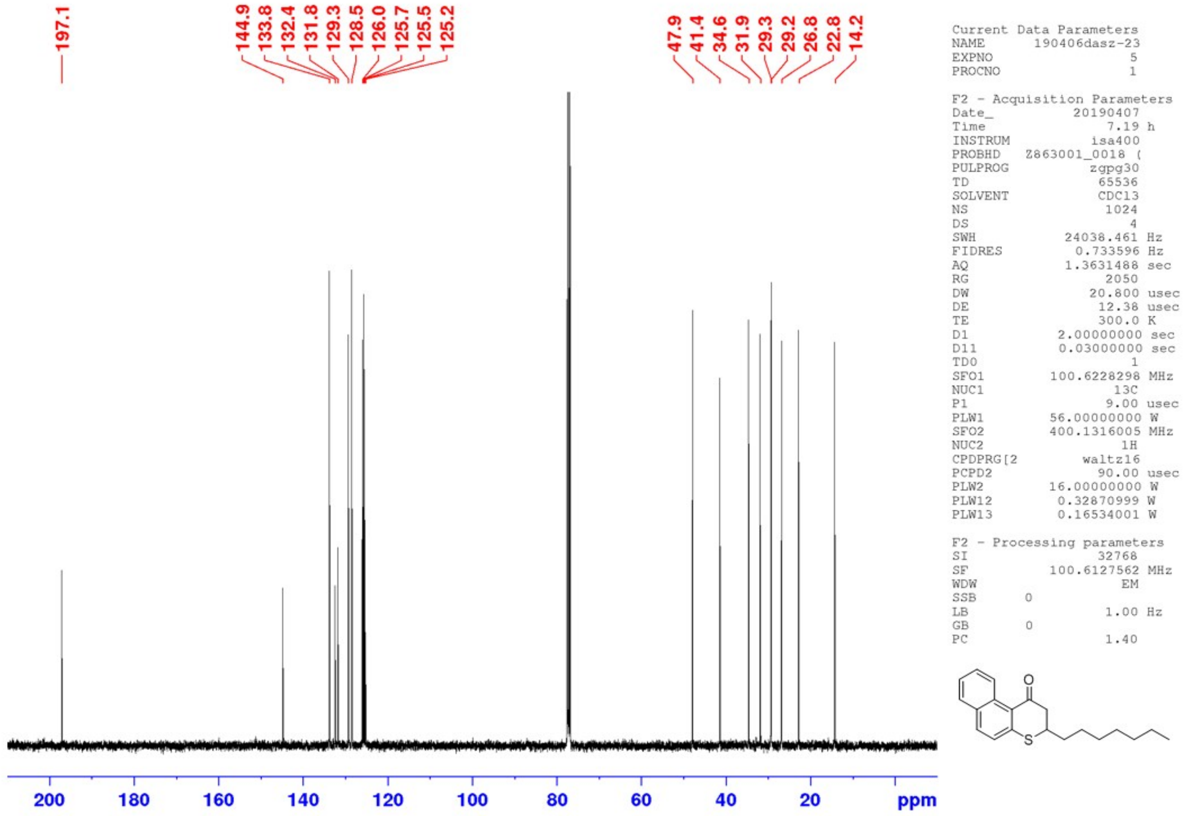

^{13}C-NMR spectra of (**13d**) in $CDCl_3$

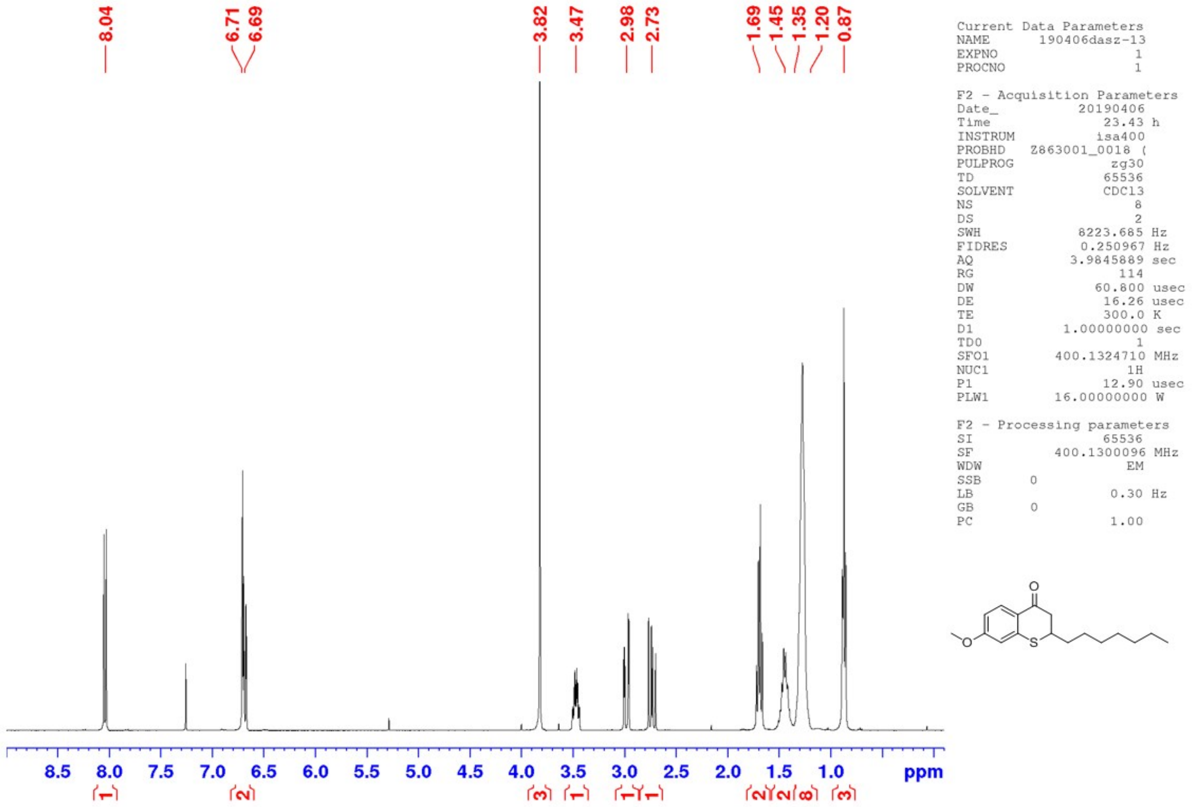

^{1}H-NMR spectra of (**14d**) in $CDCl_3$

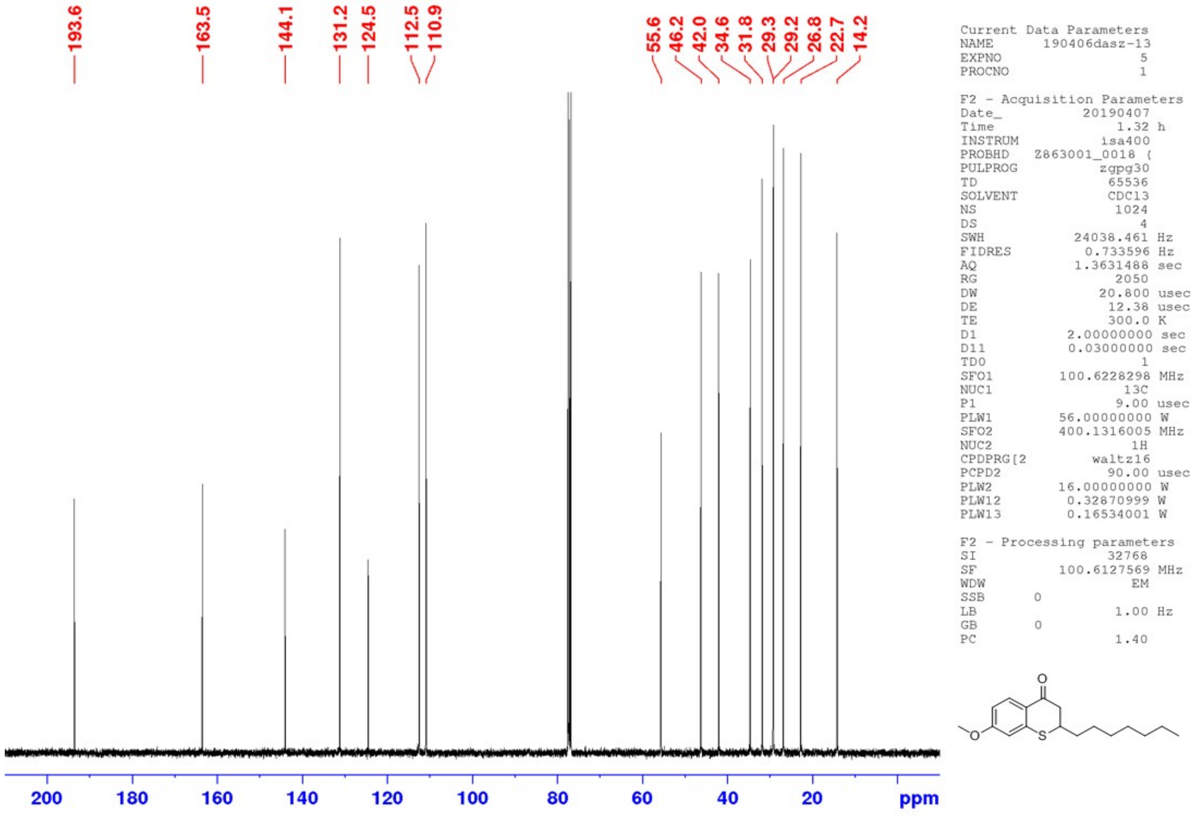

^{13}C-NMR spectra of (**14d**) in $CDCl_3$

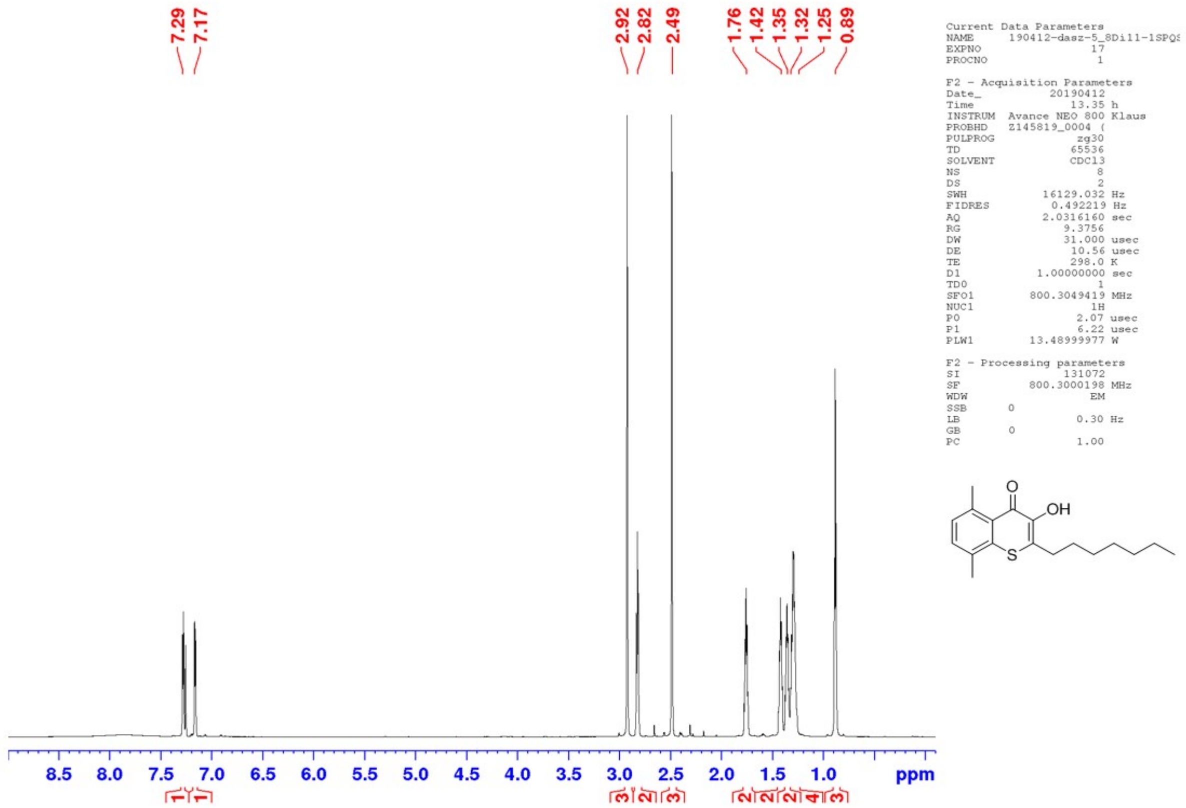

^{1}H-NMR spectra of (**11**) in $CDCl_3$

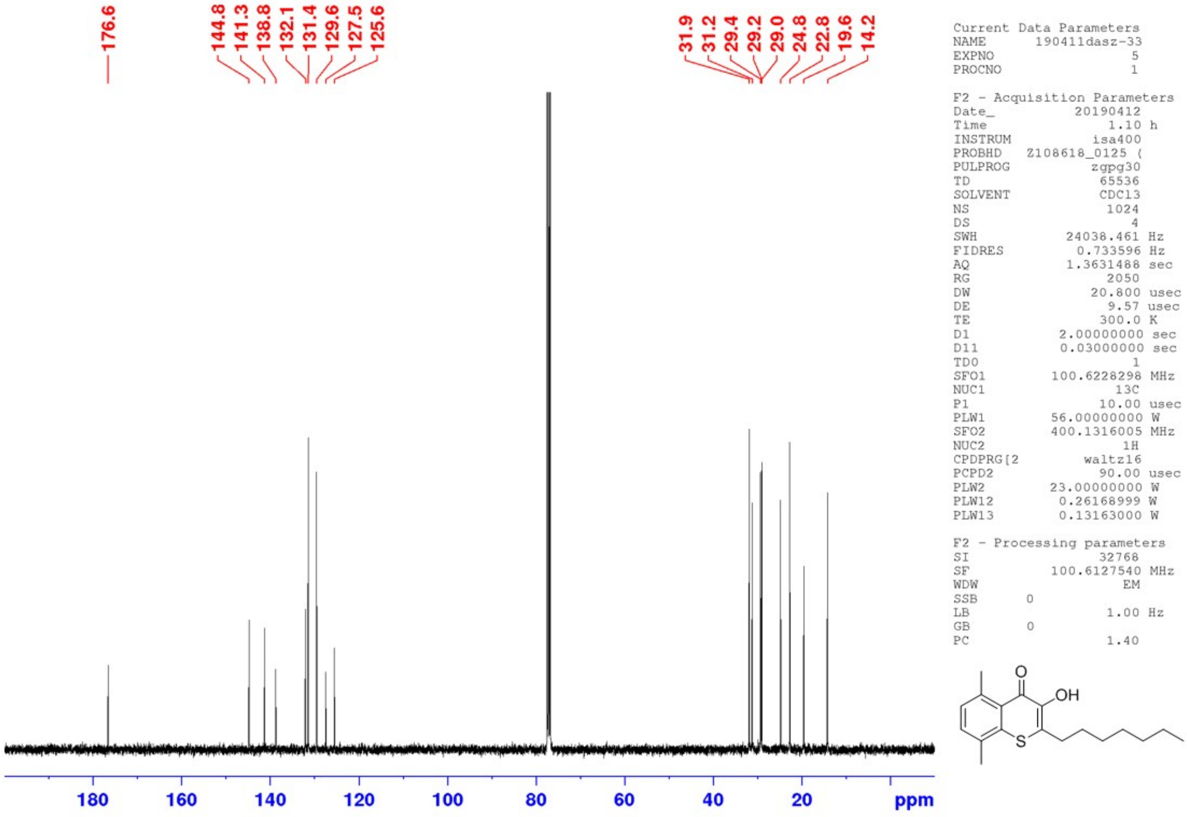

^{13}C-NMR spectra of (**11**) in $CDCl_3$

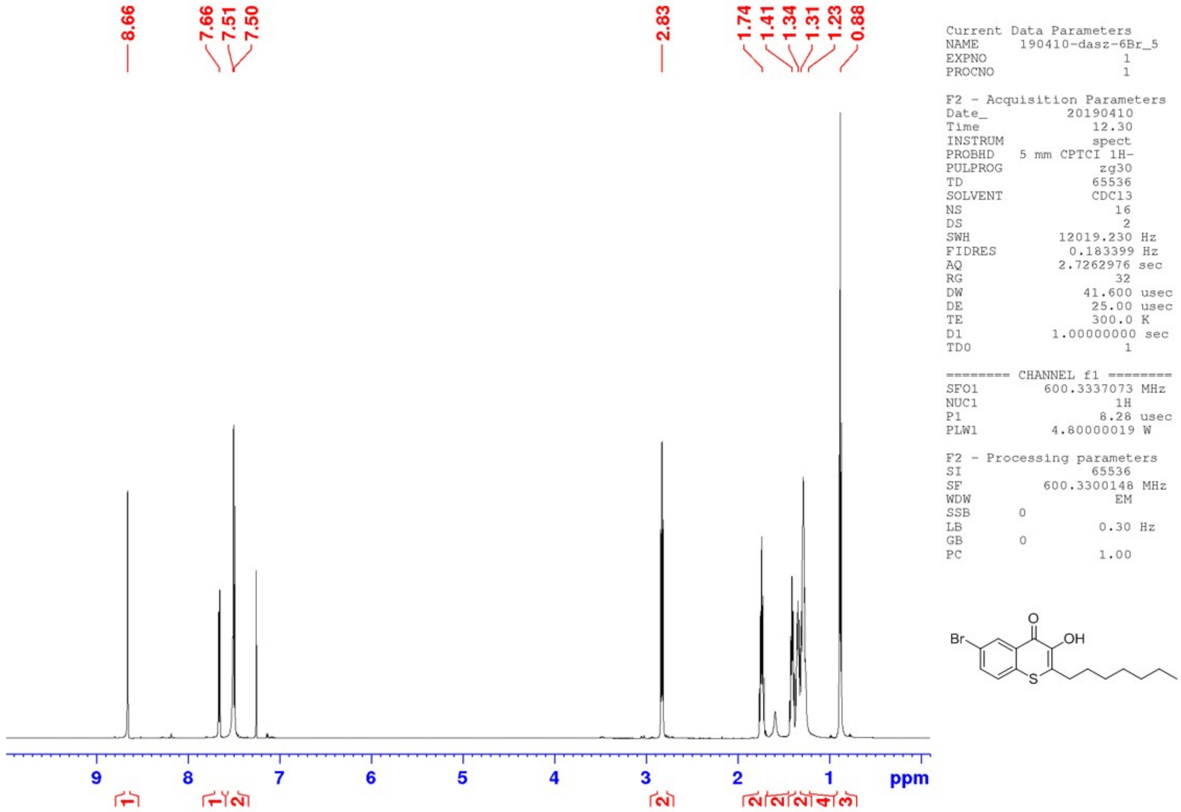

^{1}H-NMR spectra of (**12**) in $CDCl_3$

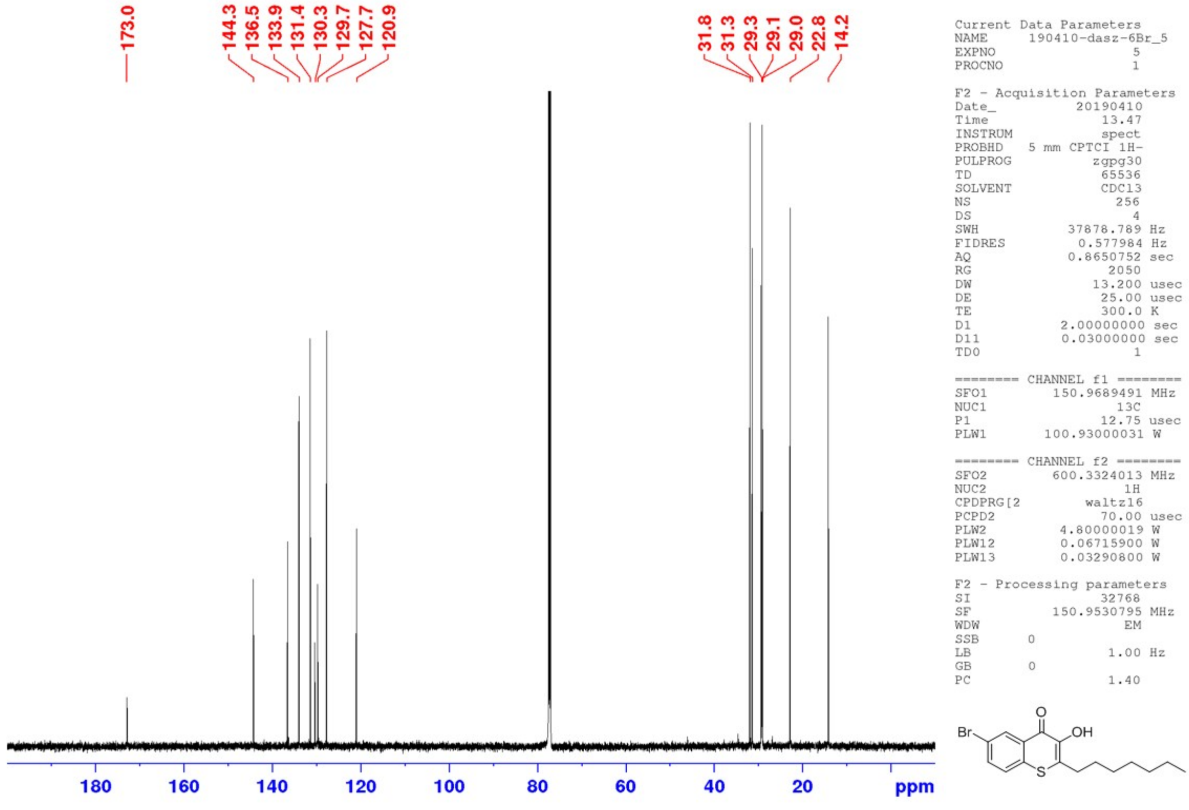

^{13}C-NMR spectra of (**12**) in $CDCl_3$

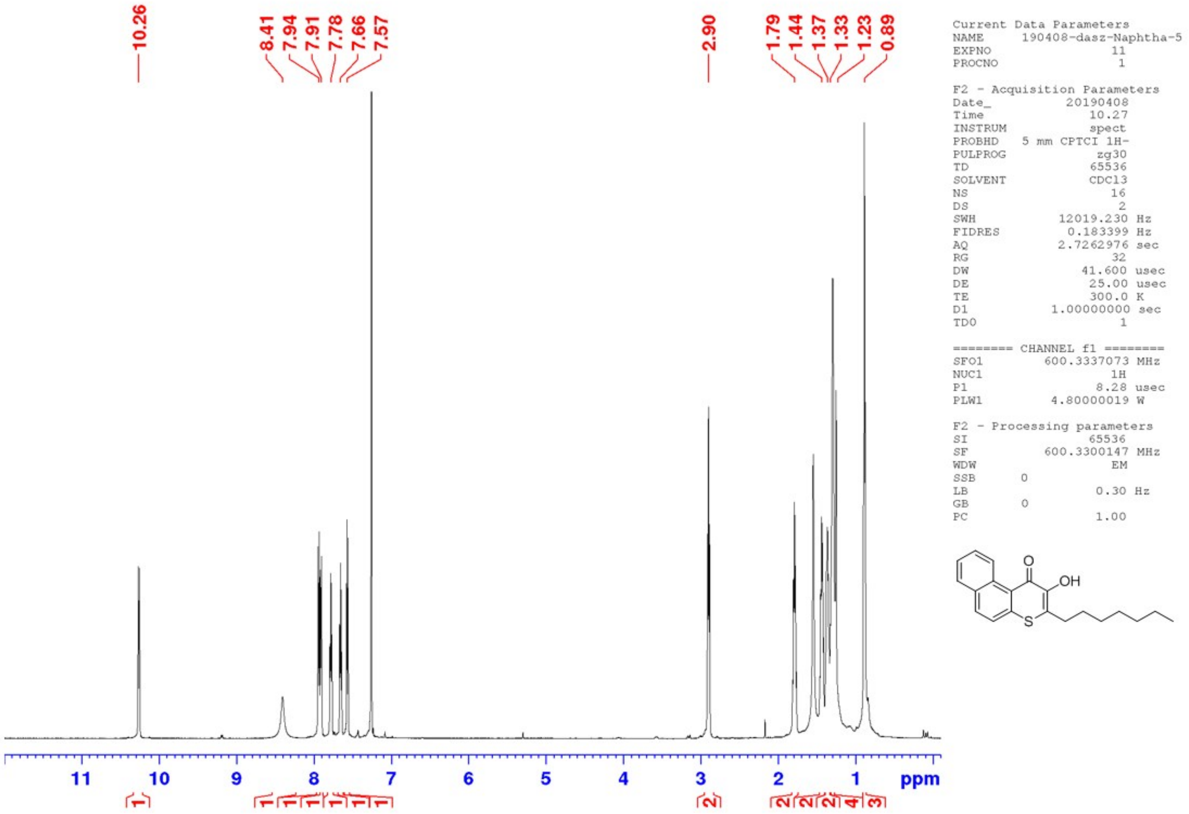

^{1}H-NMR spectra of (**13**) in $CDCl_3$

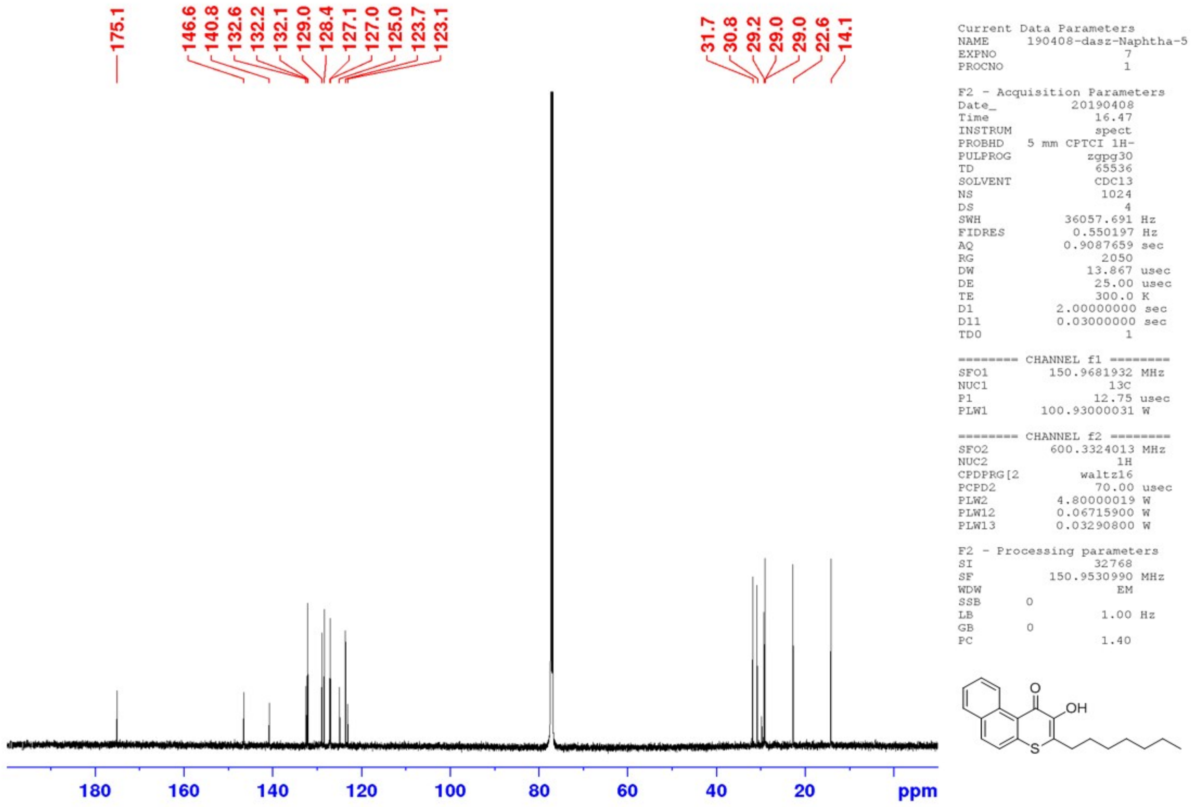

^{13}C-NMR spectra of (**13**) in $CDCl_3$

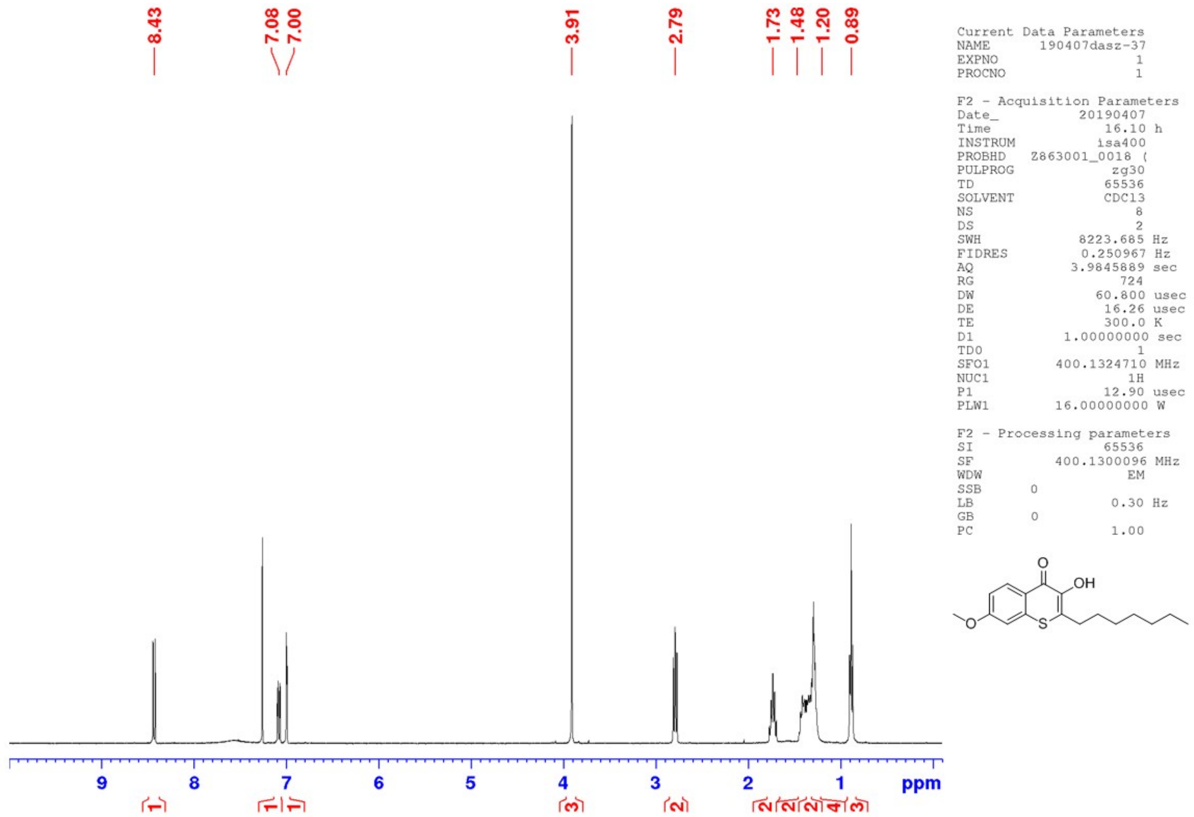

^{1}H-NMR spectra of (**14**) in $CDCl_3$

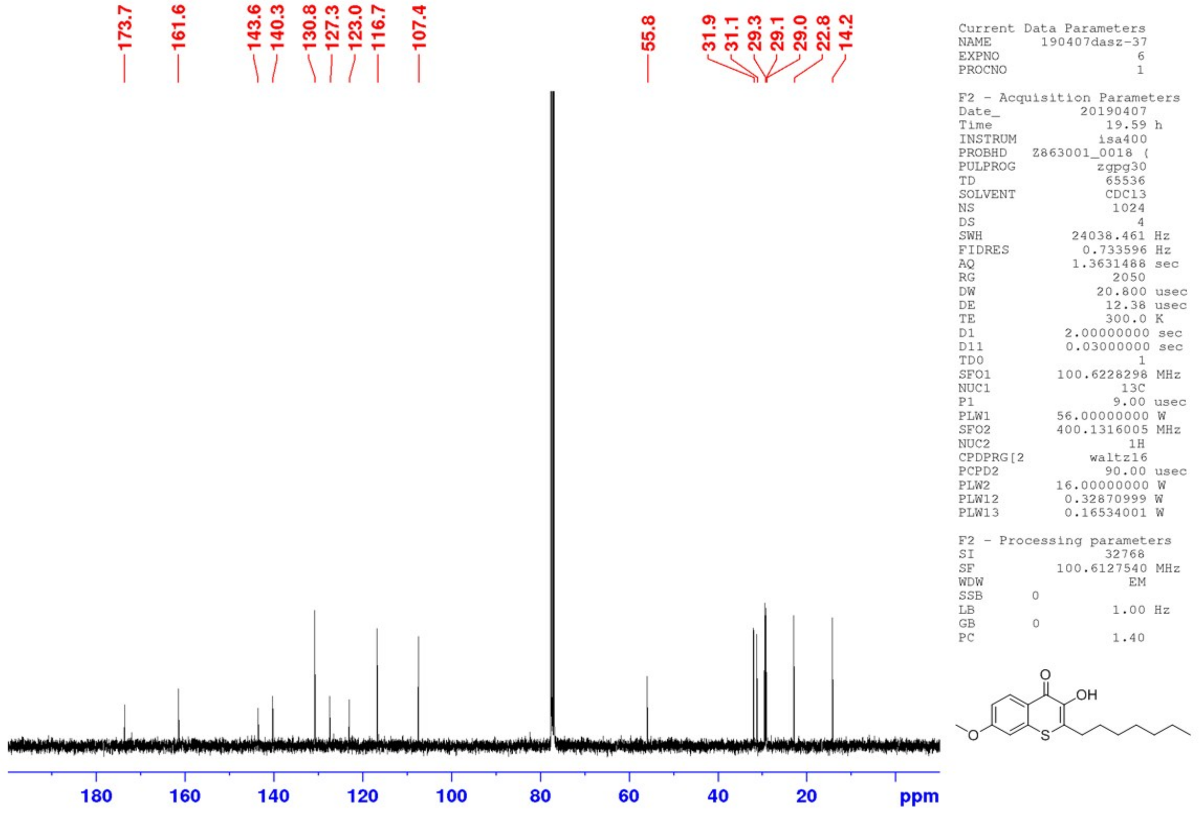

^{13}C-NMR spectra of (**14**) in $CDCl_3$
